# Computational study of the kinetics and mechanism of radical polymerization of acrylic acid and derivatives in organic solvents

**DOI:** 10.1039/d5ra09849a

**Published:** 2026-02-10

**Authors:** Mai Van Bay, Pham Thi Thuy Linh, Truong Le Bich Tram, Nguyen Thi Hoa, Adam Mechler, Quan V. Vo

**Affiliations:** a The University of Danang – University of Sciences and Education Danang 550000 Vietnam; b Department of Science and International Cooperation, The University of Danang Danang 550000 Vietnam; c The University of Danang – University of Technology and Education Danang 550000 Vietnam vvquan@ute.udn.vn; d Department of Biochemistry and Chemistry, La Trobe University Victoria 3086 Australia

## Abstract

Free-radical polymerization of acrylic acid derivatives (AADs), such as acrylic acid (AA), methyl acrylate (MA), acrylamide (AM), methacrylic acid (MAA), and methyl methacrylate (MMA), proceeds through radical addition of monomers during initiation and propagation. Despite the extensive experimental literature on this process from both a radical chemistry and an industrial point of view, there is little information on the mechanistic pathways of the radical addition process, particularly in organic solvents. In this work, quantum chemical methods were applied to explore the solvent and initiator effects on AAD polymerization. The reactivity of di-*tert*-butyl peroxide (TBO˙) and dicumyl peroxide (CMO˙) and 2-cyanoprop-2-yl radical (AI˙) as initiators was systematically studied in isopropanol (IP) and toluene (TL). Computational results predict that initiation is consistently faster in IP than in TL, with initiator efficiency ranked as TBO˙ > AI˙ > CMO˙. At 298 K, the predictions for propagation constants in IP (2.80 × 10^1^ to 2.60 × 10^4^ M^−1^ s^−1^) are substantially higher compared to those in TL (2.60–1.50 × 10^4^ M^−1^ s^−1^). Additionally, solvent-derived radicals were predicted to participate actively in propagation. Temperature was found to significantly increase log(*k*_p_) in both toluene and isopropanol, consistent with the Arrhenius kinetic model. The computed propagation rate constants for MA polymerization in toluene (1.00 × 10^3^ to 1.10 × 10^5^ M^−1^ s^−1^ at 320 K) and the activation energies of MA and MMA (3.6 and 3.4 kcal mol^−1^, respectively) align well with experimental results, validating the accuracy and reliability of the computational approach. The effect is more pronounced in toluene due to the absence of hydrogen-bonding interactions, underscoring the key role of the solvent in controlling propagation kinetics. This study predicts that solvent polarity and the properties of the radical strongly govern the kinetics of AAD polymerization, thereby providing useful mechanistic insights for optimizing radical polymerization in non-aqueous media.

## Abstract 2

Computational results confirmed the radical polymerization of AADs in organic solvents, yielding propagation rate constants of 10^1^–10^4^ M^−1^ s^−1^.

## Graphical abstract

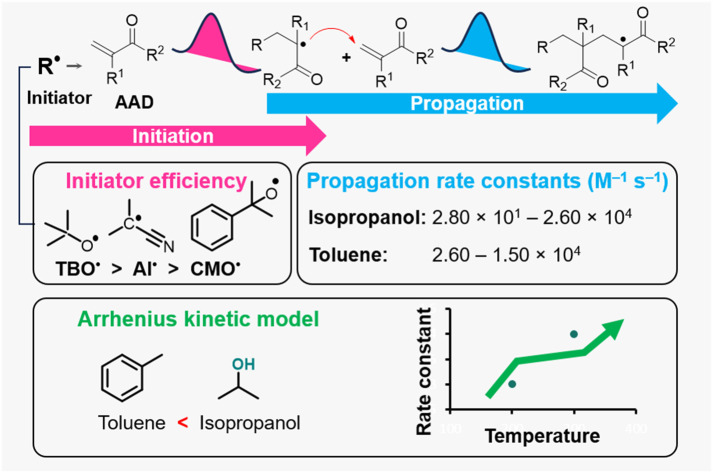

## 1. Introduction

**Fig. 1:**
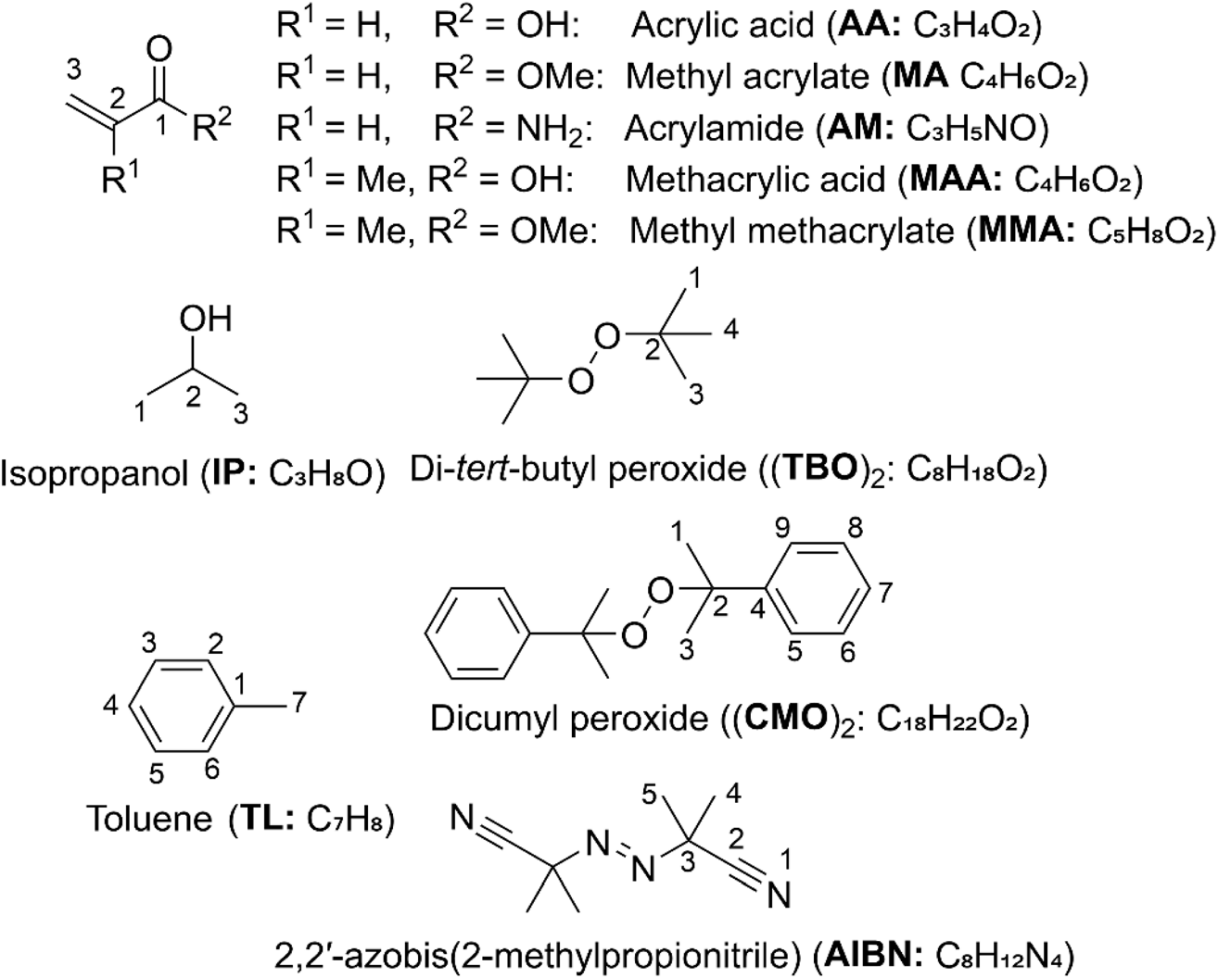
Structure, molecular formulas, and numbering of AAD, (TBO)_2_, (CMO)_2_, AIBN, IP and TL.

In the free-radical polymerization of acrylic acid derivatives (AADs; Fig. 1), such as AA, MA, AM, MAA and MMA, the initiation and propagation steps occur *via* radical addition to the monomeric double bonds. Polymerization is routinely carried out in aqueous media^1–9^ or in organic solvents like isopropanol (IP) and toluene (TL), using organic peroxides such as dicumyl peroxide (CMO)_2_ and di-*tert*-butyl peroxide (TBO)_2_, or azo initiators like 2,2′-azobis(2-methylpropionitrile) (AIBN) as radical sources (Fig. 1).^10–20^ Recent studies used purpose-designed modeling and simulation frameworks, introduced as a “*digital-twin*” approach, to investigate free-radical polymerization processes.^21–23^ These works reverted to unrestricted Hartree–Fock formalism with a semi-empirical quantum chemistry framework to allow the simulation of the polymerization process as a system of multiple possibilities, delivering qualitative but not quantitative insights into spin-related effects in the polymerization and copolymerization of vinyl monomers with stable radicals. Functionalization of polyolefins with AA and its derivatives using peroxide initiators—particularly dicumyl peroxide and di-*tert*-butyl peroxide (TBO)—has proven to be an effective approach for controlling polymer properties, enhancing grafting efficiency, as well as thermomechanical and surface characteristics.^12,13,16–20^ AIBN has been used in several works as an initiator for the radical polymerization of AA and its derivatives.^11,14,15,24^ However, mechanistic and kinetic investigations on the polymerization of AADs initiated by activators such as (CMO)_2_, (TBO)_2_, and AIBN remain unavailable. Therefore, a comprehensive study is essential to elucidate their polymerization mechanisms and reaction kinetics.

The experimental rate constant of MA in toluene was reported as *k*_p_(MA) = 1.47 × 10^4^ and 2.82 × 10^4^ M^−1^ s^−1^ at 293 and 323 K, respectively,^25,26^ and by others as 1.03 × 10^4^ to 3.31 × 10^4^ M^−1^ s^−1^ in the temperature range of 285–354 K.^27^ The radical polymerization of MA was found to follow the Arrhenius kinetic model, with activation energies (*E*_a_) of 4.1 (ref. 28) and 4.4 kcal mol^−1^,^27^ whereas that of MMA in toluene ranged from 2.8 to 5.3 kcal mol^−1^ within 303–333 K.^24^ Several studies have also found that the radical polymerization of AA in TL and IP obeys the Arrhenius kinetic model;^11,14,15^ however, comprehensive investigations on the kinetics of AADs have not yet been reported.

Although numerous studies have examined polymerization processes in selected solvent media,^3,10–15,21,24,29^ none of these delved into a comprehensive investigation of the initiation mechanism and propagation kinetics. Moreover, the reports of the solvent effects on the polymerization reactions are also inconsistent. Haehnel *et al.*^25^ observed that the propagation rate (*k*_p_) was largely unaffected by organic solvents such as butyl acetate and toluene, even under conditions comparable to bulk polymerization with the increasing number of C atoms in their ester side chain. Yet a range of other studies reported solvent-dependent behavior.^28,30–33^ Nonetheless, a consistent kinetic trend has been established, indicating that *k*_p_ systematically increases with the elongation of the ester side chain under all examined conditions.^28,30,31^ Several studies indicated that solvents may actively participate in the initiation step, thereby contributing to the characteristics of the resulting polymers.^3,34,35^ The temperature dependence of polymerization in various solvents has also been recognized as a crucial factor.^24^ Yet, these aspects have not been addressed comprehensively for the polymerization reactions of AADs.

Building on our previous investigations into the radical polymerization of AADs in aqueous media,^36^ the present work employs a quantum chemistry-based approach^37–39^ to explore the polymerization behavior of acrylic acid derivatives initiated by alkoxy radicals (TBO˙ and CMO˙) and the azo radical (2-cyanoprop-2-yl, AI˙) in isopropanol (IP) and toluene (TL) solvents.

## 2. Computational details

The kinetic evaluation was carried out using the quantum mechanics-based test for the overall free radical scavenging activity (QM-ORSA) protocol.^37^ This protocol combines density functional theory calculations of reaction energetics with transition state theory to estimate kinetic parameters in solution, and it is broadly applicable to all radical processes due to their mechanistic similarity.^40,41^ Rate constants (*k*) were determined *via* transition state theory (TST) at 298.15 K and 1 M standard state (eqn (1)).^42–46^1
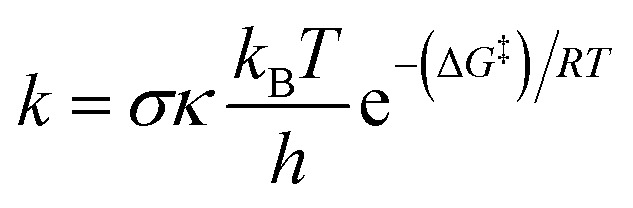
Here, *σ* is the reaction symmetry factor,^47,48^*κ* is the tunneling correction from the Eckart barrier^49^ calculated using the Eyringpy code,^45,46^*k*_B_ and *h* are Boltzmann and Planck constants, and Δ*G*^‡^ is the Gibbs free energy of activation.

Reactions approaching the diffusion limit were corrected according to the Collins–Kimball treatment,^50^ yielding apparent rate constants (*k*_app_) as shown in eqn (2).^40,51^ Recent studies have validated this model for diffusion-controlled reactions in various solvents.^36,37,39–41,52–56^2
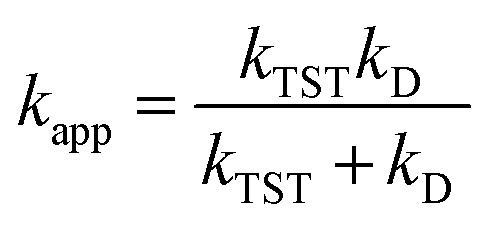

3 *k*_D_ = 4π*R*_AB_*D*_AB_*N*_A_

Diffusion coefficients (*D*_AB_ = *D*_A_ + *D*_B_) were obtained by the Stokes–Einstein equation (eqn (4)), using solvent viscosities of 20.4 × 10^−4^ Pa s (isopropanol) and 5.60 × 10^−4^ Pa s (toluene).^50,57–59^4
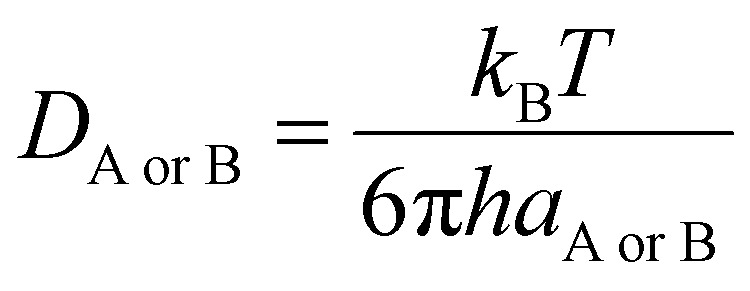


Transition states were verified by single imaginary frequencies and confirmed by IRC calculations. All computations were performed at the M06-2X/6-311++G(d,p) level of theory, which provides reliable thermodynamic and kinetic predictions.^36,39,52,60–63^ The solvent effects were modeled using SMD.^64^ This approach is considered dependable for radical reaction studies, as the calculated-to-experimental rate constant ratios remain within 0.2–2.9.^38–40,45,65^

Rate constants at different temperatures (293–353 K for IP and 280–380 K for TL) were calculated using eqn (1), in which the Gibbs free energy of activation, Δ*G*^‡^(*T*), was evaluated according to eqn (5).

5 Δ*G*^‡^(*T*) = Δ*H*^‡^(*T*) − *T*Δ*S*^‡^(*T*)

Here, Δ*H*^‡^ and Δ*S*^‡^ denote the enthalpy and entropy of activation (kcal mol^−1^), respectively, obtained in the solvent phase for IP and TL using the SMD solvation model at 298.15 K assumed to be temperature independent within the examined temperature range. The kinetic parameters were calculated with the Eyringpy program.^45,46^ While approximate, this approach is widely used in both experimental and theoretical kinetic studies.^66–69^ The calculations were performed with Gaussian 16.^70^

## 3. Results and discussion

### 3.1. Initiation reactions of TBO˙/CMO˙ in the organic solvents

**Fig. 2:**
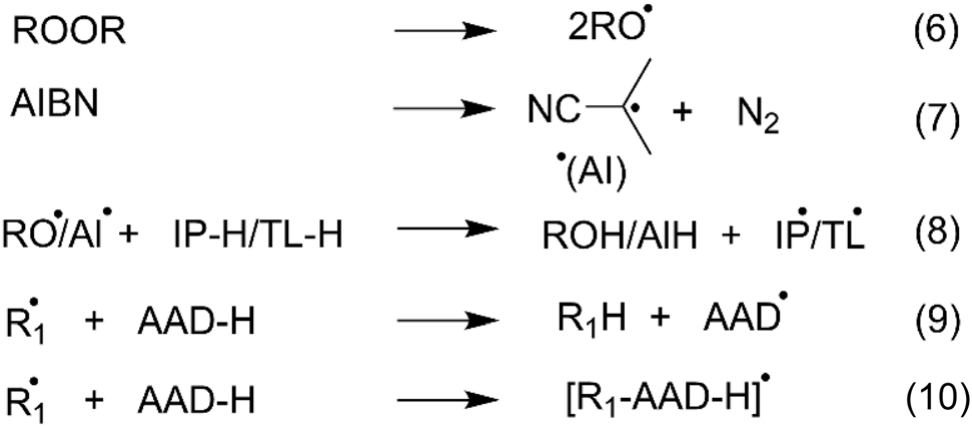
The initiation reaction of the TBO˙/CMO˙/AI˙ in isopropanol (IP) and toluene (TL) 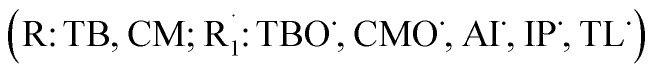.

**Fig. 3:**
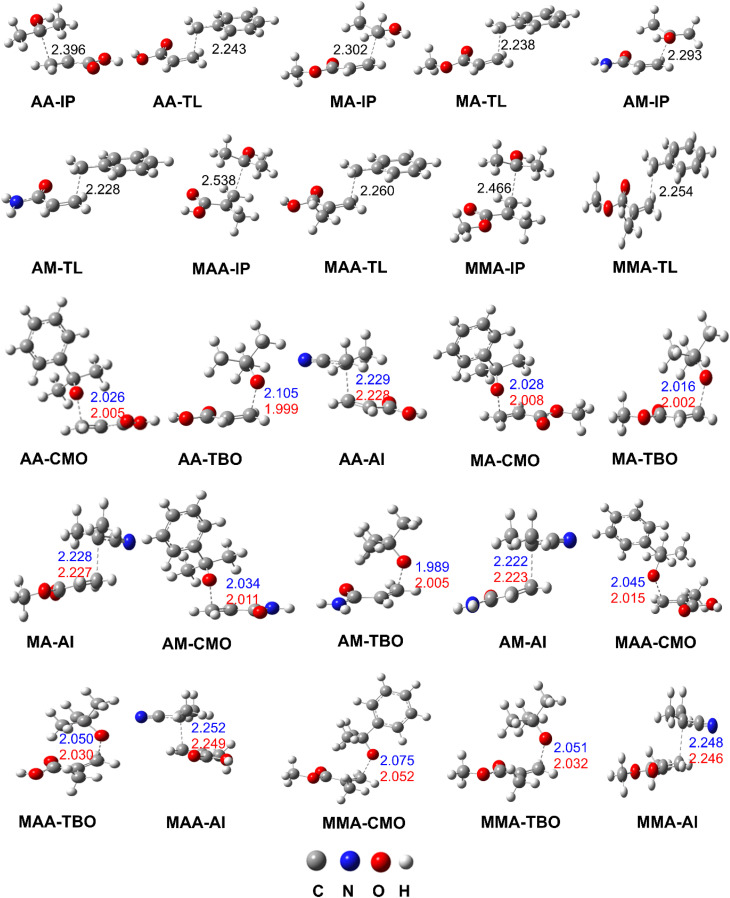
Selected transition states of the reactions (green for IP and red for TL solvents).

To explore the initiation stage of polymerization triggered by organic peroxides, including di-*tert*-butyl peroxide ((TBO)_2_), dicumyl peroxide ((CMO)_2_), and 2,2′-azobis(2-methylpropionitrile) (AIBN), in organic solvents such as IP or TL, the possible radical pathways were considered as described in reactions (6)–(10) (Fig. 2). Alkoxy radicals are produced *via* thermal decomposition of peroxides (reaction (6)), while decomposition of AIBN generates the 2-cyanoprop-2-yl radical (AI˙) through reaction (7). Our previous findings demonstrated that TBO˙ and CMO˙ preferentially undergo formal hydrogen transfer (FHT) with IP and TL, yielding the solvent-derived radicals IP–C2˙ and TL–C7˙, respectively.^54^ In contrast, reactions of AI˙ with these solvents are thermodynamically unfavorable due to positive Gibbs free energies (Table S1, SI). Consequently, in IP and TL media, initiation involves interactions between CMO˙, TBO˙, and AI˙ (from the initiators) as well as solvent-derived IP–C2˙ and TL–C7˙ radicals, reacting with AADs through radical adduct formation (RAF) predominantly at the C2 and C3 positions. The kinetics of the AADs + IP–C2˙/TL–C7˙/TBO˙/CMO˙/AI˙ reactions were subsequently evaluated, and the results are summarized in Table 1 and Fig. 3.

**Table 1:** Calculated Δ*G*^‡^ (kcal mol^−1^), tunneling corrections (*κ*), rate constants (*k*_app_ and *k*_overall_ M^−1^ s^−1^) and branching ratios (*Γ*, %) for the AAD + IP–C2˙/TL–C7˙/TBO˙/CMO˙/AI˙ reactions in the organic solvents

Comp.	Radicals	Positions	TL				IP			
			Δ*G*^‡^	*κ*	*k*_app_	*Γ*	Δ*G*^‡^	*κ*	*k*_app_	*Γ* a
AA	TL–C7˙/IP–C2˙	C2	18.3	1.5	3.80 × 10^−1^	0.1	11.7	1.2	1.90 × 10^4^	0.0
		C3	13.9	1.3	5.40 × 10^2^	99.9	6.6	1.0	7.83 × 10^7^	100.0
		***k***_**overall**_			**5.40 × 10** ^**2**^				**7.84 × 10** ^**7**^	
	CMO˙	C2	19.6	1.5	3.7 × 10^−2^	2.0	18.0	1.4	5.30 × 10^−1^	3.0
		C3	17.3	1.4	1.80	98.0	16.0	1.4	1.70 × 10^1^	97.0
		***k***_**overall**_			**1.84**				**1.75 × 10** ^**1**^	
	TBO˙	C2	18.0	1.5	6.20 × 10^−1^	0.8	16.9	1.5	3.50	4.3
		C3	15.1	1.5	7.30 × 10^1^	99.2	15.1	1.5	7.70 × 10^1^	95.7
		***k***_**overall**_			**7.36 × 10** ^**1**^				**8.05 × 10** ^**1**^	
	AI˙	C2	21.7	1.5	1.20 × 10^−3^	0.0	20.5	1.5	8.10 × 10^−3^	0.0
		C3	15.9	1.3	1.90 × 10^1^	100.0	15.1	1.3	7.10 × 10^1^	88.2
		***k***_**overall**_			**1.90 × 10** ^**1**^				**7.10 × 10** ^**1**^	
MA	TL–C7˙/IP–C2˙	C2					11.5	1.1	2.40 × 10^4^	29.6
		C3	14.4	1.3	2.40 × 10^2^	100.0	11.0	1.0	5.70 × 10^4^	70.4
		***k***_**overall**_			**2.40 × 10** ^**2**^				**8.10 × 10** ^**4**^	
	CMO˙	C3	17.4	1.4	1.70	100.0	15.7	1.4	2.60 × 10^1^	100.0
	TBO˙	C3	15.3	1.5	5.60 × 10^1^	100.0	14.8	1.5	1.40 × 10^2^	100.0
	AI˙	C3	15.9	1.4	1.70 × 10^1^	100.0	15.3	1.3	5.30 × 10^1^	100.0
AM	TL–C7˙/IP–C2˙	C2					12.5	1.2	5.10 × 10^3^	26.8
		C3	14.5	1.3	1.80 × 10^2^	100.0	12.2	1.0	7.90 × 10^3^	41.5
		***k***_**overall**_			**1.80 × 10** ^**2**^				**1.30 × 10** ^**4**^	
	CMO˙	C3	17.9	1.5	7.30 × 10^−1^	100.0	16.0	1.4	1.80 × 10^1^	100.0
	TBO˙	C3	15.2	1.5	6.80 × 10^1^	100.0	15.2	1.5	7.10 × 10^1^	100.0
	AI˙	C3	16.4	1.4	7.50	100.0	15.8	1.4	2.30 × 10^1^	100.0
MAA	TL–C7˙/IP–C2˙	C2					13.5	1.2	9.90 × 10^2^	0.0
		C3	13.6	1.3	8.20 × 10^2^	100.0	6.5	1.0	9.17 × 10^7^	100.0
		***k***_**overall**_			**8.20 × 10** ^**2**^				**9.17 × 10** ^**7**^	
	CMO˙	C3	17.7	1.5	9.50 × 10^−1^	100.0	15.8	1.4	2.30 × 10^1^	100.0
	TBO˙	C3	14.0	1.4	4.70 × 10^2^	100.0	13.5	1.4	1.10 × 10^3^	100.0
	AI˙	C3	17.5	1.4	1.30	100.0	14.3	1.3	2.60 × 10^2^	100.0
MMA	TL–C7˙/IP–C2˙	C2					13.4	1.2	1.10 × 10^3^	0.0
		C3	14.1	1.3	4.00 × 10^2^	100.0	7.9	1.0	9.52 × 10^6^	100.0
		***k***_**overall**_			**4.00 × 10** ^**2**^				**9.52 × 10** ^**6**^	
	CMO˙	C3	16.1	1.4	1.40 × 10^1^	100.0	15.5	1.3	3.40 × 10^1^	100.0
	TBO˙	C3	14.1	1.4	3.70 × 10^2^	100.0	13.8	1.4	7.20 × 10^2^	100.0
	AI˙	C3	17.4	1.4	1.50	100.0	14.9	1.3	9.90 × 10^1^	100.0

a *Γ* = *k*_app_ × 100/*k*_overall_.

The modelling of initiation reactions in the TL solvent predicted pronounced differences among the monomers (AADs) and radical species (TL–C7˙/TBO˙/CMO˙/AI˙). For AA, the overall reactivity with the solvent-derived radical (TL–C7˙) was predicted to be dominant (*k*_app_ = 5.40 × 10^2^ M^−1^ s^−1^), occurring almost exclusively at the C3 position (99.9%). In contrast, the primary initiator radicals CMO˙, TBO˙, and AI˙ were far less efficient, with *k*_app_ values of only 1.84, 73.6, and 19.0 M^−1^ s^−1^, respectively. A similar trend was observed for MA and AM, where the reactions preferentially occurred at the C3 site. The highest *k*_app_ values were again associated with the solvent radical (2.40 × 10^2^ M^−1^ s^−1^ for MA and 1.80 × 10^2^ M^−1^ s^−1^ for AM), while TBO˙ and CMO˙ displayed only moderate reactivity. Notably, the AI˙ radical was predicted to show only modest reactivity toward MA (17.0 M^−1^ s^−1^) and AM (7.50 M^−1^ s^−1^), highlighting significant kinetic limitations compared with the TL–C7˙ radical. For MAA and MMA, the differences were even more pronounced. The TL–C7˙ and TBO˙ predicted to have the highest reactivities (8.20 × 10^2^ and 4.00 × 10^2^ M^−1^ s^−1^, respectively), greatly surpassing those of CMO˙ (0.95 and 14.0 M^−1^ s^−1^) and AI˙ (1.3 and 1.5 M^−1^ s^−1^), respectively.

Comparison across monomers in the TL solvent for each radical suggests that initiation reactions were specific to each AAD. For instance, the TL–C7˙ radical is predicted to react most rapidly with MAA (*k*_app_ = 8.20 × 10^2^ M^−1^ s^−1^), followed by AA (5.40 × 10^2^ M^−1^ s^−1^), MMA (4.00 × 10^2^ M^−1^ s^−1^), MA (2.40 × 10^2^ M^−1^ s^−1^), and AM (1.80 × 10^2^ M^−1^ s^−1^). By contrast, the TBO˙ radical is predicted to show highest reactivity with MMA (3.70 × 10^2^ M^−1^ s^−1^), followed by AA (7.36 × 10^1^ M^−1^ s^−1^), AM (6.80 × 10^1^ M^−1^ s^−1^), MA (5.60 × 10^1^ M^−1^ s^−1^), and MAA (4.70 × 10^1^ M^−1^ s^−1^). Both CMO˙ and AI˙ were consistently less effective, with rate constants in the range of 0.73–1.90 × 10^1^ M^−1^ s^−1^.

In the IP solvent IP–C2˙ is predicted to have the highest reaction rates for all monomers, ranging from 10^4^ to 10^7^ M^−1^ s^−1^, far exceeding the other radicals. It was noted that the calculated *k*_app_ values were the highest for MAA and AA with *k*_app_ = 7.84 × 10^7^ and 9.17 × 10^7^ M^−1^ s^−1^, respectively, further confirming that the initiation reactions in the IP medium are strongly dominated by the solvent radical (IP–C2˙). Among the primary initiators, TBO˙ showed the highest activity (up to 1.10 × 10^3^ M^−1^ s^−1^ for MAA and 7.20 × 10^2^ M^−1^ s^−1^ for MMA), with the AADs compared with the CMO˙ and AI˙ radicals. By contrast, the CMO˙ and AI˙ were less effective initiators, with *k*_app_ values generally limited to the range of 10^1^–10^2^ M^−1^ s^−1^. These findings predict that the solvent radical (IP–C2˙) plays a decisive role in the initiation mechanism of acrylic monomers, while substituents such as –NH_2_, –COOH, and –CH_3_ exert significant influence on the reaction kinetics. Among the studied compounds, MAA was predicted to exhibit the highest reactivity in the IP solvent.

The initiation reactions proceed more rapidly in IP than in TL for all investigated AADs and radicals (IP–C2˙, TL–C7˙, TBO˙, CMO˙, and AI˙). Solvent-derived radicals (IP–C2˙ and TL–C7˙) are predicted to have higher reactivity toward AADs compared with the primary initiators (TBO˙, CMO˙, and AI˙). These results suggest that the solvents, *i.e.*, IP and TL, have a strong influence on the polymerization of the AADs initiated by alkoxy radicals, such as TBO˙ and CMO˙. Consequently, the resulting polymers are likely to incorporate structural fragments derived from the solvent molecules (IP or TL). Among the investigated initiators, the relative efficiency follows the order TBO˙ > AI˙ > CMO˙ in both solvents, whereas the initiation reactions of the monomers exhibit solvent- and initiator-dependent variations.

### 3.2. The propagation reaction

**Fig. 4:**
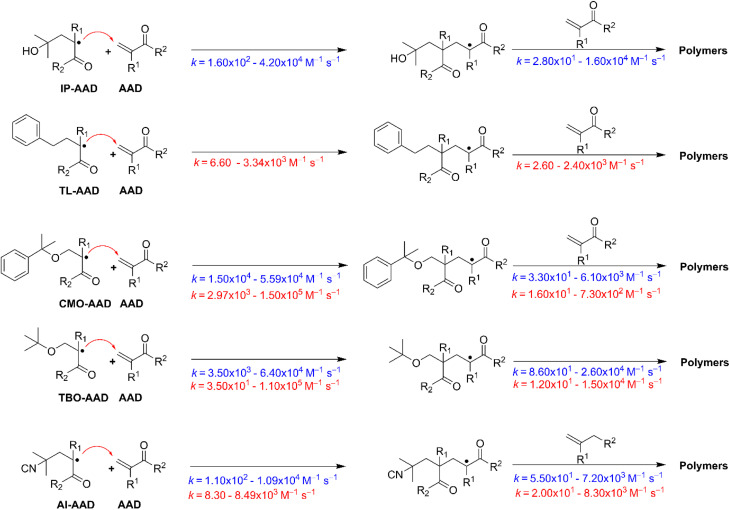
The propagation reactions (green for IP and red for TL solvents) at 298.15 K.

Our previous work predicted that the rate constants of the propagation reactions of AADs in aqueous media exhibit only negligible variation beyond the second propagation step.^36^ Therefore, in the present study, the propagation of AADs was modeled as a two-step reaction, with the corresponding results summarized in Table 2 and illustrated in Fig. 4.

**Table 2:** Calculated Δ*G*^‡^ (kcal mol^−1^), *κ*, *k*_p_ (M^−1^ s^−1^) the propagation reaction

Monomer	Intermediates	IP						TL					
		*n* = 1			*n* = 2			*n* = 1			*n* = 2		
		Δ*G*^‡^	*κ*	*k*_app_	Δ*G*^‡^	*κ*	*k*_app_	Δ*G*^‡^	*κ*	*k*_app_	Δ*G*^‡^	*κ*	*k*_app_
AA	TL–AA/IP–AA	11.4	1.3	2.55 × 10^4^	12.1	1.3	1.20 × 10^4^	13.1	1.3	2.01 × 10^2^	13.2	1.3	1.70 × 10^3^
	CMO–AA	11.9	1.2	1.50 × 10^4^	12.4	1.3	6.10 × 10^3^	11.7	1.3	2.00 × 10^4^	13.9	1.3	5.30 × 10^2^
	TBO–AA	11.6	1.2	2.20 × 10^4^	12.9	1.3	2.70 × 10^3^	12.2	1.3	8.40 × 10^3^	13.0	1.3	2.30 × 10^3^
	AI–AA	12.1	1.2	1.01 × 10^4^	12.2	1.3	7.20 × 10^3^	12.2	1.2	8.49 × 10^3^	12.3	1.3	8.3 × 10^3^
MA	TL–MA/IP–MA	11.3	1.3	4.20 × 10^4^	11.8	1.3	1.60 × 10^4^	12.8	1.3	3.34 × 10^3^	13.0	1.3	2.40 × 10^3^
	CMO–MA	11.2	1.2	4.59 × 10^4^	13.0	1.3	2.20 × 10^3^	10.5	1.2	1.50 × 10^5^	13.7	1.3	7.30 × 10^2^
	TBO–MA	11.1	1.2	5.44 × 10^4^	11.5	1.1	2.60 × 10^4^	10.7	1.2	1.10 × 10^5^	11.9	1.3	1.50 × 10^4^
	AI–MA	12.1	1.3	1.90 × 10^4^	12.5	1.3	5.40 × 10^3^	13.5	1.4	1.20 × 10^3^	13.9	1.3	5.22 × 10^2^
AM	TL–AM/IP–AM	14.2	1.3	3.10 × 10^2^	14.7	1.3	1.50 × 10^2^	16.5	1.3	6.60	16.8	1.3	4.10
	CMO–AM	11.6	1.2	2.40 × 10^4^	15.1	1.3	7.50 × 10^1^	12.6	1.3	4.90 × 10^3^	16.0	1.4	1.60 × 10^1^
	TBO–AM	12.8	1.3	3.50 × 10^3^	14.3	1.3	2.70 × 10^2^	15.5	1.3	3.50 × 10^1^	16.2	1.4	1.20 × 10^1^
	AI–AM	13.9	1.3	5.20 × 10^2^	14.8	1.3	1.20 × 10^2^	12.5	1.3	5.55 × 10^3^	12.6	1.3	4.90 × 10^3^
MAA	TL–MAA/IP–MAA	14.6	1.3	1.60 × 10^2^	15.7	1.4	2.80 × 10^1^	14.4	1.3	2.40 × 10^2^	17.1	1.3	2.60
	CMO–MAA	11.3	1.0	3.23 × 10^4^	15.0	1.3	8.60 × 10^1^	11.3	1.3	4.20 × 10^4^	15.5	1.3	3.60 × 10^1^
	TBO–MAA	12.0	1.0	1.00 × 10^4^	15.6	1.3	3.30 × 10^1^	11.6	1.2	2.50 × 10^4^	15.9	1.3	1.80 × 10^1^
	AI–MAA	14.8	1.3	1.10 × 10^2^	15.3	1.4	5.50 × 10^1^	15.8	1.3	2.11 × 10^1^	15.9	1.4	2.00 × 10^1^
MMA	TL–MMA/IP–MMA	14.6	1.3	1.70 × 10^2^	14.3	1.3	2.60 × 10^2^	13.5	1.2	9.30 × 10^2^	16.2	1.3	1.10 × 10^1^
	CMO–MMA	11.3	1.0	3.00 × 10^4^	13.8	1.3	6.60 × 10^2^	12.8	1.1	2.97 × 10^3^	14.8	1.3	1.20 × 10^2^
	TBO–MMA	10.9	1.0	6.40 × 10^4^	13.5	1.3	9.80 × 10^2^	11.0	1.1	6.05 × 10^4^	15.2	1.3	6.20 × 10^1^
	AI–MMA	14.4	1.3	2.10 × 10^2^	14.5	1.3	1.80 × 10^2^	15.1	1.3	7.60 × 10^1^	15.2	1.3	6.40 × 10^1^

As shown in Table 2, the activation barriers (Δ*G*^‡^) and propagation rate constants (*k*_p_) of the first-step propagation reaction (*n* = 1) in the IP and TL solvents are predicted to have a strong dependence on both the monomer structure and the nature of the initiating radical. For AA and MA, the calculated Δ*G*^‡^ values fall within 11.1–12.1 kcal mol^−1^ in the IP (*e.g.*, 11.6 kcal mol^−1^ for TBO–AA and 12.1 kcal mol^−1^ for AI–AA; 11.2 kcal mol^−1^ for CMO–MA and 12.1 kcal mol^−1^ for AI–MA) and 10.5–13.5 kcal mol^−1^ in TL. These values are slightly lower than those obtained for AM, MAA, and MMA, for which Δ*G*^‡^ generally exceeds 10.9 kcal mol^−1^ and may reach up to 14.8 kcal mol^−1^ in IP (*e.g.*, AI–AM = 13.9 kcal mol^−1^; AI–MAA = 14.8 kcal mol^−1^; AI–MMA = 14.4 kcal mol^−1^) and extends to 16.5 kcal mol^−1^ in TL. This variation in Δ*G*^‡^ leads to pronounced differences in the kinetics. The AA and MA monomers, with relatively low activation barriers, are predicted to have markedly higher *k*_p_ values in the order of 10^4^–10^5^ M^−1^ s^−1^. For instance, in IP solvent, TBO–MA and CMO–MA yield *k*_p_ values of 5.44 × 10^4^ and 4.59 × 10^4^ M^−1^ s^−1^, respectively, whereas in TL solvent, CMO–MA and TBO–MA reach 1.50 × 10^5^ and 1.10 × 10^5^ M^−1^ s^−1^. In contrast, calculations of AM, MAA, and MMA predict slightly lower *k*_p_ values, mostly within 10–10^4^ M^−1^ s^−1^. For example, in IP solvent, AI–AM, AI–MAA, and AI–MMA possess *k*_p_ values of 5.20 × 10^2^, 1.10 × 10^2^, and 2.10 × 10^2^ M^−1^ s^−1^, respectively, while AM–TL shows only 6.6 M^−1^ s^−1^. These findings indicate that simpler monomers (AA, MA), characterized by lower activation barriers, undergo significantly faster first-step propagation compared to bulkier or electronically perturbed monomers (MAA, MMA), in which the steric and electronic effects raise the Δ*G*^‡^ values and consequently reduce the *k*_p_ values.

In terms of radical type, TBO˙ generally predicted to have the highest initiation activity, with *k*_p_ values (*e.g.*, 5.44 × 10^4^ and 1.10 × 10^5^ M^−1^ s^−1^ for MA, *n* = 1 in the IP and TL, respectively), whereas AI˙ exhibits considerably lower efficiency, particularly in monomers with bulky substituents such as MAA and MMA in the IP solvent. By contrast, the reactivity of CMO˙ and IP–C2˙/TL–C7˙ varies substantially depending on the monomer structure. These trends suggest that TBO˙ serves as the most stable and efficient initiator across diverse monomer systems, while AI˙ displays intrinsically low activity, especially in sterically hindered monomers in the IP solvent, highlighting the selective interplay between the nature of the initiating radical and the monomer framework.

Transitioning from the first propagation step (*n* = 1) to the second (*n* = 2) generally results in an increase in Δ*G*^‡^ by 0.1–3.5 kcal mol^−1^ in IP and 0.1–4.3 kcal mol^−1^ in TL, accompanied by a decrease in the corresponding *k*_p_ values, indicating that propagation proceeds more slowly after the initial step. For example, in the propagation reaction of TBO–AA, Δ*G*^‡^ rises from 11.6 to 12.9 kcal mol^−1^ for the IP and 12.2 to 13.0 kcal mol^−1^ for the TL, while *k*_p_ decreases from 2.20 × 10^4^ to 2.70 × 10^3^ M^−1^ s^−1^ and 8.40 × 10^3^ to 2.30 × 10^3^ M^−1^ s^−1^ for the IP and TL, respectively. A similar behavior is observed in TBO–MA, whereas in the TL solvent, the predicted *k*_p_ values of TBO–MA reduce from 1.10 × 10^5^ to 1.50 × 10^4^ M^−1^ s^−1^ (the Δ*G*^‡^ values increase from 10.7 to 11.9 kcal mol^−1^), whereas the rate constant propagation of TBO–MA in the IP changes minor in the second step (*k*_p_ = 4.59–2.60 × 10^4^ M^−1^ s^−1^). This trend is also evident in other monomers such as AM, MAA, and MMA; for instance, in the IP solvent, the Δ*G*^‡^ of the CMO–AM rises from 11.6 to 15.1 kcal mol^−1^, while the *k*_p_ decreases from 2.40 × 10^4^ to 7.50 × 10^1^ M^−1^ s^−1^.

A comparison between the two solvents, IP and TL, clearly suggests that the solvent has a significant influence on the propagation kinetics. In IP, calculations for the monomers AA and MA yield remarkably high rate constants, typically in the range of 10^4^–10^5^ M^−1^ s^−1^. In contrast, although MA retains relatively high reactivity in TL, its *k*_p_ values decrease substantially, reaching a maximum of only 1.50 × 10^5^ M^−1^ s^−1^ for the CMO–MA. Bulkier monomers (MAA, MMA) are more strongly affected by the TL solvent, with predicted *k*_p_ values only within 10–10^3^ M^−1^ s^−1^ (*e.g.*, *k*_p_(*n* = 2)AM–TL = 6.60 M^−1^ s^−1^; *k*_p_(*n* = 2)AI–MAA = 21.1 M^−1^ s^−1^), whereas the corresponding reactions in IP maintain considerably higher *k*_p_ values in the range of 10^2^–10^4^ M^−1^ s^−1^. These results emphasize the critical influence of solvent properties on the reactivity of both simple and sterically hindered monomers during propagation.

In the IP solvent, the propagation rate constants of AADs initiated by TBO˙, CMO˙ and AI˙ were predicted to be within the ranges of 8.60 × 10^1^ to 2.60 × 10^4^, 3.30 × 10^1^ to 6.10 × 10^3^ and 5.50 × 10^1^ to 7.20 × 10^3^ M^−1^ s^−1^, respectively (Fig. 4). By contrast, in the TL solvent, these values decrease to 1.20 × 10^1^ to 1.50 × 10^4^, 1.60 × 10^1^ to 7.30 × 10^2^ and 2.00 × 10^1^ to 8.30 × 10^3^ M^−1^ s^−1^ for TBO˙, CMO˙ and AI˙, respectively. Furthermore, the propagation rate constants of AADs initiated by solvent-derived radicals (IP–C2˙ and TL–C7˙) are found in the range of 2.80 × 10^1^ to 1.60 × 10^4^ and 2.60–2.40 × 10^3^ M^−1^ s^−1^ for the IP and TL, respectively. These data highlight the marked solvent effect on the kinetic profiles of radical-mediated propagation processes. In comparison with the HO˙-initiated AAD reactions in aqueous solution (*k*_p_ = 1.40 × 10^3^ to 3.90 × 10^5^ M^−1^ s^−1^),^36^ the polymerization of AADs initiated by TBO˙, CMO˙, and AI˙ in IP and TL solvents proceeds at relatively lower rates, predicting slower reactivity in these non-aqueous media. This difference suggests that solvent polarity and hydrogen-bonding capability play a critical role in stabilizing transition states and influencing radical propagation efficiency. Consequently, the reduced reactivity observed in IP and TL highlights the importance of solvent effects in determining the kinetics and mechanism of AAD polymerization.

### 3.3. The effect of temperature on the propagation rate constant in the radical polymerization of AAD in the solvents

**Fig. 5:**
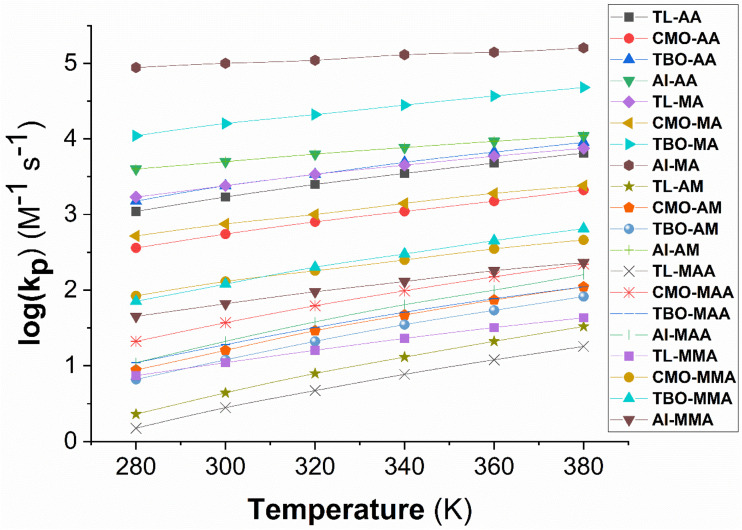
The temperature influence on propagation rate constants (log(*k*_p_)) in TL at 280–380 K.

**Fig. 6:**
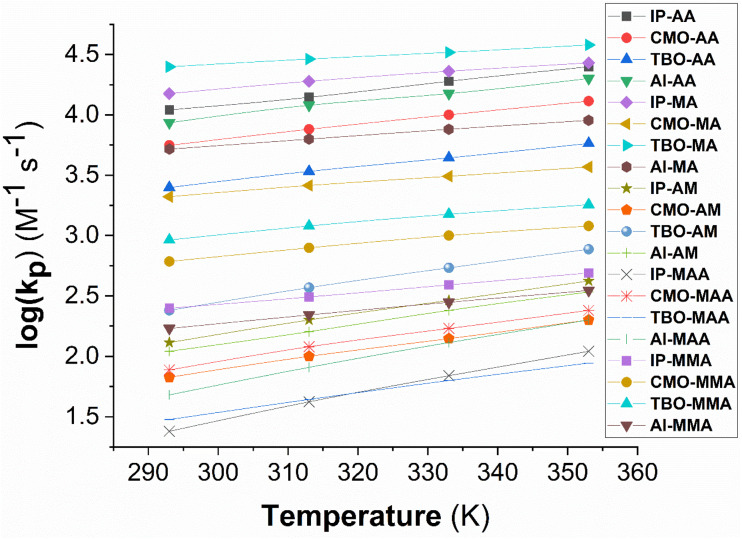
The temperature influence on propagation rate constants (log(*k*_p_)) in IP at 293–353 K.

The dependence of the propagation rate constant (log(*k*_p_)) on temperature was systematically evaluated in the IP and TL over the range of 293–353 K and 280–380 K, respectively, and the results are shown in Fig. 5 and 6.

The results presented in Fig. 5 suggest that the log(*k*_p_) values of all investigated propagation reactions increase almost linearly with temperature in the range of 280–380 K, clearly reflecting the Arrhenius-type dependence of the propagation process. Nevertheless, the pronounced differences among the initiating radicals (*i.e.* TL–C7˙, TBO˙, CMO˙, AI˙) and monomers reveal that polymerization kinetics are not solely governed by temperature but are also substantially influenced by the intrinsic properties of the radical initiator and the chemical structure of the monomers (AA, MA, AM, MAA and MMA).

For the propagation reaction of AA, log(*k*_p_) varies from 2.56 (CMO–AA, 280 K) to 4.04 (AI–AA, 380 K). Throughout the entire temperature range, AI˙ consistently provides the highest values, while CMO˙ gives the lowest, demonstrating the superior initiating efficiency of AI˙ toward AA. In the case of MA, log(*k*_p_) ranges from 2.72 (CMO–MA, 280 K) to 5.20 (AI–MA, 380 K), indicating that MA possesses significantly higher propagation activity than AA, particularly in the AI˙ initiation. By contrast, AM exhibits striking variations: log(*k*_p_) for AI–AM reaches 4.04 at 380 K, whereas TL–AM shows only 0.36 at 280 K, underscoring the critical importance of initiator selection.

Conversely, MAA and MMA were predicted to have considerably lower log(*k*_p_) values. At 380 K, AI–MAA reaches only 2.20, whereas TL–MAA remains at 1.26. The TBO–MMA and CMO–MMA intermediates provide higher log(*k*_p_) values than the AI–MMA (2.81 and 2.66 *vs.* 2.36 at 380 K), suggesting that bulky substituents strongly influence the stability of the transition state, thereby modifying initiating efficiency. A comparison among the monomers reveals that log(*k*_p_) generally follows the order: MA > AA ≈ AM > MMA ≈ MAA. For instance, at 380 K under AI˙ initiation, the log(*k*_p_) values are 5.20 (MA), 4.04 (AA), 4.04 (AM), 2.20 (MAA), and 2.36 (MMA). This trend reflects the combined influence of substituent effects and polarity: acrylates (MA, AA) exhibit higher propagation activity than methacrylates (MAA, MMA) due to their smaller substituents, which reduce steric hindrance. The calculated data predict that the propagation rate constant of MA polymerization in toluene at 320 K ranges from 1.00 × 10^3^ to 1.10 × 10^5^ M^−1^ s^−1^ (Fig. 5), which shows strong agreement with the reported experimental value (*k*_p_(exp) = 2.82 × 10^4^ M^−1^ s^−1^).^25^ The results further indicate that the radical polymerization of MA and MMA follows the Arrhenius kinetic model, with activation energies of 3.6 and 3.4 kcal mol^−1^ in the temperature range of 280–380 K, respectively. These values are in good agreement with experimental data (*E*_a_(MA) = 4.1 (ref. 28) and 4.4 kcal mol^−1^,^27^ and *E*_a_(MMA) = 2.8–5.3 kcal mol^−1^ at 303–333 K (ref. 24)), thereby confirming the reliability of the computational approach employed in this study. Thus, in the TL solvent, the data demonstrate that AI˙ is the most effective initiator for the majority of monomers, except for MMA, where TBO˙ and CMO˙ show higher performance. At the same time, monomer structure plays a decisive role in determining propagation reactivity.

The results presented in Fig. 6 demonstrate that log(*k*_p_) values for all systems increase as the temperature rises from 293 K to 353 K, confirming the acceleration of the chain propagation stage in free-radical polymerization under thermal energy. The near-linear relationship between log(*k*_p_) and temperature indicates that the reaction kinetics are primarily governed by thermodynamic factors rather than being restricted by diffusion processes.

Considering the individual monomers, calculations for AA, MA, and AM generally yield higher log(*k*_p_) values than MAA and MMA. At 293 K, the log(*k*_p_) of the propagation of IP–AA reaches 4.04, whereas that of IP–MAA is only 1.38, a difference of 2.66 log units. Similarly, in the propagation of IP–MA, log(*k*_p_) is 4.18, more than three times higher than in the IP–MMA (2.40). Whereas that of the TBO–MA achieves 4.40, substantially higher than TBO–MMA (2.61) and TBO–MAA (1.57). This clearly reflects the steric hindrance imposed by the –CH_3_ substituent in methacrylic monomers, which reduces the radical addition efficiency and thereby suppresses chain propagation. This trend remains consistent throughout the investigated temperature range. With increasing temperature, all systems display growth in log(*k*_p_), though to varying extents. For instance, in the IP–AA reaction, log(*k*_p_) increases from 4.04 (293 K) to 4.40 (353 K), yielding Δlog(*k*_p_) = 0.36. In contrast, for IP–MMA, log(*k*_p_) rises only from 2.40 to 2.69 (Δlog(*k*_p_) = 0.29). The smaller increment in methacrylic systems suggests that although temperature enhances kinetics, steric hindrance remains a significant limiting factor. Studies in the AM monomer indicate that although its log(*k*_p_) values are lower than those of AA and MA, they are still noticeably higher than those of the methacrylic systems. For example, at 353 K, log(*k*_p_) of IP–AM (2.62) surpasses those of IP–MAA (2.04) and IP–MMA (2.69). This may imply that hydrogen-bonding interactions between isopropanol and the –CONH_2_ group of AM provide an additional stabilizing effect, enhancing radical orientation during the propagation step.

The influence of the radical species is also clearly evident. Calculations for TBO˙ consistently yields the highest log(*k*_p_) values. For example, with MA at 353 K, TBO–MA reaches 4.58, while CMO–MA is 3.57 and AI–MA is 3.95. This indicates that TBO˙ possesses superior electronic stabilization and a more favorable transition state, facilitating efficient chain propagation. In contrast, CMO˙ generally produces lower log(*k*_p_) values. With AA at 293 K, log(*k*_p_) of CMO–AA is only 3.14, compared with 4.22 for TBO–AA and 4.04 for IP–AA. AI˙ exhibits intermediate values, ranging from 2.04 (AI–AM, 293 K) to 4.20 (AI–AA, 353 K), reflecting its moderate stability in propagation reactions. Comparisons within the same monomer show a consistent trend: TBO˙ dominates, AI˙ is intermediate, and CMO˙ is the lowest. For example, with AM at 353 K, log(*k*_p_) of TBO–AM is 2.89, compared with 2.53 for AI–AM and 1.98 for CMO–AM. These results highlight the decisive role of radical identity in controlling propagation kinetics.

The comparative analysis between isopropanol and toluene demonstrates that log(*k*_p_) consistently increases with rising temperature in both solvents, in agreement with Arrhenius kinetics. However, the predicted magnitude of this increase is more pronounced in toluene, where steric and polarity effects of the solvent impose fewer restrictions on radical propagation. In contrast, hydrogen-bonding interactions in isopropanol partially moderate the temperature effect. These findings not only validate the Arrhenius-based kinetic model but also highlight that the judicious choice of initiator and rational monomer design are critical strategies for controlling free-radical polymerization kinetics.

## 4. Conclusion

Quantum chemical calculations were conducted to study the radical polymerization of AADs, including AA, MA, AM, MAA, and MMA, initiated by CMO˙, TBO˙, and AI˙ in the IP and TL solvents. The results predict that initiation reactions proceed more rapidly in IP than in TL for all examined AADs and radicals (IP–C2˙, TL–C7˙, TBO˙, CMO˙, and AI˙). Among the initiators, the relative efficiency follows the order TBO˙ > AI˙ > CMO˙ in both solvents, although the initiation of individual monomers exhibits solvent- and initiator-dependent variations. In IP at 298 K, the propagation rate constants of AADs initiated by TBO˙, CMO˙, and AI˙ were predicted to be in the ranges of 8.60 × 10^1^ to 2.60 × 10^4^, 3.30 × 10^1^ to 6.10 × 10^3^ and 5.50 × 10^1^ to 5.40 × 10^3^ M^−1^ s^−1^, respectively, while in TL, these values were significantly lower, ranging from 1.20 × 10^1^ to 1.50 × 10^4^, 1.60 × 10^1^ to 7.30 × 10^2^ and 2.00 × 10^1^ to 4.90 × 10^3^ M^−1^ s^−1^, respectively. Additionally, solvent-derived radicals also contributed to the propagation reactions, with *k*_p_ values of 2.80 × 10^1^ to 2.60 × 10^4^ M^−1^ s^−1^ in IP and 2.60–1.50 × 10^4^ M^−1^ s^−1^ in TL. The investigation of temperature effects on the polymerization rate constant reveals that temperature markedly enhances log(*k*_p_) in both solvents, consistent with the Arrhenius kinetic model. The calculated *k*_p_ of MA polymerization in toluene (1.00 × 10^3^ to 1.10 × 10^5^ M^−1^ s^−1^ at 320 K) and the activation energies for MA and MMA (3.6 and 3.4 kcal mol^−1^, respectively) show excellent agreement with experimental data, confirming the reliability of the computational method. Nevertheless, the acceleration is more pronounced in toluene than in isopropanol due to the absence of hydrogen-bonding constraints, emphasizing the defining role of solvent nature in governing propagation kinetics. These findings underscore the pronounced influence of solvent environments on both the initiation and propagation stages of radical polymerization, thereby providing valuable mechanistic insights for optimizing reaction conditions in non-aqueous media.

## Conflicts of interest

There are no conflicts to declare.

## Supplementary Material

RA-016-D5RA09849A-s001

## Data Availability

The data supporting this article have been included as part of the supplementary information (SI). Supplementary information is available. See DOI: https://doi.org/10.1039/d5ra09849a.
