# Supplementary material for: Computational study of the kinetics and mechanism of radical polymerization of acrylic acid and derivatives in organic solvents
Source: RSC Adv. 2026 Feb 10;16(9):8289–99. doi: 10.1039/d5ra09849a (PMC12890304; doi:10.1039/d5ra09849a)
Supplement: RA-016-D5RA09849A-s001 [file RA-016-D5RA09849A-s001.pdf]

Supplementary Information (SI) for RSC Advances.
This journal is © The Royal Society of Chemistry 2026

Supporting Information (SI)

Computational study of the kinetics and mechanism of radical polymerization of acrylic acid and derivatives in organic solvents

Mai Van Bay,[1] Pham Thi Thuy Linh,[1] Truong Le Bich Tram,[2] Nguyen Thi Hoa[3], Adam Mechler[4] and Quan V. Vo[3*]

[1]The University of Danang - University of Sciences and Education, Danang 550000, Vietnam.
[2]Department of Science and International Cooperation, The University of Danang, Danang 550000, Vietnam
[3]The University of Danang - University of Technology and Education, Danang 550000, Vietnam.
[4]Department of Biochemistry and Chemistry, La Trobe University, Victoria 3086, Australia.
**Corresponding authors: vvquan@ute.udn.vn;*

Table of Contents

Table S1. The calculated values of the ΔG^o for the TBO•/CMO•/AI• + TL/IP reactions in terms of kcal/mol according to the mechanisms. S2

Table S2: The Cartesian coordinates, energies of TS of the reaction between FPHs with HO• in the studied media (IP: isopropanol; TL: Toluene) S3

Table S1. The calculated values of the ΔG° for the TBO•/CMO•/AI• + TL/IP reactions in terms of kcal/mol according to the mechanisms.

Solvents	Mechanism	Positions	**TBO**	**CMO**	**AI**
		C1-H	-5.2	-5.4	13.9
IP	FHT	C2-H	-13.8	-14.0	5.3
	FHT	O2-H	-0.8	-1.0	18.3
		C7-H	-13.0	-13.4	4.1
TL		C2	10.9	12.2	18.7
	RAF	C3	9.4	10.5	16.0
		C4	8.5	9.4	15.2

Table S2: The Cartesian coordinates, energies of TS of the reaction between FPHs with HO• in the studied media (IP: isopropanol; TL: Toluene)

Cartesian Coordinates | **Energy**

AA-C2-AI-TS-IP

Atom	X	Y	Z		
O	1.818167	-0.610211	-0.947152	Electronic Energy (EE)	-477.8369298
O	3.807112	-1.386013	-0.276362	Zero-point Energy Correction	0.158242
C	3.692657	0.704014	-1.441521	Thermal Correction to Energy	0.169467
C	3.147803	-0.533753	-0.824886	Thermal Correction to Enthalpy	0.170412
C	4.955677	0.652497	-1.972192	Thermal Correction to Free Energy	0.121052
H	2.958603	1.393499	-1.845527		
H	5.3234	1.44683	-2.609275		
H	5.626841	-0.162382	-1.726577		
H	1.507276	-1.429344	-0.52784		
C	3.756434	1.906788	0.378535		
C	2.414459	1.719767	1.032571		
H	2.284397	0.714525	1.436412		
H	2.317377	2.433186	1.860105		
H	1.617507	1.929637	0.315614		
C	4.042002	3.258338	-0.218958		
H	4.12142	4.002862	0.581429		
H	4.97559	3.261817	-0.783483		
H	3.220414	3.554271	-0.874931		
C	4.862503	1.294832	1.041001		
N	5.773179	0.778902	1.530033		

AA-C2-AI-TS-TL

Atom	X	Y	Z		
O	1.766361	-0.5152	-1.005769	Electronic Energy (EE)	-477.8258569
O	3.694795	-1.402162	-0.298447	Zero-point Energy Correction	0.158434
C	3.721394	0.700073	-1.450241	Thermal Correction to Energy	0.169843
C	3.103574	-0.51385	-0.85131	Thermal Correction to Enthalpy	0.170787
C	5.002945	0.593191	-1.924431	Thermal Correction to Free Energy	0.120482
H	3.031795	1.409819	-1.894448		
H	5.433097	1.361894	-2.553427		
H	5.628229	-0.24035	-1.627866		
H	1.424996	-1.328096	-0.604159		
C	3.767805	1.907734	0.372772		
C	2.452153	1.671834	1.065063		
H	2.374309	0.662374	1.47108		
H	2.350826	2.377015	1.899152		
H	1.62373	1.847332	0.375543		
C	3.97919	3.271787	-0.230307		

H	4.080155	4.018393	0.56561
H	4.882678	3.307127	-0.840885
H	3.118489	3.550653	-0.842275
C	4.918387	1.353412	1.015288
N	5.86791	0.89708	1.486053

AA-C2-CM-TS-IP

Atom	X	Y	Z	Electronic Energy (EE)	-691.8137535
O	-1.513082	1.366544	-0.274043	Zero-point Energy Correction	0.245961
O	-0.531066	1.620024	-2.270247	Thermal Correction to Energy	0.260716
C	0.254075	-0.079405	-0.780406	Thermal Correction to Enthalpy	0.26166
C	-0.616339	1.048645	-1.210093	Thermal Correction to Free Energy	0.203114
C	1.036914	-0.718976	-1.694563		
H	-0.00363	-0.552407	0.158254		
H	1.573383	-1.621377	-1.43031		
H	1.170553	-0.310134	-2.689567		
H	-2.042861	2.118841	-0.584546		
O	1.53659	1.290166	-0.061784		
C	2.092403	0.958808	1.197053		
C	3.171884	2.04404	1.369221		
C	1.028094	1.005675	2.29089		
C	2.7652	-0.42025	1.172439		
C	3.221624	2.88267	2.482482		
C	4.144664	2.191227	0.373141		
C	4.232588	3.835989	2.60673		
C	5.149209	3.143232	0.494843		
C	5.197723	3.970503	1.615913		
H	0.274932	0.237503	2.104526		
H	1.462208	0.809496	3.274066		
H	0.531371	1.978391	2.309092		
H	2.012903	-1.208324	1.084027		
H	3.31736	-0.578078	2.102183		
H	3.459124	-0.497271	0.333282		
H	2.480351	2.801985	3.267685		
H	4.108913	1.55906	-0.507024		
H	4.258118	4.475635	3.482094		
H	5.893825	3.242622	-0.287273		
H	5.980584	4.714406	1.712532		

AA-C2-CM-TS-TL

Atom	X	Y	Z	Electronic Energy (EE)	-691.8046706
O	-1.523144	1.341526	-0.349786	Zero-point Energy Correction	0.246158
O	-0.528377	1.528243	-2.346356	Thermal Correction to Energy	0.260994
C	0.27668	-0.093348	-0.781872	Thermal Correction to Enthalpy	0.261938

C	-0.605953	1.005094	-1.268372	Thermal Correction to Free Energy	0.20308
C	1.087695	-0.739437	-1.664876		
H	0.001828	-0.547472	0.161036		
H	1.639215	-1.624319	-1.375303		
H	1.230862	-0.339234	-2.661467		
H	-2.042434	2.076424	-0.706977		
O	1.518926	1.28681	-0.056535		
C	2.066379	0.985398	1.206755		
C	3.156208	2.060966	1.370981		
C	0.997176	1.058347	2.296272		
C	2.742427	-0.394862	1.214762		
C	3.299465	2.808648	2.538521		
C	4.053922	2.275321	0.320407		
C	4.330005	3.73948	2.660756		
C	5.078149	3.205307	0.440791		
C	5.22195	3.940667	1.615167		
H	0.212504	0.326799	2.09432		
H	1.411127	0.831398	3.281367		
H	0.537772	2.048362	2.318651		
H	1.996014	-1.190268	1.138059		
H	3.293492	-0.533594	2.1477		
H	3.440591	-0.480156	0.380065		
H	2.612996	2.675622	3.365022		
H	3.931277	1.71798	-0.600621		
H	4.428537	4.309905	3.577364		
H	5.762561	3.361488	-0.385276		
H	6.019768	4.668147	1.71004		

AA-C2-IP-TS-IP

Atom	X	Y	Z		
O	-1.205607	-1.264825	-0.583705	Electronic Energy (EE)	-460.8245815
O	-1.032652	0.89421	-0.02874	Zero-point Energy Correction	0.163671
C	0.938407	-0.465165	-0.083895	Thermal Correction to Energy	0.174325
C	-0.501131	-0.18091	-0.216906	Thermal Correction to Enthalpy	0.17527
C	1.836176	0.552556	-0.257212	Thermal Correction to Free Energy	0.12751
H	1.232641	-1.495273	-0.254307		
H	2.897635	0.349953	-0.328984		
H	1.510933	1.586415	-0.270739		
H	-2.144312	-1.024094	-0.635901		
O	2.104954	-1.498886	2.261094		
C	0.863366	-0.939831	2.124479		
C	-0.289545	-1.871942	2.31059		
C	0.809431	0.415727	2.737046		
H	-1.238544	-1.36136	2.140934		
H	-0.223718	-2.732878	1.638946		

H	-0.28703	-2.246676	3.343763
H	-0.109531	0.933347	2.458116
H	1.668956	1.011927	2.42037
H	0.838347	0.327877	3.83106
H	2.082633	-2.428044	1.996642

AA-C2-TB-TS-IP

Atom	X	Y	Z		
O	-1.527669	-0.293926	-0.460545	Electronic Energy (EE)	-500.1048732
O	-0.378701	1.541866	0.107237	Zero-point Energy Correction	0.192157
C	0.750038	-0.564137	-0.001703	Thermal Correction to Energy	0.203976
C	-0.415808	0.35389	-0.105609	Thermal Correction to Enthalpy	0.20492
C	2.011267	-0.050667	0.056171	Thermal Correction to Free Energy	0.154164
H	0.581456	-1.59078	-0.300245		
H	2.877152	-0.697714	-0.00157		
H	2.167812	1.011221	0.207793		
H	-2.266037	0.336267	-0.491165		
O	0.317769	-0.780862	1.943698		
C	0.417634	-2.115449	2.394037		
C	-0.654153	-3.003858	1.762911		
C	0.163845	-1.988955	3.907148		
C	1.819062	-2.678874	2.154669		
H	-0.493262	-3.123904	0.688607		
H	-1.644334	-2.568053	1.91931		
H	-0.637623	-3.998757	2.215731		
H	-0.819206	-1.550994	4.093775		
H	0.197193	-2.982304	4.361851		
H	0.927351	-1.360693	4.370364		
H	2.010447	-2.803828	1.085578		
H	1.917855	-3.656302	2.633764		
H	2.572936	-2.005757	2.570558		

AA-C2-TB-TS-TL

Atom	X	Y	Z		
O	-1.541872	-0.286097	-0.433951	Electronic Energy (EE)	-500.0957396
O	-0.370903	1.556782	0.056703	Zero-point Energy Correction	0.19238
C	0.74239	-0.56107	0.003774	Thermal Correction to Energy	0.204271
C	-0.416687	0.368105	-0.108541	Thermal Correction to Enthalpy	0.205215
C	2.00406	-0.051335	0.060411	Thermal Correction to Free Energy	0.154259
H	0.565141	-1.585944	-0.295394		
H	2.871659	-0.696012	0.009154		
H	2.153429	1.010271	0.216485		
H	-2.260209	0.362147	-0.464357		
O	0.325316	-0.791666	1.936796		

C	0.421527	-2.119635	2.388387
C	-0.647104	-3.015238	1.759109
C	0.161948	-1.985869	3.900282
C	1.824966	-2.687069	2.16075
H	-0.485421	-3.142317	0.685978
H	-1.636498	-2.57644	1.904791
H	-0.63417	-4.008353	2.216053
H	-0.819974	-1.543427	4.0783
H	0.191769	-2.97472	4.364923
H	0.922022	-1.351985	4.359844
H	2.02243	-2.823417	1.094001
H	1.925518	-3.660754	2.646989
H	2.575685	-2.008526	2.571879

AA-C2-TL-TS-TL

Atom	X	Y	Z		
				Electronic Energy (EE)	-537.9938318
O	-0.616199	-2.017932	-0.226495	Zero-point Energy Correction	0.184716
O	-1.221041	0.118415	0.038873	Thermal Correction to Energy	0.195886
C	1.097325	-0.475149	0.169216	Thermal Correction to Enthalpy	0.19683
C	-0.356547	-0.719883	-0.009187	Thermal Correction to Free Energy	0.146237
C	1.601268	0.751471	-0.170435		
H	1.727423	-1.356924	0.143185		
H	2.668912	0.921391	-0.223711		
H	0.93668	1.592235	-0.329184		
H	-1.578424	-2.126155	-0.251897		
C	0.058945	-4.078621	3.177624		
C	0.805138	-2.954024	2.863016		
C	0.190193	-1.693533	2.73508		
C	-1.202423	-1.607596	2.931882		
C	-1.943801	-2.735907	3.249745		
C	-1.318843	-3.976644	3.371459		
H	0.54909	-5.040611	3.274631		
H	1.876698	-3.036718	2.711253		
H	-1.690355	-0.643538	2.832157		
H	-3.01309	-2.650241	3.406898		
H	-1.899823	-4.857135	3.619393		
C	0.951094	-0.533699	2.34437		
H	0.483308	0.439054	2.445336		
H	2.025798	-0.556379	2.48325		

AA-C3-AI-TS-IP

Atom	X	Y	Z		
				Electronic Energy (EE)	-477.844429
O	1.995005	-1.485261	-1.360412	Zero-point Energy Correction	0.158467
O	4.130854	-1.412193	-0.690832	Thermal Correction to Energy	0.16988

C	3.085304	0.571559	-1.507681	Thermal Correction to Enthalpy	0.170824
C	3.155275	-0.844281	-1.139601	Thermal Correction to Free Energy	0.119905
C	4.15809	1.387402	-1.318845		
H	2.143006	0.939493	-1.895018		
H	4.15847	2.382516	-1.749616		
H	5.120021	0.954203	-1.061763		
H	2.101285	-2.41779	-1.114479		
C	3.911728	2.26035	0.71797		
C	3.874392	1.054117	1.607912		
H	4.777227	0.448819	1.516727		
H	3.779822	1.378895	2.651475		
H	3.00009	0.445194	1.367767		
C	2.655477	3.066918	0.567793		
H	2.430976	3.568906	1.516737		
H	2.748165	3.827721	-0.208528		
H	1.820245	2.40483	0.33141		
C	5.134596	2.986468	0.690953		
N	6.136205	3.560585	0.624779		

AA-C3-AI-TS-TL

Atom	X	Y	Z	Electronic Energy (EE)	-477.8352748
O	2.011509	-1.504938	-1.365594	Zero-point Energy Correction	0.159032
O	4.129367	-1.391435	-0.649748	Thermal Correction to Energy	0.170426
C	3.077364	0.567624	-1.513276	Thermal Correction to Enthalpy	0.17137
C	3.162607	-0.845402	-1.126483	Thermal Correction to Free Energy	0.120653
C	4.144109	1.39054	-1.322973		
H	2.136588	0.92068	-1.917164		
H	4.149078	2.382007	-1.761152		
H	5.104391	0.95671	-1.06282		
H	2.143465	-2.427015	-1.102322		
C	3.913868	2.263745	0.715106		
C	3.863239	1.049416	1.594944		
H	4.744569	0.418617	1.476239		
H	3.798278	1.360223	2.644771		
H	2.972052	0.461901	1.36463		
C	2.661932	3.081416	0.576724		
H	2.433484	3.569409	1.531828		
H	2.760159	3.857773	-0.183238		
H	1.821948	2.431373	0.323973		
C	5.142522	2.982991	0.694628		
N	6.145706	3.552813	0.628675		

AA-C3-CM-TS-IP

Atom	X	Y	Z	Electronic Energy (EE)	-691.8172094

O	-1.533624	0.516743	-0.153379	Zero-point Energy Correction	0.246528
O	-0.837512	1.789398	-1.859146	Thermal Correction to Energy	0.261182
C	0.50081	-0.051421	-1.141796	Thermal Correction to Enthalpy	0.262126
C	-0.662085	0.848843	-1.115778	Thermal Correction to Free Energy	0.203308
C	1.505513	0.157958	-2.034392		
H	0.547075	-0.830679	-0.390954		
H	2.319185	-0.551271	-2.115654		
H	1.362232	0.847393	-2.856872		
H	-2.277769	1.139933	-0.173586		
O	2.569961	1.617428	-1.11793		
C	3.08385	1.38346	0.174944		
C	4.238557	2.407983	0.212634		
C	2.026511	1.652292	1.242653		
C	3.646854	-0.033277	0.32628		
C	4.311562	3.420502	1.169155		
C	5.247942	2.318644	-0.753556		
C	5.386439	4.308964	1.176004		
C	6.315412	3.207971	-0.749557		
C	6.390269	4.20643	0.219847		
H	1.243403	0.894934	1.168654		
H	2.454093	1.595813	2.246506		
H	1.572041	2.635485	1.101997		
H	2.83477	-0.764679	0.324945		
H	4.18085	-0.118229	1.275818		
H	4.337231	-0.271612	-0.484761		
H	3.541495	3.524542	1.923047		
H	5.194621	1.550603	-1.516787		
H	5.433096	5.084025	1.933022		
H	7.088971	3.123151	-1.504699		
H	7.223366	4.900292	0.226001		

AA-C3-CM-TS-TL

Atom	X	Y	Z	Electronic Energy (EE)	-691.8084789
O	-1.538842	0.527303	-0.166198	Zero-point Energy Correction	0.246716
O	-0.911955	1.676056	-1.98267	Thermal Correction to Energy	0.261455
C	0.497998	-0.057987	-1.144231	Thermal Correction to Enthalpy	0.2624
C	-0.689645	0.811603	-1.173438	Thermal Correction to Free Energy	0.203179
C	1.521099	0.161966	-2.013041		
H	0.540777	-0.821167	-0.377141		
H	2.344721	-0.536582	-2.079104		
H	1.377879	0.845694	-2.839611		
H	-2.294017	1.128269	-0.241562		
O	2.567705	1.610198	-1.103517		
C	3.081279	1.394779	0.186736		
C	4.240045	2.414382	0.21814		

C	2.023849	1.676953	1.252477
C	3.648172	-0.020064	0.358396
C	4.355843	3.390313	1.206451
C	5.213135	2.352473	-0.784923
C	5.437344	4.269482	1.207139
C	6.286925	3.232706	-0.787575
C	6.404944	4.194112	0.213305
H	1.225663	0.936892	1.170412
H	2.441112	1.607194	2.259877
H	1.587586	2.667299	1.1091
H	2.840916	-0.757164	0.359642
H	4.179116	-0.095822	1.310021
H	4.346186	-0.261661	-0.445236
H	3.610609	3.475905	1.986752
H	5.117324	1.616765	-1.574699
H	5.516441	5.017682	1.987575
H	7.030497	3.171967	-1.57397
H	7.242415	4.882075	0.213554

AA-C3-IP-TS-IP

Atom	X	Y	Z	Electronic Energy (EE)	-460.831346
C	1.572842	-0.330813	-0.784051	Zero-point Energy Correction	0.163287
C	2.744148	0.338614	-0.682516	Thermal Correction to Energy	0.174232
H	1.510058	-1.325663	-1.207618	Thermal Correction to Enthalpy	0.175176
H	3.637323	-0.050717	-1.157107	Thermal Correction to Free Energy	0.126177
H	2.768264	1.369513	-0.344191		
O	3.901375	-1.673002	1.169929		
C	3.783919	-0.325893	1.370949		
C	2.744958	0.101499	2.348497		
C	5.088369	0.38121	1.285652		
H	2.631557	1.186809	2.346583		
H	1.777512	-0.355678	2.112556		
H	3.024562	-0.214085	3.363995		
H	4.935734	1.460907	1.237424		
H	5.648623	0.059722	0.404169		
H	5.703262	0.166637	2.171774		
H	3.028437	-2.085165	1.230069		
C	0.344415	0.235628	-0.270136		
O	0.209603	1.370546	0.207104		
O	-0.72729	-0.58161	-0.3838		
H	-1.533638	-0.118385	-0.115976		

AA-C3-TB-TS-IP

Atom	X	Y	Z	Electronic Energy (EE)	-500.1076178

O	-0.426995	0.999935	-0.760267	Zero-point Energy Correction	0.192393
O	1.696174	0.603499	-1.349792	Thermal Correction to Energy	0.20421
C	0.709475	-0.650944	0.427783	Thermal Correction to Enthalpy	0.205154
C	0.745116	0.361193	-0.637812	Thermal Correction to Free Energy	0.154016
C	1.823752	-1.359824	0.76353		
H	-0.234891	-0.802554	0.936372		
H	1.731359	-2.242179	1.381063		
H	2.730385	-1.25733	0.17826		
H	-0.363929	1.64107	-1.486537		
O	2.453445	-0.437947	2.44041		
C	2.97712	0.865052	2.381291		
C	3.694198	0.976996	3.745957		
C	1.874525	1.920425	2.302945		
C	3.990153	1.0215	1.250547		
H	4.47648	0.220443	3.830612		
H	2.982602	0.8521	4.564533		
H	4.148352	1.968233	3.819208		
H	1.120943	1.737157	3.072718		
H	2.293108	2.918962	2.453544		
H	1.386821	1.906186	1.32541		
H	4.75823	0.246716	1.319704		
H	4.474824	1.999138	1.313366		
H	3.497693	0.947392	0.278488		

AA-C3-TB-TS-TL

Atom	X	Y	Z	Electronic Energy (EE)	-500.1005796
O	-0.454207	0.965393	-0.789551	Zero-point Energy Correction	0.192942
O	1.699491	0.648458	-1.302404	Thermal Correction to Energy	0.204757
C	0.700026	-0.651842	0.433916	Thermal Correction to Enthalpy	0.205701
C	0.739087	0.364823	-0.626605	Thermal Correction to Free Energy	0.154591
C	1.821679	-1.34771	0.771856		
H	-0.24421	-0.812255	0.938832		
H	1.7435	-2.23176	1.387694		
H	2.722419	-1.233189	0.180257		
H	-0.364213	1.605965	-1.509858		
O	2.448421	-0.43131	2.434004		
C	2.978595	0.863742	2.377412		
C	3.692977	0.968879	3.744083		
C	1.880656	1.926156	2.300332		
C	3.996306	1.021304	1.249014		
H	4.469309	0.206798	3.827808		
H	2.978998	0.841643	4.559472		
H	4.153335	1.956539	3.825289		
H	1.126389	1.742547	3.068426		
H	2.300344	2.924364	2.44992		

H	1.392348	1.913018	1.323566
H	4.757402	0.239827	1.316377
H	4.490925	1.993523	1.318223
H	3.509363	0.960306	0.273271

AA-C3-TL-TS-TL

Atom	X	Y	Z	Electronic Energy (EE)	-537.9998279
O	-1.465161	-0.671239	-0.480231	Zero-point Energy Correction	0.185174
O	-0.56795	1.293018	0.10748	Thermal Correction to Energy	0.196453
C	0.845019	-0.610744	-0.130999	Thermal Correction to Enthalpy	0.197397
C	-0.421939	0.118764	-0.148114	Thermal Correction to Free Energy	0.145249
C	1.999241	0.05148	0.147595		
H	0.811583	-1.682597	-0.279897		
H	2.954865	-0.451311	0.057245		
H	2.008712	1.135653	0.130696		
H	-2.259757	-0.119403	-0.468795		
C	4.605893	2.994585	2.697763		
C	3.417387	2.294846	2.563255		
C	3.407511	0.884816	2.561581		
C	4.638945	0.209253	2.684971		
C	5.824136	0.914754	2.819481		
C	5.81457	2.309767	2.826888		
H	4.59502	4.078498	2.702719		
H	2.477434	2.827762	2.460875		
H	4.647692	-0.876102	2.681087		
H	6.761417	0.379769	2.920817		
H	6.742482	2.859239	2.932201		
C	2.182995	0.16058	2.380221		
H	2.173576	-0.90825	2.555149		
H	1.240291	0.681064	2.497641		

AA2-C2-IP-TS-IP

Atom	X	Y	Z	Electronic Energy (EE)	-728.0055732
O	-1.259197	-1.076835	0.494761	Zero-point Energy Correction	0.235556
O	-1.070422	1.088272	1.004613	Thermal Correction to Energy	0.251615
C	0.843079	-0.35163	1.322537	Thermal Correction to Enthalpy	0.252559
C	-0.579221	-0.013034	0.933348	Thermal Correction to Free Energy	0.190146
C	1.783708	0.691165	0.817613		
H	1.085933	-1.325505	0.884857		
H	2.844401	0.506117	0.931229		
H	1.460122	1.724303	0.805042		
H	-2.169481	-0.808455	0.285923		
O	2.265637	-1.058341	3.144047		
C	0.954979	-0.561449	2.881531		

C	-0.084964	-1.568115	3.360755
C	0.84421	0.747281	3.646588
H	-1.098673	-1.170789	3.264988
H	-0.021397	-2.498568	2.788942
H	0.095218	-1.791202	4.414915
H	-0.106543	1.241573	3.439593
H	1.65835	1.423143	3.376236
H	0.904821	0.542629	4.718052
H	2.336826	-1.950311	2.781231
O	4.69836	1.916431	-2.783765
O	4.549709	-0.068168	-1.758453
C	2.581584	1.245853	-2.066201
C	4.007299	0.939365	-2.169984
C	1.734355	0.356735	-1.499504
H	2.253284	2.219676	-2.407931
H	0.666091	0.54423	-1.506578
H	2.066454	-0.652569	-1.280121
H	5.633452	1.661402	-2.821072

AA2-C3-AI-TS-IP

Atom	X	Y	Z
O	-1.37413	-1.573124	-1.94091
O	-1.126595	0.451722	-1.005314
C	0.618743	-1.150124	-0.803786
C	-0.690135	-0.660806	-1.228824
C	1.407018	-0.364766	0.191483
H	0.785008	-2.216591	-0.894541
H	2.47556	-0.515049	0.013628
H	1.18655	0.699177	0.077176
H	-2.209633	-1.176456	-2.235639
C	1.112667	-0.784728	1.664398
C	1.522427	-2.24089	1.928485
C	-0.356621	-0.557296	2.04701
H	0.878041	-2.908889	1.353645
H	2.56123	-2.41493	1.639907
H	1.405505	-2.481452	2.986963
H	-0.990993	-1.226862	1.461108
H	-0.659089	0.473824	1.855778
H	-0.509473	-0.782265	3.104443
O	-0.52292	-1.446649	-5.375267
O	-0.442299	0.45761	-4.197587
C	1.105312	-1.287646	-3.710644
C	-0.009433	-0.653882	-4.422757
C	1.7209	-0.639409	-2.687152
H	1.364398	-2.302187	-3.988008

Electronic Energy (EE)	-745.0332385
Zero-point Energy Correction	0.231487
Thermal Correction to Energy	0.247888
Thermal Correction to Enthalpy	0.248832
Thermal Correction to Free Energy	0.185917

H	2.616213	-1.063468	-2.247883
H	1.532281	0.417475	-2.522404
H	-1.250248	-0.979026	-5.815648
C	1.946498	0.084633	2.51074
N	2.600415	0.763861	3.16981

AA2-C3-AI-TS-TL

Atom	X	Y	Z		
O	-1.415141	-1.444317	-1.905516	Electronic Energy (EE)	-745.0163524
O	-1.099025	0.541713	-0.908411	Zero-point Energy Correction	0.232034
C	0.615937	-1.096877	-0.805313	Thermal Correction to Energy	0.248512
C	-0.694441	-0.563709	-1.182486	Thermal Correction to Enthalpy	0.249456
C	1.427252	-0.365023	0.212993	Thermal Correction to Free Energy	0.186197
H	0.759761	-2.161977	-0.938676		
H	2.492726	-0.534156	0.032029		
H	1.231568	0.706547	0.128194		
H	-2.21449	-0.98452	-2.202062		
C	1.124619	-0.813355	1.676815		
C	1.480603	-2.291244	1.896408		
C	-0.336478	-0.547976	2.067111		
H	0.815458	-2.92143	1.302607		
H	2.513886	-2.496633	1.60859		
H	1.354174	-2.560956	2.946433		
H	-0.994413	-1.194745	1.482044		
H	-0.614261	0.489837	1.87893		
H	-0.491659	-0.772587	3.124003		
O	-0.403506	-1.643228	-5.421568		
O	-0.60164	0.268278	-4.272794		
C	1.139011	-1.259029	-3.710864		
C	-0.027353	-0.776712	-4.463284		
C	1.659544	-0.509742	-2.704074		
H	1.524282	-2.240603	-3.957539		
H	2.600347	-0.800499	-2.252343		
H	1.34012	0.52101	-2.582111		
H	-1.163893	-1.259724	-5.881706		
C	1.994205	0.00064	2.544319		
N	2.680002	0.632823	3.21648		

AA2-C3-IP-TS-IP

Atom	X	Y	Z		
O	-1.368053	-1.602819	-1.954063	Electronic Energy (EE)	-728.0211783
O	-1.140139	0.420163	-1.010494	Zero-point Energy Correction	0.236962
C	0.622783	-1.165138	-0.816351	Thermal Correction to Energy	0.252645
C	-0.69078	-0.687051	-1.237513	Thermal Correction to Enthalpy	0.25359
				Thermal Correction to Free Energy	0.192827

C	1.398802	-0.392163	0.194566
H	0.797039	-2.229439	-0.920721
H	2.470287	-0.543917	0.024558
H	1.183911	0.674712	0.09403
H	-2.206393	-1.210669	-2.246274
O	1.905552	0.053465	2.500093
C	1.115556	-0.80048	1.66465
C	1.495338	-2.255163	1.918698
C	-0.328735	-0.534282	2.058301
H	0.863885	-2.936011	1.343163
H	2.539062	-2.434698	1.642662
H	1.371037	-2.486453	2.979436
H	-1.011898	-1.169327	1.489233
H	-0.590053	0.511131	1.87811
H	-0.463942	-0.752865	3.12051
H	2.838037	-0.117756	2.318392
O	-0.514451	-1.445107	-5.397801
O	-0.465071	0.452839	-4.208441
C	1.107165	-1.272144	-3.728042
C	-0.014325	-0.651076	-4.438106
C	1.707873	-0.623672	-2.695099
H	1.383094	-2.280169	-4.012825
H	2.609643	-1.035829	-2.257887
H	1.502949	0.428773	-2.52234
H	-1.24722	-0.984415	-5.836311

AA2-CM-C3-TS-IP

Atom	X	Y	Z		
O	-1.533875	0.12038	-0.891838	Electronic Energy (EE)	-959.0179684
O	0.252974	1.471098	-0.901201	Zero-point Energy Correction	0.317703
C	0.519592	-0.821103	-0.303481	Thermal Correction to Energy	0.338011
C	-0.226617	0.370096	-0.722326	Thermal Correction to Enthalpy	0.338955
C	1.851096	-0.736459	-0.055859	Thermal Correction to Free Energy	0.265269
H	-0.042105	-1.732631	-0.139173		
H	2.426267	-1.631506	0.151415		
H	2.399035	0.155261	-0.346933		
H	-1.97702	0.939044	-1.165759		
O	0.056826	0.957447	2.184852		
O	1.923382	2.176413	1.90494		
C	2.092775	-0.176292	2.116431		
C	1.387643	1.094615	2.063648		
C	3.576546	-0.152035	2.22258		
H	1.560316	-1.033034	2.511417		
H	4.005476	0.51945	1.469859		
H	3.850035	0.250824	3.211957		

H	-0.356334	1.831294	2.095545
O	4.052751	-1.474061	2.068783
C	5.417982	-1.693158	2.465074
C	6.348034	-0.756178	1.69714
H	6.26908	0.278419	2.037991
H	7.386179	-1.073479	1.80894
H	6.094121	-0.799821	0.635081
C	5.538078	-1.550446	3.983888
C	4.494033	-2.00445	4.796567
C	6.679767	-1.029007	4.59283
C	4.586929	-1.933697	6.181962
H	3.59863	-2.408144	4.335761
C	6.777226	-0.962677	5.982389
H	7.505633	-0.668299	3.990776
C	5.732	-1.412267	6.781337
H	3.764676	-2.285574	6.795401
H	7.672459	-0.553282	6.437374
H	5.806176	-1.356145	7.861551
C	5.693294	-3.142361	2.072285
H	5.61212	-3.255917	0.988107
H	6.698798	-3.432052	2.384168
H	4.972703	-3.807361	2.554037

AA2-CM-C3-TS-TL

Atom	X	Y	Z		
O	-1.608584	-0.206992	-0.798569	Electronic Energy (EE)	-959.0052882
O	0.017702	1.327693	-0.892668	Zero-point Energy Correction	0.318593
C	0.545903	-0.897231	-0.216076	Thermal Correction to Energy	0.338978
C	-0.330053	0.193927	-0.662054	Thermal Correction to Enthalpy	0.339922
C	1.869699	-0.670364	-0.026138	Thermal Correction to Free Energy	0.265617
H	0.085576	-1.853098	0.00102		
H	2.546129	-1.489774	0.188423		
H	2.302007	0.264173	-0.3722		
H	-2.122442	0.555851	-1.099504		
O	0.157029	1.173324	2.146685		
O	2.068053	2.307495	1.818627		
C	2.153232	-0.037341	2.122319		
C	1.49585	1.259206	2.017473		
C	3.638414	-0.061639	2.215753		
H	1.597913	-0.857477	2.559873		
H	4.077398	0.608173	1.467262		
H	3.936356	0.320573	3.206369		
H	-0.205323	2.051931	1.961226		
O	4.071571	-1.394883	2.041935		
C	5.420236	-1.668759	2.450358		

C	6.393759	-0.746185	1.717659
H	6.343823	0.283241	2.078746
H	7.419775	-1.098612	1.835804
H	6.156258	-0.757727	0.651286
C	5.522563	-1.565825	3.974106
C	4.456728	-2.02174	4.755997
C	6.659733	-1.078211	4.616603
C	4.524691	-1.98702	6.143132
H	3.565683	-2.397547	4.264794
C	6.732278	-1.047694	6.008268
H	7.501342	-0.714652	4.039056
C	5.666068	-1.499732	6.775583
H	3.686219	-2.340206	6.732648
H	7.624329	-0.663772	6.489915
H	5.720821	-1.471363	7.857585
C	5.652305	-3.116558	2.024898
H	5.582895	-3.200515	0.937659
H	6.64032	-3.451856	2.346135
H	4.900425	-3.765909	2.477528

AA2-TB-C3-TS-IP

Atom	X	Y	Z		
O	-0.842595	0.746384	-1.421024	Zero-point Energy Correction	0.264449
O	0.813695	1.974357	-0.545292	Thermal Correction to Energy	0.281648
C	0.993593	-0.403053	-0.552378	Thermal Correction to Enthalpy	0.282592
C	0.349723	0.886842	-0.821788	Thermal Correction to Free Energy	0.21781
C	2.204816	-0.444322	0.057421		
H	0.438981	-1.3007	-0.797428		
H	2.729476	-1.386585	0.167588		
H	2.78192	0.468741	0.174084		
H	-1.223003	1.625655	-1.5747		
O	-0.177407	0.649922	2.129221		
O	1.655836	1.823538	2.685779		
C	1.846183	-0.500885	2.283261		
C	1.133684	0.761079	2.399683		
C	3.254311	-0.556908	2.763501		
H	1.262321	-1.413273	2.277529		
H	3.833543	0.261979	2.323022		
H	3.257883	-0.402204	3.854436		
H	-0.580686	1.531716	2.175527		
O	3.785037	-1.823254	2.435933		
C	5.225123	-1.945526	2.456396		
C	5.795344	-1.42459	3.772789		
H	6.863598	-1.648525	3.820497		
H	5.676779	-0.34228	3.86691		

Note: the header row of the table above also carries "Electronic Energy (EE) | -767.3063912" in its last two columns.

H	5.303513	-1.908715	4.621047
C	5.469505	-3.442107	2.324647
H	5.011871	-3.820735	1.407022
H	6.541242	-3.64939	2.289175
H	5.03837	-3.974541	3.176131
C	5.82483	-1.205031	1.2625
H	6.905748	-1.364073	1.235643
H	5.398061	-1.582728	0.329573
H	5.651153	-0.127821	1.318231

AA2-TB-C3-TS-TL

Atom	X	Y	Z		
O	-0.993183	0.460838	-1.416951	Electronic Energy (EE)	-767.2928144
O	0.570441	1.871518	-0.659434	Zero-point Energy Correction	0.264927
C	0.951514	-0.479158	-0.527351	Thermal Correction to Energy	0.28232
C	0.19844	0.738696	-0.854163	Thermal Correction to Enthalpy	0.283264
C	2.176831	-0.386078	0.045912	Thermal Correction to Free Energy	0.217281
H	0.468288	-1.431254	-0.708235		
H	2.787599	-1.269907	0.191056		
H	2.666421	0.582319	0.097396		
H	-1.427593	1.303746	-1.610263		
O	-0.092536	0.82687	2.152516		
O	1.791317	1.939869	2.660896		
C	1.894783	-0.39273	2.285877		
C	1.230831	0.899096	2.397318		
C	3.314054	-0.48605	2.727591		
H	1.286039	-1.287379	2.320223		
H	3.891582	0.341745	2.299565		
H	3.3461	-0.363922	3.822402		
H	-0.432453	1.733533	2.147107		
O	3.811615	-1.746975	2.345972		
C	5.234351	-1.944794	2.451889		
C	5.734524	-1.584573	3.849247		
H	6.781518	-1.878681	3.951962		
H	5.673056	-0.51049	4.040427		
H	5.153604	-2.111847	4.610538		
C	5.415448	-3.433697	2.189562		
H	5.011604	-3.694808	1.208662		
H	6.47406	-3.701045	2.213002		
H	4.88849	-4.017794	2.94715		
C	5.959924	-1.128215	1.382438		
H	7.026057	-1.367345	1.391709		
H	5.563963	-1.365663	0.391855		
H	5.865759	-0.052858	1.549059		

AA2-TL-C3-TS-TL

Atom	X	Y	Z		
				Electronic Energy (EE)	-805.1850075
O	-1.074019	0.937495	-1.344664	Zero-point Energy Correction	0.258181
O	0.988976	1.73514	-0.99986	Thermal Correction to Energy	0.274887
C	0.49178	-0.520883	-0.409844	Thermal Correction to Enthalpy	0.275832
C	0.202168	0.82116	-0.930609	Thermal Correction to Free Energy	0.209928
C	1.731582	-0.815928	0.056899		
H	-0.326123	-1.229295	-0.367772		
H	1.984249	-1.83756	0.314906		
H	2.550499	-0.126629	-0.125437		
H	-1.190669	1.837433	-1.681068		
O	0.404128	1.541401	1.967697		
O	2.619811	1.888387	2.072241		
C	1.787515	-0.323505	2.255656		
C	1.680293	1.126071	2.100908		
C	3.106615	-0.892555	2.655059		
H	0.877549	-0.850192	2.518924		
H	3.903887	-0.427454	2.069096		
H	0.431592	2.487846	1.764778		
H	3.128592	-1.968989	2.465135		
C	3.391119	-0.643398	4.155988		
H	3.354915	0.432498	4.344308		
H	2.607812	-1.114209	4.756144		
C	4.741282	-1.189335	4.545862		
C	5.89673	-0.439723	4.313349		
C	4.870119	-2.465779	5.094586		
C	7.151129	-0.952524	4.625342		
H	5.807122	0.55545	3.887871		
C	6.124075	-2.982755	5.408035		
H	3.97948	-3.057362	5.283665		
C	7.268349	-2.227141	5.173464		
H	8.037843	-0.355965	4.443407		
H	6.206642	-3.97437	5.8384		
H	8.245283	-2.626888	5.419208		

AA3-C2-IP-TS-IP

Atom	X	Y	Z		
				Electronic Energy (EE)	-995.2107246
O	-1.238754	-1.162045	0.933385	Zero-point Energy Correction	0.309666
O	-1.049342	1.01662	1.373587	Thermal Correction to Energy	0.330728
C	0.920158	-0.373514	1.542352	Thermal Correction to Enthalpy	0.331672
C	-0.539563	-0.075873	1.282316	Thermal Correction to Free Energy	0.257218
C	1.79742	0.659176	0.831578		
H	1.126337	-1.367997	1.133863		
H	2.832711	0.515125	1.149757		

H	1.493623	1.667918	1.122824
H	-2.168877	-0.912324	0.804124
O	2.528676	-0.966021	3.240277
C	1.197377	-0.471219	3.074639
C	0.213289	-1.432614	3.735604
C	1.174468	0.883583	3.765853
H	-0.805726	-1.036787	3.714186
H	0.218203	-2.402805	3.229739
H	0.499982	-1.579928	4.779235
H	0.214477	1.38116	3.617792
H	1.968384	1.529006	3.385519
H	1.332062	0.741522	4.837639
H	2.565968	-1.868883	2.900012
O	4.682859	2.369313	-1.883133
O	4.543657	0.234591	-1.201721
C	2.562136	1.530037	-1.377401
C	3.998356	1.292395	-1.458309
C	1.703496	0.517617	-0.699074
H	2.243939	2.565944	-1.395462
H	0.665168	0.655425	-1.017514
H	2.015482	-0.490006	-0.987981
H	5.622072	2.134964	-1.952978
O	4.755134	2.604139	-5.418903
O	4.708987	0.566063	-4.490709
C	2.805902	1.986925	-4.291869
C	4.15924	1.623779	-4.722388
C	2.077948	1.124776	-3.536862
H	2.45122	2.980301	-4.538113
H	1.041054	1.346359	-3.312175
H	2.408131	0.097107	-3.417333
H	5.643283	2.310355	-5.676498

AA3-C3-AI-TS-IP

Atom	X	Y	Z	Electronic Energy (EE)	-1012.223424
O	-1.034287	0.914982	-1.036208	Zero-point Energy Correction	0.303888
O	1.090747	1.636176	-0.987009	Thermal Correction to Energy	0.325727
C	0.57397	-0.528696	-0.161939	Thermal Correction to Enthalpy	0.326672
C	0.268376	0.772723	-0.745218	Thermal Correction to Free Energy	0.250095
C	1.899919	-0.760445	0.482139		
H	-0.276883	-1.114431	0.168643		
H	2.128704	-1.82844	0.457398		
H	2.688602	-0.228163	-0.056994		
H	-1.169834	1.775869	-1.463272		
O	0.412776	1.484813	2.149312		
O	2.57493	2.001734	1.933873		

C	1.927484	-0.330124	1.977089
C	1.708251	1.168139	2.031767
C	3.264093	-0.730985	2.605283
H	1.088326	-0.813723	2.481622
H	4.029589	-0.016039	2.292254
H	0.316915	2.451387	2.114409
O	-2.329771	-1.246399	-3.709484
O	-1.852368	-2.615952	-2.002213
C	-0.131182	-1.251399	-2.926206
C	-1.491757	-1.786788	-2.812276
C	0.838955	-1.672762	-2.073959
H	0.034493	-0.452298	-3.638872
H	1.858051	-1.324957	-2.203806
H	0.684568	-2.566975	-1.479207
H	-3.212564	-1.631136	-3.588268
H	3.553076	-1.710778	2.216544
C	3.243252	-0.823857	4.151946
C	2.385557	-2.003242	4.639483
H	1.337104	-1.82169	4.396426
H	2.701307	-2.936661	4.168923
H	2.467726	-2.109697	5.723054
C	2.788764	0.480606	4.825204
H	3.385348	1.330409	4.489115
H	1.736782	0.665773	4.591635
H	2.87703	0.393459	5.909978
C	4.632243	-1.084972	4.566958
N	5.716493	-1.292429	4.890811

AA3-C3-AI-TS-TL

Atom	X	Y	Z		
O	-1.04961	0.916867	-1.066694	Electronic Energy (EE)	-1012.201043
O	1.05976	1.676605	-0.996274	Zero-point Energy Correction	0.304944
C	0.577044	-0.497419	-0.174979	Thermal Correction to Energy	0.326809
C	0.257245	0.801601	-0.760583	Thermal Correction to Enthalpy	0.327753
C	1.907699	-0.706243	0.469378	Thermal Correction to Free Energy	0.25013
H	-0.266752	-1.093973	0.154635		
H	2.165965	-1.767541	0.425289		
H	2.67957	-0.14342	-0.063139		
H	-1.176957	1.77621	-1.49361		
O	0.440336	1.540272	2.108366		
O	2.621165	2.021658	2.029081		
C	1.933465	-0.297548	1.970509		
C	1.742248	1.205524	2.048827		
C	3.259665	-0.723739	2.605286		
H	1.08224	-0.774202	2.462535		

H	4.030649	-0.006245	2.31298
H	0.38252	2.507478	2.072237
O	-2.355131	-1.357563	-3.664531
O	-1.792767	-2.659261	-1.932068
C	-0.139238	-1.263938	-2.925711
C	-1.480023	-1.844676	-2.767233
C	0.856997	-1.643367	-2.085157
H	-0.012951	-0.472462	-3.654102
H	1.864586	-1.271483	-2.233975
H	0.730585	-2.538204	-1.485332
H	-3.213148	-1.771019	-3.491577
H	3.549075	-1.697271	2.200867
C	3.230236	-0.846302	4.150039
C	2.339756	-2.012436	4.611295
H	1.294414	-1.801813	4.377992
H	2.629277	-2.945326	4.123283
H	2.419756	-2.148297	5.691412
C	2.799971	0.456225	4.842808
H	3.414523	1.29865	4.523156
H	1.75431	0.672574	4.607784
H	2.88056	0.350595	5.926245
C	4.611274	-1.150883	4.567681
N	5.686473	-1.396593	4.892213

AA3-C3-IP-TS-IP

Atom	X	Y	Z		
O	-1.04624	0.900765	-1.036867	Zero-point Energy Correction	0.309339
O	1.073254	1.636924	-0.98249	Thermal Correction to Energy	0.330548
C	0.571149	-0.534263	-0.164066	Thermal Correction to Enthalpy	0.331492
C	0.257162	0.766667	-0.74378	Thermal Correction to Free Energy	0.256354
C	1.895538	-0.758688	0.485768		
H	-0.276897	-1.127238	0.160944		
H	2.133277	-1.824616	0.45644		
H	2.682881	-0.217349	-0.046296		
H	-1.186209	1.761861	-1.462032		
O	0.386257	1.469737	2.167807		
O	2.542907	1.999688	1.936269		
C	1.91116	-0.337241	1.983283		
C	1.68352	1.159072	2.040989		
C	3.238052	-0.736738	2.630454		
H	1.068069	-0.825222	2.477627		
H	4.012666	-0.02693	2.325556		
H	0.286707	2.435864	2.134453		
O	-2.310755	-1.243042	-3.731623		
O	-1.839515	-2.62066	-2.029128		

Electronic Energy (EE) -995.2105116

C	-0.116632	-1.246683	-2.935847
C	-1.476226	-1.786111	-2.832519
C	0.849465	-1.668431	-2.079021
H	0.05113	-0.443906	-3.643896
H	1.868286	-1.317091	-2.201177
H	0.694398	-2.565693	-1.489078
H	-3.193253	-1.630437	-3.61699
H	3.529724	-1.72032	2.251197
C	3.226335	-0.820849	4.161744
O	4.597022	-1.101186	4.492334
H	4.662659	-1.192186	5.45076
C	2.353677	-1.971122	4.658043
H	1.29621	-1.796167	4.447652
H	2.656111	-2.909477	4.186023
H	2.462711	-2.076582	5.741672
C	2.811751	0.490157	4.82389
H	3.4397	1.314187	4.477762
H	1.76415	0.730095	4.618475
H	2.918912	0.401392	5.909173

AA3-CM-TS-IP

Atom	X	Y	Z		
O	-1.463901	0.177346	-0.473375	Electronic Energy (EE)	-1226.210183
O	0.244267	1.611532	-0.724885	Zero-point Energy Correction	0.390614
C	0.690146	-0.63551	-0.10197	Thermal Correction to Energy	0.416315
C	-0.160497	0.497362	-0.448306	Thermal Correction to Enthalpy	0.417259
C	2.122014	-0.399671	0.239898	Thermal Correction to Free Energy	0.330099
H	0.186676	-1.503048	0.310442		
H	2.699042	-1.304843	0.039704		
H	2.531714	0.41206	-0.367908		
H	-1.97366	0.954926	-0.751213		
O	0.376113	1.104898	2.388783		
O	2.198835	2.337956	1.998089		
C	2.333377	-0.055924	1.738588		
C	1.662009	1.257523	2.054639		
C	3.819183	0.034912	2.065329		
H	1.872468	-0.834884	2.351438		
H	4.302126	0.768041	1.410408		
H	3.946384	0.361773	3.104182		
H	-0.024889	1.979219	2.527308		
O	4.373417	-1.253805	1.872687		
O	-2.511546	-2.245668	-3.033606		
O	-1.189768	-3.392875	-1.636187		
C	-0.332994	-1.459806	-2.734599		
C	-1.348034	-2.464455	-2.402335		

In the printed page, the Electronic Energy (EE) line appears on the header row (Atom X Y Z) and the other energy lines on the following rows, each shifted up by one row relative to this table.

C	0.894181	-1.517811	-2.155543
H	-0.634322	-0.632875	-3.366451
H	1.662352	-0.809769	-2.446907
H	1.212145	-2.424331	-1.651282
H	-3.146474	-2.928755	-2.764711
C	5.665791	-1.468996	2.462671
C	5.537733	-1.469463	3.988273
C	6.542136	-0.973312	4.819602
C	4.400074	-2.031743	4.576623
C	6.413979	-1.037194	6.206948
H	7.435668	-0.530575	4.395192
C	4.26775	-2.091358	5.959369
H	3.609433	-2.418086	3.942191
C	5.277125	-1.593912	6.781733
H	7.205651	-0.645583	6.836273
H	3.375808	-2.526617	6.396696
H	5.175409	-1.639205	7.860226
C	6.067695	-2.865368	1.994109
H	6.167554	-2.878578	0.905619
H	7.021879	-3.152663	2.440576
H	5.310056	-3.594827	2.289917
C	6.663539	-0.435084	1.943914
H	7.683854	-0.725822	2.199639
H	6.588513	-0.388786	0.854674
H	6.478768	0.560484	2.353394

AA3-CM-TS-TL

Atom	X	Y	Z		
O	-1.463745	0.204044	-0.549869	Electronic Energy (EE)	-1226.191922
O	0.244543	1.634563	-0.802212	Zero-point Energy Correction	0.391983
C	0.683555	-0.586066	-0.086678	Thermal Correction to Energy	0.417581
C	-0.158528	0.534255	-0.496414	Thermal Correction to Enthalpy	0.418525
C	2.106117	-0.329114	0.279539	Thermal Correction to Free Energy	0.331755
H	0.170405	-1.439599	0.342741		
H	2.706027	-1.222998	0.094486		
H	2.506353	0.488782	-0.326576		
H	-1.948813	0.977859	-0.870015		
O	0.340624	1.239769	2.307927		
O	2.238524	2.406519	2.129753		
C	2.302405	0.020928	1.778489		
C	1.662291	1.35314	2.087646		
C	3.786047	0.074523	2.122016		
H	1.816704	-0.746656	2.387223		
H	4.283845	0.821391	1.49411		
H	3.91175	0.370857	3.17043		

H	-0.016465	2.132741	2.428845
O	4.314609	-1.21637	1.894533
O	-2.421472	-2.38925	-3.035871
O	-1.118594	-3.426765	-1.539939
C	-0.271473	-1.532508	-2.708609
C	-1.272739	-2.546547	-2.35271
C	0.941145	-1.545066	-2.098278
H	-0.572486	-0.740844	-3.383434
H	1.709256	-0.842268	-2.401212
H	1.252604	-2.430329	-1.554484
H	-3.033328	-3.078713	-2.74075
C	5.61481	-1.463835	2.445586
C	5.526445	-1.492555	3.973986
C	6.543922	-1.005141	4.792963
C	4.4047	-2.071466	4.57533
C	6.445222	-1.094846	6.180548
H	7.424223	-0.54707	4.357868
C	4.301542	-2.157134	5.958079
H	3.605732	-2.448589	3.946132
C	5.324762	-1.66896	6.767657
H	7.24649	-0.709123	6.80056
H	3.421974	-2.605514	6.406125
H	5.246085	-1.73456	7.846576
C	5.979301	-2.856897	1.937109
H	6.048722	-2.848153	0.846674
H	6.937222	-3.174868	2.35324
H	5.214429	-3.576519	2.235473
C	6.619474	-0.437849	1.921564
H	7.640984	-0.752878	2.141288
H	6.516037	-0.365922	0.836347
H	6.467537	0.552434	2.356066

AA3-TB-TS-IP

Atom	X	Y	Z		
				Electronic Energy (EE)	-1034.498614
O	-1.357954	0.3034	-0.673885	Zero-point Energy Correction	0.337445
O	0.377628	1.695746	-0.380656	Thermal Correction to Energy	0.359979
C	0.689029	-0.637581	-0.070793	Thermal Correction to Enthalpy	0.360923
C	-0.077948	0.567115	-0.367699	Thermal Correction to Free Energy	0.282447
C	2.038024	-0.511483	0.550726		
H	0.113608	-1.541258	0.097984		
H	2.636924	-1.392618	0.309776		
H	2.554168	0.372579	0.165278		
H	-1.805309	1.134422	-0.899611		
O	-0.04443	0.695845	2.583702		
O	1.832133	1.902032	2.720446		

C	1.985718	-0.421785	2.098734
C	1.281966	0.848204	2.506307
C	3.396078	-0.439816	2.678619
H	1.418098	-1.271225	2.487432
H	3.995363	0.341748	2.201011
H	3.356597	-0.232143	3.754147
H	-0.453927	1.552927	2.78856
O	3.93107	-1.726285	2.439818
O	-1.925523	-1.527869	-3.807553
O	-0.949535	-2.979368	-2.408236
C	0.167168	-0.945711	-2.95376
C	-0.922275	-1.925408	-3.010171
C	1.253973	-1.179712	-2.173423
H	0.021824	-0.005601	-3.472496
H	2.085746	-0.483687	-2.186495
H	1.439482	-2.177	-1.788494
H	-2.619784	-2.206283	-3.801501
C	5.368384	-1.853723	2.487978
C	5.924006	-1.245276	3.773002
H	6.991505	-1.465203	3.849322
H	5.804102	-0.159247	3.792089
H	5.421261	-1.670734	4.645993
C	5.989367	-1.20086	1.254411
H	7.06893	-1.371188	1.249418
H	5.567582	-1.636644	0.344681
H	5.824512	-0.121095	1.236017
C	5.608674	-3.356892	2.464381
H	6.679819	-3.570539	2.460157
H	5.161877	-3.827318	3.343917
H	5.163086	-3.797137	1.568528

AA3-TB-TS-TL

Atom	X	Y	Z		
O	-1.367557	0.35037	-0.708572	Electronic Energy (EE)	-1034.479563
O	0.363392	1.752851	-0.452437	Zero-point Energy Correction	0.338249
C	0.684871	-0.570892	-0.085947	Thermal Correction to Energy	0.360922
C	-0.083692	0.628016	-0.408594	Thermal Correction to Enthalpy	0.361866
C	2.035865	-0.423631	0.527591	Thermal Correction to Free Energy	0.282046
H	0.111461	-1.471381	0.105328		
H	2.655143	-1.289237	0.280565		
H	2.525324	0.476273	0.143786		
H	-1.797791	1.182074	-0.952939		
O	-0.010153	0.821419	2.517288		
O	1.900732	1.936924	2.830948		
C	2.002067	-0.351064	2.076614		

C	1.330265	0.926281	2.519021
C	3.41747	-0.409107	2.641042
H	1.425258	-1.198117	2.45753
H	4.013442	0.401154	2.206817
H	3.38512	-0.259519	3.726554
H	-0.37541	1.692292	2.735573
O	3.938015	-1.678743	2.321165
O	-1.955947	-1.787303	-3.708704
O	-0.86598	-3.085124	-2.246302
C	0.119356	-1.03181	-2.941539
C	-0.912863	-2.076405	-2.909164
C	1.233547	-1.168517	-2.177937
H	-0.089808	-0.131636	-3.506163
H	2.030999	-0.436757	-2.244425
H	1.472256	-2.139246	-1.757093
H	-2.596885	-2.507658	-3.624212
C	5.357934	-1.859717	2.460912
C	5.830258	-1.427511	3.847825
H	6.880264	-1.697871	3.981245
H	5.745652	-0.34718	3.988362
H	5.245142	-1.929171	4.623093
C	6.098036	-1.091239	1.365796
H	7.164891	-1.324918	1.401824
H	5.716434	-1.377618	0.382462
H	5.995902	-0.009888	1.479649
C	5.557044	-3.358125	2.275712
H	6.61694	-3.615228	2.334332
H	5.018385	-3.909062	3.049774
H	5.175639	-3.670647	1.300881

AA3-TL-TS-TL

Atom	X	Y	Z		
O	-1.111044	0.862646	-0.927228	Electronic Energy (EE)	-1072.37083
O	0.958386	1.7151	-0.783573	Zero-point Energy Correction	0.331126
C	0.586803	-0.549062	-0.173538	Thermal Correction to Energy	0.35316
C	0.201216	0.782385	-0.632642	Thermal Correction to Enthalpy	0.354104
C	1.92443	-0.746631	0.457889	Thermal Correction to Free Energy	0.27448
H	-0.225134	-1.218813	0.087923		
H	2.248862	-1.780012	0.306802		
H	2.661633	-0.086687	-0.007753		
H	-1.28155	1.753328	-1.265332		
O	0.363468	1.268269	2.277203		
O	2.527622	1.813024	2.376964		
C	1.925939	-0.496721	1.991034		
C	1.676589	0.976419	2.242107		

C	3.255236	-0.931155	2.605092
H	1.098982	-1.058457	2.436063
H	4.059188	-0.316038	2.190281
H	0.277666	2.230048	2.362397
O	-2.311527	-1.375515	-3.717021
O	-1.610371	-2.750376	-2.095405
C	-0.107639	-1.128035	-2.978684
C	-1.384395	-1.845624	-2.863327
C	0.93406	-1.484117	-2.18442
H	-0.06895	-0.268098	-3.635716
H	1.900001	-1.007192	-2.308139
H	0.902467	-2.435926	-1.665374
H	-3.12363	-1.882927	-3.575771
H	3.454044	-1.966675	2.312604
C	3.269249	-0.829803	4.135734
H	3.056334	0.200811	4.433328
H	2.476988	-1.461976	4.548407
C	4.602587	-1.249248	4.702684
C	5.672212	-0.350831	4.724384
C	4.811578	-2.548031	5.168065
C	6.918958	-0.740089	5.201735
H	5.52051	0.662954	4.365398
C	6.058155	-2.942076	5.646845
H	3.988051	-3.255636	5.159926
C	7.115766	-2.038689	5.664591
H	7.737221	-0.029004	5.21578
H	6.201657	-3.9538	6.009138
H	8.086198	-2.342676	6.039449

AM-C2-IP-TS-IP

Atom	X	Y	Z		
O	-1.110134	0.966533	-0.47181	Zero-point Energy Correction	0.17538
C	0.75112	-0.343987	0.266469	Thermal Correction to Energy	0.186624
C	-0.58794	-0.143886	-0.354046	Thermal Correction to Enthalpy	0.187569
C	1.675043	0.658713	0.185726	Thermal Correction to Free Energy	0.138481
H	1.082468	-1.373526	0.375694		
H	2.706479	0.491726	0.471894		
H	1.382671	1.661069	-0.105883		
N	-1.193582	-1.264658	-0.798065		
H	-0.767583	-2.172861	-0.692611		
H	-2.115003	-1.202719	-1.208396		
O	1.260783	-0.863918	2.986043		
C	0.082516	-0.455683	2.411229		
C	-1.022954	-1.46143	2.433738		
C	-0.217025	0.962165	2.753598		

Electronic Energy (EE): -440.9526259

H	-1.908864	-1.080767	1.924341
H	-0.719485	-2.400999	1.960533
H	-1.296143	-1.682048	3.475281
H	-1.068291	1.329242	2.179359
H	0.651426	1.594643	2.550388
H	-0.456653	1.044385	3.822186
H	1.368603	-1.817483	2.876461

AM-C3-AI-TS-IP

Atom	X	Y	Z		
O	1.861011	1.992685	-2.184946	Zero-point Energy Correction	0.17038
C	2.62002	-0.089633	-1.306637	Thermal Correction to Energy	0.182227
C	1.607323	0.826827	-1.871845	Thermal Correction to Enthalpy	0.183172
C	3.880821	0.342846	-1.035169	Thermal Correction to Free Energy	0.13175
H	2.313674	-1.104972	-1.077074		
H	4.654663	-0.375953	-0.789507		
H	4.200431	1.313843	-1.400593		
N	0.375017	0.296567	-2.02436		
H	0.181078	-0.665092	-1.787764		
H	-0.360241	0.856277	-2.433142		
C	3.852788	1.050734	1.070902		
C	3.521893	-0.202039	1.82905		
H	4.258814	-0.988769	1.660495		
H	3.491771	0.017127	2.903169		
H	2.534373	-0.560035	1.530792		
C	2.832214	2.15018	1.053975		
H	2.7424	2.578346	2.059963		
H	3.10157	2.948938	0.361872		
H	1.85746	1.743382	0.77552		
C	5.215765	1.454793	1.089073		
N	6.329406	1.766077	1.056121		

Electronic Energy (EE): -457.973541

AM-C3-AI-TS-TL

Atom	X	Y	Z		
O	1.781735	2.005837	-2.042151	Zero-point Energy Correction	0.170577
C	2.606916	-0.095284	-1.295686	Thermal Correction to Energy	0.182551
C	1.571306	0.822919	-1.82526	Thermal Correction to Enthalpy	0.183495
C	3.861005	0.36074	-1.033327	Thermal Correction to Free Energy	0.131695
H	2.332582	-1.12603	-1.095578		
H	4.660268	-0.340196	-0.821064		
H	4.141874	1.347424	-1.387335		
N	0.352209	0.250819	-2.036338		
H	0.218627	-0.744961	-1.966705		
H	-0.36602	0.801355	-2.482384		

Electronic Energy (EE): -457.9613356

C	3.86938	1.057046	1.077464
C	3.505995	-0.186734	1.83775
H	4.219734	-0.994332	1.667674
H	3.485868	0.027124	2.91302
H	2.508355	-0.518949	1.543282
C	2.873369	2.179385	1.059705
H	2.809754	2.630448	2.057688
H	3.142693	2.956688	0.344623
H	1.885559	1.793901	0.800255
C	5.244415	1.424851	1.098109
N	6.366628	1.700078	1.066152

AM-C3-CM-TS-IP

Atom	X	Y	Z		
O	-0.839923	1.775365	-1.891344	Electronic Energy (EE)	-671.9471061
C	0.500038	-0.029424	-1.099359	Zero-point Energy Correction	0.258276
C	-0.689065	0.858408	-1.084531	Thermal Correction to Energy	0.273392
C	1.502493	0.175971	-1.993935	Thermal Correction to Enthalpy	0.274336
H	0.568632	-0.804462	-0.343097	Thermal Correction to Free Energy	0.21479
H	2.319979	-0.52914	-2.071929		
H	1.350719	0.857646	-2.821312		
H	-2.427913	1.14505	-0.048003		
O	2.58854	1.646525	-1.103329		
C	3.11727	1.413572	0.183416		
C	4.281263	2.426816	0.214357		
C	2.073537	1.687722	1.263285		
C	3.67441	-0.006662	0.33173		
C	4.382675	3.424519	1.183741		
C	5.273433	2.340656	-0.769779		
C	5.466513	4.302403	1.183844		
C	6.349895	3.21909	-0.772648		
C	6.452306	4.203333	0.208918		
H	1.278264	0.943446	1.187513		
H	2.507545	1.615665	2.263505		
H	1.631883	2.678521	1.135402		
H	2.858491	-0.733724	0.340827		
H	4.219714	-0.093082	1.274781		
H	4.353802	-0.249647	-0.487347		
H	3.626873	3.526315	1.952317		
H	5.198811	1.58341	-1.541999		
H	5.534206	5.066465	1.950431		
H	7.109192	3.136835	-1.542465		
H	7.292386	4.888766	0.20962		
N	-1.588346	0.586655	-0.118889		
H	-1.452056	-0.172846	0.531732		

AM-C3-CM-TS-TL

Atom	X	Y	Z		
O	-0.936824	1.632925	-1.997419	Electronic Energy (EE)	-671.9343851
C	0.503409	-0.042836	-1.112728	Zero-point Energy Correction	0.258104
C	-0.721931	0.799775	-1.135653	Thermal Correction to Energy	0.273515
C	1.515873	0.19538	-1.987999	Thermal Correction to Enthalpy	0.274459
H	0.586451	-0.813488	-0.35264	Thermal Correction to Free Energy	0.214027
H	2.348153	-0.491275	-2.068112		
H	1.346572	0.880387	-2.808595		
H	-2.450155	1.084258	-0.071872		
O	2.596485	1.643242	-1.104872		
C	3.110743	1.433097	0.18428		
C	4.284044	2.435291	0.216462		
C	2.058124	1.73219	1.250794		
C	3.664651	0.013144	0.365608		
C	4.426601	3.395186	1.216817		
C	5.246773	2.370976	-0.796326		
C	5.522125	4.257139	1.2185		
C	6.334893	3.233451	-0.797824		
C	6.478659	4.179557	0.21435		
H	1.242737	1.012527	1.157265		
H	2.469328	1.645857	2.259567		
H	1.643182	2.732285	1.112096		
H	2.851108	-0.716984	0.371429		
H	4.197573	-0.06192	1.316345		
H	4.35842	-0.239165	-0.438494		
H	3.690294	3.483037	2.005394		
H	5.130128	1.647719	-1.59474		
H	5.620528	4.993954	2.007578		
H	7.069669	3.171081	-1.592379		
H	7.327127	4.853937	0.215053		
N	-1.587843	0.562817	-0.11285		
H	-1.411327	-0.139376	0.586416		

AM-C3-IP-TS-IP

Atom	X	Y	Z		
O	-0.611827	1.378039	-0.446783	Electronic Energy (EE)	-440.9533419
C	0.677643	-0.563562	0.086178	Zero-point Energy Correction	0.176268
C	-0.55168	0.151022	-0.327562	Thermal Correction to Energy	0.187521
C	1.800142	0.108215	0.364913	Thermal Correction to Enthalpy	0.188465
H	0.626594	-1.644674	0.170924	Thermal Correction to Free Energy	0.138626
H	2.700649	-0.39957	0.683933		
H	1.842096	1.187304	0.256459		

N	-1.6204	-0.638197	-0.569947
H	-1.568101	-1.64125	-0.475282
H	-2.493793	-0.219909	-0.857852
O	2.127531	-0.784631	3.024154
C	1.992926	0.516187	2.613463
C	0.633514	1.110465	2.719024
C	3.215361	1.323241	2.853402
H	0.593876	2.074423	2.208091
H	-0.119664	0.450891	2.275062
H	0.357969	1.272407	3.772333
H	3.156347	2.274896	2.321773
H	4.108336	0.786927	2.520625
H	3.338869	1.543703	3.924463
H	1.289685	-1.251082	2.904989

AM-C3-TB-TS-IP

Atom	X	Y	Z		
O	1.711579	0.545416	-1.351857	Electronic Energy (EE)	-480.2376634
C	0.702561	-0.666031	0.438081	Zero-point Energy Correction	0.204397
C	0.722292	0.346366	-0.646124	Thermal Correction to Energy	0.216634
C	1.820396	-1.366802	0.776159	Thermal Correction to Enthalpy	0.217578
H	-0.234015	-0.830295	0.961501	Thermal Correction to Free Energy	0.1658
H	1.735104	-2.246832	1.398144		
H	2.721681	-1.263329	0.183025		
H	-0.50089	1.715586	-1.544948		
O	2.481201	-0.455263	2.455918		
C	2.980895	0.857464	2.392076		
C	3.716127	0.986983	3.744132		
C	1.857645	1.891765	2.325931		
C	3.973539	1.033561	1.245688		
H	4.516884	0.248459	3.816485		
H	3.020989	0.844621	4.574173		
H	4.149059	1.988069	3.813399		
H	1.123159	1.701537	3.1127		
H	2.258831	2.899982	2.459328		
H	1.351377	1.8571	1.358494		
H	4.756227	0.27231	1.302181		
H	4.442415	2.01947	1.302083		
H	3.466265	0.950796	0.281889		
N	-0.431212	1.022665	-0.81249		
H	-1.234049	0.852694	-0.224955		

AM-C3-TB-TS-TL

Atom	X	Y	Z	Electronic Energy (EE)	-480.2276097

O	1.710621	0.599088	-1.293894	Zero-point Energy Correction	0.204541
C	0.703052	-0.681208	0.441915	Thermal Correction to Energy	0.216929
C	0.72134	0.354467	-0.620919	Thermal Correction to Enthalpy	0.217874
C	1.834374	-1.360117	0.777344	Thermal Correction to Free Energy	0.165751
H	-0.230109	-0.872739	0.96166		
H	1.774283	-2.246954	1.391283		
H	2.728694	-1.225918	0.180364		
H	-0.519508	1.71788	-1.511261		
O	2.458168	-0.442319	2.446912		
C	2.97691	0.85787	2.381657		
C	3.698471	0.980703	3.741753		
C	1.869033	1.910156	2.301077		
C	3.985243	1.020137	1.245107		
H	4.484503	0.228345	3.824692		
H	2.991883	0.848166	4.56306		
H	4.147824	1.974023	3.817028		
H	1.122827	1.727058	3.077793		
H	2.280076	2.913833	2.439253		
H	1.375866	1.883281	1.327086		
H	4.752509	0.244297	1.309927		
H	4.473657	1.996139	1.308525		
H	3.489813	0.952074	0.273943		
N	-0.455375	1.012172	-0.793517		
H	-1.266095	0.815858	-0.229775		

AM-C3-TL-TS-TL

Atom	X	Y	Z	Electronic Energy (EE)	-518.1252992
O	-0.449465	1.314542	0.134117	Zero-point Energy Correction	0.196544
C	0.902652	-0.609551	-0.193385	Thermal Correction to Energy	0.208448
C	-0.369513	0.140838	-0.200643	Thermal Correction to Enthalpy	0.209393
C	2.061797	0.033184	0.107712	Thermal Correction to Free Energy	0.155834
H	0.876156	-1.682593	-0.35238		
H	3.013924	-0.477136	0.019692		
H	2.078051	1.117276	0.09918		
H	-2.342966	-0.088534	-0.686714		
C	4.586277	2.996681	2.694536		
C	3.408039	2.285462	2.531306		
C	3.409112	0.875433	2.543594		
C	4.642184	0.212584	2.710506		
C	5.817146	0.929468	2.873999		
C	5.796486	2.324306	2.867249		
H	4.565862	4.080522	2.687811		
H	2.467136	2.80892	2.394125		
H	4.660477	-0.872684	2.717452		
H	6.755269	0.403148	3.008594		

H	6.716207	2.882766	2.994973
C	2.195477	0.140006	2.329079
H	2.190706	-0.927081	2.515655
H	1.246032	0.653993	2.417768
N	-1.467269	-0.577019	-0.577894
H	-1.379661	-1.495082	-0.982346

AM2-C2-IP-TS-IP

Atom	X	Y	Z		
O	-1.085821	1.033361	1.151096	Electronic Energy (EE)	-688.264484
C	0.903008	-0.310019	1.383588	Zero-point Energy Correction	0.259272
C	-0.536893	-0.048437	0.956079	Thermal Correction to Energy	0.276347
C	1.784993	0.792367	0.894248	Thermal Correction to Enthalpy	0.277291
H	1.231229	-1.258601	0.942547	Thermal Correction to Free Energy	0.212801
H	2.855766	0.637341	0.941678		
H	1.433863	1.812982	0.984462		
O	2.344019	-0.95162	3.217232		
C	1.006601	-0.53044	2.936037		
C	0.027074	-1.610418	3.382939		
C	0.806197	0.749228	3.731575		
H	-1.008292	-1.279443	3.269541		
H	0.164573	-2.526997	2.80053		
H	0.200084	-1.83921	4.437218		
H	-0.177692	1.178111	3.538483		
H	1.568742	1.487752	3.474969		
H	0.888332	0.523735	4.797666		
H	2.487053	-1.816799	2.813293		
O	4.111297	-0.460371	-1.878126		
C	2.566664	1.351851	-1.971977		
C	3.874861	0.709798	-2.197486		
C	1.552002	0.659917	-1.402654		
H	2.465961	2.403635	-2.218754		
H	0.563631	1.100237	-1.328394		
H	1.632167	-0.4163	-1.287775		
N	-1.160415	-1.069067	0.342146		
H	-0.705092	-1.957376	0.196004		
H	-2.125491	-0.96346	0.058393		
N	4.812889	1.489415	-2.778225		
H	4.621775	2.447735	-3.029408		
H	5.733082	1.112308	-2.955503		

AM2-C3-AI-TS-IP

Atom	X	Y	Z		
O	-0.686169	0.899989	0.101495	Electronic Energy (EE)	-705.2906902
				Zero-point Energy Correction	0.254882

C	0.237893	-1.273072	0.352677	Thermal Correction to Energy	0.272313
C	-0.872195	-0.314914	0.211012	Thermal Correction to Enthalpy	0.273257
C	1.599586	-0.776176	0.712039	Thermal Correction to Free Energy	0.208706
H	-0.026159	-2.290794	0.619051		
H	2.357864	-1.438059	0.283647		
H	1.744799	0.223981	0.297211		
N	-2.111586	-0.856994	0.1937		
H	-2.245235	-1.857524	0.203026		
H	-2.900169	-0.267163	-0.03398		
O	-1.40536	-0.027145	-3.012652		
C	-0.626991	-2.173682	-2.334886		
C	-1.613826	-1.238051	-2.915447		
C	0.543595	-1.719749	-1.815589		
H	-0.891039	-3.22525	-2.287019		
H	1.312347	-2.427115	-1.527233		
H	0.84836	-0.692297	-1.991812		
N	-2.765672	-1.804514	-3.335277		
H	-2.92342	-2.798607	-3.264297		
H	-3.478353	-1.226369	-3.75807		
C	1.843212	-0.72022	2.251176		
C	1.759078	-2.115318	2.887769		
H	0.732527	-2.481027	2.823833		
H	2.419191	-2.820332	2.37766		
H	2.039644	-2.069592	3.941918		
C	0.885806	0.253239	2.95335		
H	0.93639	1.247601	2.506411		
H	-0.13732	-0.12029	2.866579		
H	1.133598	0.326946	4.014311		
C	3.216548	-0.221268	2.430513		
N	4.291173	0.166342	2.566961		

AM2-C3-AI-TS-TL

Atom	X	Y	Z		
O	-0.515528	1.055335	0.233762	Electronic Energy (EE)	-705.2673015
C	0.253753	-1.178879	0.326126	Zero-point Energy Correction	0.255675
C	-0.789897	-0.133699	0.245454	Thermal Correction to Energy	0.272978
C	1.634666	-0.777117	0.73133	Thermal Correction to Enthalpy	0.273922
H	-0.071201	-2.191528	0.541534	Thermal Correction to Free Energy	0.209016
H	2.369388	-1.46318	0.300277		
H	1.837509	0.22715	0.352228		
N	-2.078964	-0.591575	0.21082		
H	-2.251173	-1.557869	-0.024834		
H	-2.771844	0.070967	-0.109943		
O	-1.768565	-0.175389	-2.966661		
C	-0.580867	-2.142383	-2.359128		

C	-1.748316	-1.390428	-2.877682
C	0.507002	-1.484991	-1.876334
H	-0.629644	-3.227033	-2.342872
H	1.4157	-2.036148	-1.6656
H	0.590175	-0.413761	-2.034476
N	-2.824454	-2.165265	-3.211934
H	-2.730049	-3.163869	-3.308939
H	-3.590623	-1.720487	-3.696513
C	1.848757	-0.779307	2.277058
C	1.665799	-2.185861	2.867078
H	0.621765	-2.49049	2.77108
H	2.297061	-2.915557	2.355024
H	1.92265	-2.190911	3.92795
C	0.929792	0.22468	2.987894
H	1.024004	1.221274	2.555629
H	-0.109334	-0.096093	2.886123
H	1.171888	0.269763	4.051587
C	3.247132	-0.371903	2.500997
N	4.341532	-0.06287	2.671044

AM2-C3-IP-TS-IP

Atom	X	Y	Z		
O	-0.708616	0.899748	0.108816	Electronic Energy (EE)	-688.2778173
C	0.225671	-1.270199	0.361284	Zero-point Energy Correction	0.260172
C	-0.887118	-0.317032	0.214624	Thermal Correction to Energy	0.276985
C	1.580618	-0.775616	0.74318	Thermal Correction to Enthalpy	0.27793
H	-0.040914	-2.289213	0.621798	Thermal Correction to Free Energy	0.215338
H	2.347269	-1.431992	0.319148		
H	1.733227	0.229728	0.342379		
N	-2.125702	-0.863974	0.189606		
H	-2.252698	-1.865383	0.179551		
H	-2.912121	-0.277835	-0.054539		
O	-1.412172	-0.0269	-3.016173		
C	-0.621735	-2.168957	-2.3371		
C	-1.610379	-1.240236	-2.92328		
C	0.540417	-1.707797	-1.804323		
H	-0.878381	-3.222638	-2.294841		
H	1.312262	-2.410096	-1.512115		
H	0.839918	-0.677872	-1.974663		
N	-2.754372	-1.814628	-3.355332		
H	-2.905197	-2.809994	-3.287986		
H	-3.467735	-1.240774	-3.782647		
C	1.827907	-0.728621	2.265467		
C	1.734182	-2.116452	2.891483		
H	0.712078	-2.500341	2.854395		

H	2.393333	-2.8166	2.371328
H	2.036864	-2.069262	3.941818
C	0.891084	0.248839	2.966856
H	0.972062	1.243232	2.521833
H	-0.149056	-0.081171	2.898686
H	1.153553	0.315981	4.02716
O	3.178221	-0.252606	2.370677
H	3.39686	-0.177254	3.307528

AM2-CM-TS-IP

Atom	X	Y	Z		
O	-0.664526	0.949082	-0.01335	Electronic Energy (EE)	-919.2707889
C	0.460642	-1.105106	0.336259	Zero-point Energy Correction	0.341875
C	-0.739259	-0.261809	0.216606	Thermal Correction to Energy	0.363124
C	1.775541	-0.420984	0.51864	Thermal Correction to Enthalpy	0.364068
H	0.338399	-2.102255	0.744921	Thermal Correction to Free Energy	0.289272
H	2.598888	-1.110061	0.306722		
H	1.84098	0.430358	-0.163389		
N	-1.921665	-0.900267	0.351721		
H	-1.96722	-1.894949	0.515653		
H	-2.780385	-0.391437	0.194679		
O	1.828264	0.004344	1.880051		
O	-1.576705	-0.410442	-2.957491		
C	-0.472806	-2.373258	-2.177811		
C	-1.622031	-1.620333	-2.728491		
C	0.671584	-1.734794	-1.831287		
H	-0.595216	-3.438897	-2.012441		
H	1.549013	-2.306975	-1.551123		
H	0.814928	-0.696822	-2.116474		
N	-2.728671	-2.356394	-2.964538		
H	-2.751198	-3.348856	-2.783276		
H	-3.547394	-1.912441	-3.356648		
C	2.753891	1.066635	2.194887		
C	2.874825	1.023192	3.717301		
C	3.069031	-0.211371	4.346909		
C	2.847974	2.17422	4.504822		
C	3.228437	-0.293007	5.725231		
H	3.088672	-1.115887	3.749485		
C	3.010088	2.094561	5.888059		
H	2.697263	3.146917	4.052636		
C	3.200528	0.863094	6.50336		
H	3.374426	-1.260154	6.193858		
H	2.983242	3.001288	6.48238		
H	3.32421	0.801745	7.578793		
C	4.131723	0.794219	1.58235		

H	4.491046	-0.198576	1.864513
H	4.102369	0.866795	0.492369
H	4.843883	1.535676	1.951357
C	2.193966	2.390733	1.683216
H	1.234003	2.612781	2.154682
H	2.887463	3.209837	1.883824
H	2.048237	2.346384	0.602491

AM2-CM-TS-TL

Atom	X	Y	Z		
O	-0.742739	1.076311	-0.023285	Electronic Energy (EE)	-919.2492254
C	0.469994	-0.933183	0.243761	Zero-point Energy Correction	0.342431
C	-0.767788	-0.131417	0.153508	Thermal Correction to Energy	0.363664
C	1.755789	-0.197075	0.438001	Thermal Correction to Enthalpy	0.364608
H	0.400653	-1.935871	0.65249	Thermal Correction to Free Energy	0.289849
H	2.603094	-0.790512	0.076298		
H	1.710756	0.740952	-0.121314		
N	-1.932937	-0.832227	0.288551		
H	-1.920726	-1.837808	0.209718		
H	-2.776253	-0.365478	-0.013872		
O	1.886147	0.03055	1.834038		
O	-1.895316	-0.798442	-2.982293		
C	-0.334162	-2.418317	-2.211841		
C	-1.655089	-1.960714	-2.707808		
C	0.646426	-1.521772	-1.939416		
H	-0.186915	-3.479064	-2.032756		
H	1.6518	-1.866477	-1.726911		
H	0.514944	-0.486899	-2.24136		
N	-2.602956	-2.942468	-2.795937		
H	-2.344014	-3.915244	-2.747444		
H	-3.474031	-2.71336	-3.252033		
C	2.726602	1.117227	2.240375		
C	2.901448	0.96477	3.750365		
C	2.310382	-0.081699	4.456792		
C	3.668948	1.896606	4.456539		
C	2.485931	-0.195385	5.834706		
H	1.709282	-0.805117	3.923421		
C	3.845051	1.783279	5.83009		
H	4.136178	2.723554	3.9324		
C	3.253038	0.733158	6.526972		
H	2.016565	-1.01541	6.366653		
H	4.444079	2.517257	6.357108		
H	3.388109	0.643464	7.598625		
C	4.090658	1.027262	1.548731		
H	4.544571	0.050167	1.732014		

H	3.997284	1.182791	0.471197
H	4.763408	1.794292	1.93528
C	2.032867	2.44728	1.931109
H	1.059417	2.47951	2.423225
H	2.635012	3.285259	2.287881
H	1.880933	2.577039	0.857878

AM2-TB-TS-IP

Atom	X	Y	Z		
O	-0.74055	0.904907	0.110319	Electronic Energy (EE)	-727.5622363
C	0.434772	-1.148151	0.258124	Zero-point Energy Correction	0.287964
C	-0.783402	-0.32291	0.236016	Thermal Correction to Energy	0.306289
C	1.746522	-0.463637	0.460579	Thermal Correction to Enthalpy	0.307233
H	0.339301	-2.176694	0.589399	Thermal Correction to Free Energy	0.240192
H	2.565835	-1.104688	0.119821		
H	1.765237	0.46826	-0.110487		
N	-1.949665	-0.996695	0.338652		
H	-1.971908	-2.002806	0.411846		
H	-2.821655	-0.495533	0.241069		
O	1.869913	-0.209958	1.857945		
O	-1.671748	-0.227971	-2.92634		
C	-0.539166	-2.234541	-2.323052		
C	-1.703688	-1.452676	-2.79439		
C	0.60677	-1.612411	-1.953075		
H	-0.650943	-3.310338	-2.233617		
H	1.49378	-2.1951	-1.731981		
H	0.736503	-0.555292	-2.165386		
N	-2.80794	-2.177862	-3.071829		
H	-2.82045	-3.181714	-2.9705		
H	-3.635951	-1.71128	-3.415027		
C	2.828748	0.799248	2.247774		
C	2.924181	0.652359	3.760234		
H	3.297015	-0.340738	4.023636		
H	3.604559	1.401129	4.172152		
H	1.940285	0.788818	4.216263		
C	4.186564	0.537052	1.601179		
H	4.517581	-0.485045	1.80547		
H	4.157721	0.689329	0.519462		
H	4.926067	1.226944	2.01492		
C	2.302619	2.185782	1.883317		
H	1.319948	2.34557	2.334373		
H	2.986253	2.951006	2.259989		
H	2.216247	2.320041	0.802778		

AM2-TB-TS-TL

Atom	X	Y	Z	Electronic Energy (EE)	-727.5398095
O	-0.618168	1.168982	-0.021056	Zero-point Energy Correction	0.289065
C	0.397162	-0.953326	0.206795	Thermal Correction to Energy	0.307171
C	-0.75711	-0.035496	0.123445	Thermal Correction to Enthalpy	0.308115
C	1.748516	-0.35662	0.432037	Thermal Correction to Free Energy	0.241604
H	0.225164	-1.951406	0.596195		
H	2.532829	-1.005489	0.024062		
H	1.792554	0.614095	-0.069263		
N	-1.984976	-0.626758	0.225353		
H	-2.066979	-1.627031	0.123965		
H	-2.777152	-0.074812	-0.071482		
O	1.908369	-0.228408	1.836804		
O	-1.895161	-0.512412	-3.067507		
C	-0.541443	-2.302672	-2.280594		
C	-1.79273	-1.695395	-2.794979		
C	0.529411	-1.525565	-1.982546		
H	-0.519197	-3.374692	-2.109427		
H	1.485938	-1.983344	-1.759354		
H	0.520587	-0.479172	-2.273063		
N	-2.844605	-2.562335	-2.906074		
H	-2.701854	-3.558579	-2.854445		
H	-3.675962	-2.232432	-3.374038		
C	2.86447	0.746049	2.29315		
C	2.987418	0.466916	3.785358		
H	3.366739	-0.543794	3.952324		
H	3.669439	1.179973	4.254186		
H	2.009274	0.553049	4.263453		
C	4.21424	0.548313	1.604203		
H	4.553894	-0.484765	1.717912		
H	4.166602	0.787503	0.538861		
H	4.960307	1.205844	2.056696		
C	2.328397	2.157999	2.063236		
H	1.356896	2.272581	2.548434		
H	3.019704	2.892443	2.484692		
H	2.20678	2.385158	1.0023		

AM2-TL-TS-TL

Atom	X	Y	Z	Electronic Energy (EE)	-765.4356315
O	-0.577402	0.903968	0.552169	Zero-point Energy Correction	0.281837
C	0.34536	-1.25796	0.321734	Thermal Correction to Energy	0.29928
C	-0.769552	-0.294145	0.407252	Thermal Correction to Enthalpy	0.300224
C	1.711925	-0.78509	0.687522	Thermal Correction to Free Energy	0.233661
H	0.107496	-2.305038	0.483836		
H	2.467539	-1.492232	0.333637		

H	1.903813	0.183249	0.217916
N	-2.026217	-0.834019	0.33818
H	-2.135652	-1.76585	-0.034539
H	-2.771584	-0.185534	0.124921
O	-1.895333	-0.127152	-2.823607
C	-0.571459	-2.058211	-2.407083
C	-1.801702	-1.342362	-2.817772
C	0.495295	-1.374099	-1.919191
H	-0.559748	-3.142103	-2.469422
H	1.444363	-1.87772	-1.778089
H	0.5041	-0.290472	-1.988189
N	-2.850824	-2.154691	-3.153121
H	-2.706228	-3.137067	-3.325709
H	-3.661601	-1.722431	-3.571693
C	1.855966	-0.625131	2.219679
H	1.105044	0.08966	2.56442
H	1.653686	-1.584032	2.705073
C	3.236424	-0.144374	2.587787
C	4.237672	-1.044546	2.955117
C	3.551085	1.214848	2.517153
C	5.523348	-0.599458	3.249929
H	4.005401	-2.103499	3.016635
C	4.834201	1.663548	2.810733
H	2.778866	1.923471	2.232823
C	5.825102	0.756701	3.178214
H	6.287899	-1.311633	3.539263
H	5.060693	2.722352	2.756187
H	6.824599	1.105839	3.410151

AM3-AI-TS-IP

Atom	X	Y	Z		
O	-1.752408	0.760246	1.649095	Electronic Energy (EE)	-952.6099074
C	-0.43075	-1.258476	1.65984	Zero-point Energy Correction	0.340385
C	-1.732711	-0.465421	1.597536	Thermal Correction to Energy	0.363036
C	0.572556	-0.635454	2.633543	Thermal Correction to Enthalpy	0.36398
H	-0.657557	-2.289226	1.946589	Thermal Correction to Free Energy	0.286988
H	1.572187	-1.003825	2.386139		
H	0.576546	0.447806	2.485531		
N	-2.850542	-1.19921	1.440949		
H	-2.812674	-2.204943	1.36281		
H	-3.734736	-0.732414	1.288849		
O	-1.766422	-0.079201	-1.563537		
C	-0.659537	-2.018919	-0.755634		
C	-1.680638	-1.309671	-1.545747		
C	0.187352	-1.289839	0.23421		

H	-0.865693	-3.065972	-0.55589
H	1.151023	-1.800084	0.318401
H	0.373342	-0.265381	-0.100039
N	-2.516041	-2.111076	-2.255491
H	-2.233326	-3.069925	-2.436511
H	-3.12026	-1.667744	-2.93468
O	-0.872859	-4.627329	-2.999791
C	1.1662	-3.67711	-2.231083
C	0.295106	-4.8009	-2.629702
C	0.759117	-2.400603	-2.456949
H	2.075477	-3.899065	-1.682328
H	1.409125	-1.565432	-2.220367
H	-0.037204	-2.220744	-3.170651
N	0.840885	-6.028856	-2.537987
H	1.807614	-6.156102	-2.276964
H	0.299782	-6.836325	-2.814954
C	0.318196	-0.946494	4.130204
C	0.493987	-2.441758	4.442277
H	-0.303166	-3.012924	3.963478
H	1.457756	-2.806979	4.080754
H	0.433603	-2.615448	5.518636
C	-1.051818	-0.457144	4.62441
H	-1.195783	0.602723	4.407974
H	-1.842445	-1.03066	4.135087
H	-1.140866	-0.615009	5.701354
C	1.353352	-0.21442	4.880834
N	2.164422	0.353918	5.46647

AM3-AI-TS-TL

Atom	X	Y	Z		
O	-1.789969	0.776571	1.787758	Electronic Energy (EE)	-952.5817824
C	-0.464977	-1.234009	1.829765	Zero-point Energy Correction	0.340788
C	-1.766544	-0.434969	1.72353	Thermal Correction to Energy	0.363566
C	0.530608	-0.548524	2.769088	Thermal Correction to Enthalpy	0.36451
H	-0.69393	-2.245106	2.182244	Thermal Correction to Free Energy	0.287071
H	1.537551	-0.913435	2.547509		
H	0.51489	0.523703	2.55824		
N	-2.893345	-1.181974	1.556165		
H	-2.830662	-2.162491	1.328068		
H	-3.73343	-0.696702	1.273112		
O	-1.800203	-0.419114	-1.546651		
C	-0.651492	-2.214507	-0.509384		
C	-1.722592	-1.618125	-1.332667		
C	0.152241	-1.352666	0.407632		
H	-0.777318	-3.260403	-0.240465		

H	1.151201	-1.783618	0.52384
H	0.256367	-0.351265	-0.019335
N	-2.620725	-2.520175	-1.833771
H	-2.387233	-3.504269	-1.839983
H	-3.262624	-2.189817	-2.539918
O	-0.695684	-5.005601	-2.541831
C	0.208652	-2.934133	-3.259833
C	-0.464402	-4.233976	-3.467557
C	0.854695	-2.683187	-2.090796
H	0.089461	-2.16187	-4.012509
H	1.389048	-1.748598	-1.963847
H	1.129596	-3.513029	-1.449241
N	-0.858614	-4.492884	-4.74039
H	-0.572183	-3.907037	-5.5084
H	-1.2725	-5.389875	-4.946099
C	0.27525	-0.775759	4.280828
C	0.469987	-2.249418	4.676474
H	-0.316071	-2.861989	4.231148
H	1.440598	-2.622457	4.342441
H	0.409441	-2.36516	5.760288
C	-1.107384	-0.2831	4.73632
H	-1.269968	0.757161	4.452819
H	-1.887423	-0.894133	4.275491
H	-1.201807	-0.37751	5.820003
C	1.298154	0.011491	4.994582
N	2.101876	0.616965	5.551012

AM3-C2-IP-TS-IP

Atom	X	Y	Z
O	-1.005727	1.159367	1.866155
C	0.876391	-0.333634	2.086324
C	-0.576841	0.009391	1.78735
C	1.800012	0.593814	1.293526
H	1.053847	-1.365324	1.760148
H	2.831753	0.416788	1.60806
H	1.555649	1.635245	1.518401
O	2.461617	-0.888155	3.820681
C	1.160818	-0.322206	3.616951
C	0.127566	-1.162713	4.362279
C	1.233715	1.079974	4.202838
H	-0.86466	-0.706351	4.316473
H	0.065189	-2.169415	3.936692
H	0.418326	-1.246224	5.411966
H	0.305185	1.623926	4.024059
H	2.060989	1.642246	3.765583

Electronic Energy (EE)	-935.5976484
Zero-point Energy Correction	0.344571
Thermal Correction to Energy	0.367259
Thermal Correction to Enthalpy	0.368204
Thermal Correction to Free Energy	0.291186

H	1.398953	1.010591	5.280851
H	2.443476	-1.807792	3.527681
O	4.45671	-0.173678	-0.767427
C	2.588009	1.272526	-0.973059
C	4.020593	0.948829	-1.04574
C	1.677953	0.359351	-0.223154
H	2.320997	2.32329	-1.02751
H	0.644378	0.537173	-0.53786
H	1.925271	-0.681799	-0.449695
N	4.832677	1.954012	-1.448352
H	5.80243	1.750054	-1.647329
H	4.452601	2.836051	-1.759052
N	-1.361931	-1.020248	1.421859
H	-1.008388	-1.964078	1.378869
H	-2.344534	-0.859038	1.243976
O	4.549652	-0.01019	-4.166669
C	2.779142	1.566693	-3.927629
C	4.096098	1.108651	-4.417774
C	2.038058	0.790026	-3.096826
H	2.451921	2.563244	-4.206592
H	1.020837	1.074972	-2.853777
H	2.321793	-0.245351	-2.932901
N	4.775392	2.005784	-5.165275
H	4.389867	2.913102	-5.379915
H	5.672838	1.752426	-5.554163

AM3-CM-TS-IP

Atom	X	Y	Z		
O	-1.612731	0.959582	1.387178	Electronic Energy (EE)	-1166.595774
C	-0.156943	-0.948745	1.540093	Zero-point Energy Correction	0.427287
C	-1.510845	-0.251885	1.560095	Thermal Correction to Energy	0.453782
C	0.850408	-0.114756	2.313494	Thermal Correction to Enthalpy	0.454726
H	-0.244898	-1.927666	2.021355	Thermal Correction to Free Energy	0.368657
H	1.834228	-0.593989	2.266797		
H	0.924118	0.879895	1.863958		
N	-2.576072	-1.049213	1.751495		
H	-2.471964	-2.044463	1.88278		
H	-3.507523	-0.658088	1.704171		
O	0.391362	-0.050876	3.653661		
O	-2.041623	-0.537913	-1.532998		
C	-0.533322	-2.145048	-0.646851		
C	-1.757162	-1.724436	-1.350328		
C	0.308913	-1.148391	0.079137		
H	-0.539076	-3.160255	-0.262705		
H	1.341881	-1.508616	0.110308		

H	0.298968	-0.186661	-0.440854
N	-2.546556	-2.740017	-1.785629
H	-2.142416	-3.668037	-1.871003
H	-3.306099	-2.503081	-2.410082
O	-0.64725	-5.098097	-2.431921
C	1.303976	-3.770571	-2.140664
C	0.570722	-5.050071	-2.218944
C	0.677698	-2.614815	-2.483811
H	2.302971	-3.774638	-1.7176
H	1.218478	-1.674831	-2.479606
H	-0.228272	-2.66095	-3.07815
N	1.30511	-6.160463	-2.01518
H	2.302961	-6.111945	-1.871516
H	0.862655	-7.067822	-2.064517
C	0.918778	0.993426	4.481661
C	2.445624	1.043184	4.381242
H	2.762461	1.34318	3.379654
H	2.844694	1.771915	5.088662
H	2.873791	0.064346	4.613351
C	0.294393	2.331413	4.0741
H	-0.793931	2.274267	4.150889
H	0.649372	3.132172	4.725822
H	0.562463	2.593412	3.048202
C	0.504253	0.645131	5.910488
C	0.85216	1.504684	6.959127
C	-0.218285	-0.510051	6.210554
C	0.490499	1.215699	8.269914
H	1.412152	2.411184	6.754501
C	-0.582614	-0.799764	7.525583
H	-0.499877	-1.187284	5.415205
C	-0.230149	0.058704	8.560002
H	0.771918	1.895655	9.066494
H	-1.144855	-1.702893	7.737203
H	-0.51308	-0.167878	9.581832

AM3-CM-TS-TL

Atom	X	Y	Z		
O	-1.59409	1.0943	1.43786	Electronic Energy (EE)	-1166.56956
C	-0.189867	-0.85295	1.510501	Zero-point Energy Correction	0.427555
C	-1.529501	-0.11653	1.511599	Thermal Correction to Energy	0.454374
C	0.826332	-0.037101	2.291752	Thermal Correction to Enthalpy	0.455318
H	-0.304199	-1.824232	2.00333	Thermal Correction to Free Energy	0.366988
H	1.821389	-0.487586	2.189907		
H	0.853289	0.976251	1.880302		
N	-2.629417	-0.908674	1.594901		

H	-2.557571	-1.911942	1.536893
H	-3.531431	-0.4884	1.423946
O	0.418279	-0.046306	3.644013
O	-1.972612	-0.519588	-1.67168
C	-0.529898	-2.101396	-0.657516
C	-1.725065	-1.690398	-1.427548
C	0.288185	-1.076335	0.055664
H	-0.562636	-3.098196	-0.227777
H	1.329233	-1.413696	0.098973
H	0.252165	-0.127833	-0.486905
N	-2.519399	-2.726786	-1.833578
H	-2.108469	-3.654431	-1.896236
H	-3.246428	-2.499151	-2.496952
O	-0.680999	-5.118959	-2.177461
C	1.32259	-3.857553	-2.01331
C	0.538853	-5.11107	-2.029077
C	0.732172	-2.702295	-2.418305
H	2.320854	-3.870757	-1.588405
H	1.295784	-1.777049	-2.459492
H	-0.1662	-2.758519	-3.022368
N	1.243028	-6.251836	-1.829015
H	2.249846	-6.252512	-1.805761
H	0.759408	-7.136457	-1.872739
C	0.895625	1.008554	4.483449
C	2.415167	1.153576	4.350356
H	2.691321	1.493022	3.349176
H	2.791352	1.888392	5.063558
H	2.90557	0.197928	4.552152
C	0.179603	2.314634	4.126006
H	-0.899518	2.181804	4.217564
H	0.494204	3.118235	4.794885
H	0.400984	2.621087	3.102058
C	0.538474	0.601166	5.911908
C	0.877759	1.439794	6.978626
C	-0.130966	-0.589701	6.190075
C	0.561931	1.095484	8.287208
H	1.393209	2.375837	6.790713
C	-0.448915	-0.935297	7.502332
H	-0.405323	-1.243338	5.373517
C	-0.103943	-0.097626	8.555538
H	0.834711	1.760875	9.098485
H	-0.971111	-1.865272	7.698068
H	-0.352298	-0.367527	9.575458

AM3-IP-TS-IP

Atom	X	Y	Z	Electronic Energy (EE)	-935.5966786
O	-1.766327	0.697204	1.636717	Zero-point Energy Correction	0.345427
C	-0.428935	-1.308972	1.653098	Thermal Correction to Energy	0.367545
C	-1.738018	-0.529785	1.623773	Thermal Correction to Enthalpy	0.368489
C	0.573131	-0.716491	2.645643	Thermal Correction to Free Energy	0.292941
H	-0.641482	-2.351005	1.908224		
H	1.554275	-1.155106	2.438573		
H	0.65481	0.361043	2.473288		
N	-2.856162	-1.275867	1.543835		
H	-2.814881	-2.283697	1.512921		
H	-3.752866	-0.821134	1.434027		
O	-1.854223	-0.143563	-1.546247		
C	-0.66068	-2.042905	-0.76383		
C	-1.721359	-1.369966	-1.532261		
C	0.173247	-1.29313	0.221763		
H	-0.822394	-3.099355	-0.571971		
H	1.157031	-1.767109	0.285458		
H	0.315607	-0.258286	-0.101174		
N	-2.538106	-2.20086	-2.230809		
H	-2.220302	-3.146706	-2.42118		
H	-3.167113	-1.778519	-2.900784		
O	-0.800423	-4.638604	-3.03444		
C	1.207046	-3.60946	-2.284315		
C	0.378401	-4.765649	-2.680151		
C	0.742076	-2.349628	-2.491809		
H	2.133164	-3.797514	-1.7514		
H	1.359383	-1.489372	-2.257534		
H	-0.072647	-2.198256	-3.191396		
N	0.976051	-5.970356	-2.604668		
H	1.949475	-6.058952	-2.35279		
H	0.464838	-6.798176	-2.878054		
C	0.287028	-0.955314	4.133474		
C	0.217162	-2.443278	4.468299		
H	-0.679147	-2.907039	4.050128		
H	1.097215	-2.965883	4.083441		
H	0.183305	-2.574864	5.553997		
C	-0.964754	-0.237037	4.629541		
H	-0.917456	0.828103	4.39249		
H	-1.872341	-0.656154	4.188481		
H	-1.043777	-0.351373	5.715339		
O	1.436691	-0.385537	4.786011		
H	1.317365	-0.47897	5.739022		

AM3-TB-TS-IP

Atom	X	Y	Z	Electronic Energy (EE)	-974.8866621

O	-1.613796	0.795471	1.613081	Zero-point Energy Correction	0.373344
C	-0.108465	-1.083443	1.561919	Thermal Correction to Energy	0.39692
C	-1.477727	-0.423683	1.667482	Thermal Correction to Enthalpy	0.397864
C	0.904334	-0.287914	2.36813	Thermal Correction to Free Energy	0.318725
H	-0.163972	-2.097768	1.969288		
H	1.898991	-0.729878	2.239345		
H	0.932035	0.742663	2.001383		
N	-2.52167	-1.262167	1.793705		
H	-2.392213	-2.261859	1.842304		
H	-3.462294	-0.890848	1.802663		
O	0.505282	-0.346082	3.726509		
O	-1.998316	-0.346965	-1.454186		
C	-0.550833	-2.077665	-0.714872		
C	-1.759098	-1.554895	-1.376577		
C	0.325976	-1.167186	0.079774		
H	-0.597863	-3.116868	-0.404916		
H	1.351099	-1.549863	0.062852		
H	0.328775	-0.164672	-0.356481		
N	-2.58606	-2.498281	-1.89462		
H	-2.220068	-3.432031	-2.056571		
H	-3.338837	-2.181044	-2.490782		
O	-0.807901	-4.878323	-2.707771		
C	1.209697	-3.679558	-2.325261		
C	0.410301	-4.909969	-2.491927		
C	0.6421	-2.471822	-2.581515		
H	2.207736	-3.766159	-1.908769		
H	1.228957	-1.562581	-2.512044		
H	-0.265038	-2.430582	-3.174468		
N	1.082802	-6.069751	-2.366554		
H	2.080393	-6.086139	-2.213918		
H	0.591345	-6.94603	-2.475867		
C	1.034998	0.66424	4.610106		
C	0.654926	0.17745	6.001973		
H	1.12897	-0.784866	6.211631		
H	0.9769	0.89721	6.75793		
H	-0.428893	0.056747	6.076493		
C	2.552978	0.75986	4.476926		
H	3.011884	-0.223233	4.614952		
H	2.850717	1.153477	3.501931		
H	2.945535	1.433044	5.242805		
C	0.369476	2.008929	4.323318		
H	0.606949	2.378418	3.323192		
H	-0.715981	1.918239	4.413307		
H	0.716173	2.753846	5.044267		

AM3-TB-TS-TL

Atom	X	Y	Z		
O	-1.562324	0.938439	1.684432	Zero-point Energy Correction	0.374037
C	-0.140353	-0.993071	1.531642	Thermal Correction to Energy	0.397733
C	-1.483685	-0.273344	1.646024	Thermal Correction to Enthalpy	0.398678
C	0.90106	-0.241028	2.343148	Thermal Correction to Free Energy	0.318708
H	-0.231557	-2.003403	1.944283		
H	1.894443	-0.669235	2.15661		
H	0.904385	0.806462	2.02626		
N	-2.573462	-1.082828	1.690288		
H	-2.49244	-2.076948	1.547358		
H	-3.484842	-0.661281	1.585483		
O	0.550674	-0.37753	3.703164		
O	-2.047389	-0.397073	-1.547634		
C	-0.568018	-2.052734	-0.718779		
C	-1.792535	-1.582876	-1.404185		
C	0.279053	-1.089543	0.044912		
H	-0.585814	-3.081882	-0.372582		
H	1.320841	-1.42643	0.018384		
H	0.222024	-0.098679	-0.413654		
N	-2.606225	-2.585932	-1.854134		
H	-2.200262	-3.505454	-2.005023		
H	-3.359845	-2.309426	-2.467234		
O	-0.781923	-4.94137	-2.459805		
C	1.227787	-3.691925	-2.279442		
C	0.442498	-4.942266	-2.355317		
C	0.621607	-2.510991	-2.57118		
H	2.2423	-3.734714	-1.897312		
H	1.182699	-1.583345	-2.564296		
H	-0.301014	-2.524045	-3.140133		
N	1.151825	-6.09373	-2.25936		
H	2.158727	-6.094993	-2.280006		
H	0.665384	-6.973178	-2.349412		
C	1.060711	0.607788	4.615716		
C	0.714559	0.051217	5.990809		
H	1.211736	-0.908916	6.147369		
H	1.029313	0.742242	6.776162		
H	-0.364099	-0.100684	6.07092		
C	2.575342	0.751352	4.469121		
H	3.06176	-0.223455	4.563279		
H	2.849916	1.188994	3.506052		
H	2.963965	1.405845	5.252916		
C	0.355641	1.945806	4.397985		
H	0.567356	2.368076	3.413836		
H	-0.724998	1.8166	4.48298		
H	0.685486	2.666959	5.150523		

Electronic Energy (EE) -974.8599285

AM3-TL-TS-TL

Atom	X	Y	Z		
O	-1.689921	0.684443	2.152387	Electronic Energy (EE)	-1012.745819
C	-0.34677	-1.279366	1.807872	Zero-point Energy Correction	0.366913
C	-1.661496	-0.506879	1.917324	Thermal Correction to Energy	0.389891
C	0.679929	-0.715127	2.788995	Thermal Correction to Enthalpy	0.390836
H	-0.533541	-2.333595	2.045873	Thermal Correction to Free Energy	0.310459
H	1.6366	-1.224745	2.635378		
H	0.833856	0.344436	2.566231		
N	-2.792895	-1.248803	1.757684		
H	-2.739729	-2.182391	1.379365		
H	-3.655485	-0.744256	1.606302		
O	-2.022863	-0.212871	-1.236515		
C	-0.615402	-2.004528	-0.610175		
C	-1.810416	-1.412531	-1.24562		
C	0.183034	-1.186701	0.351816		
H	-0.614633	-3.081821	-0.465766		
H	1.217812	-1.541236	0.365758		
H	0.183511	-0.139154	0.03944		
N	-2.680311	-2.31303	-1.813264		
H	-2.327095	-3.228565	-2.054147		
H	-3.330237	-1.917144	-2.479754		
O	-1.136545	-1.512715	-4.544559		
C	0.264785	-2.966052	-3.290345		
C	-0.717884	-2.640625	-4.350753		
C	0.695343	-2.007727	-2.4279		
H	0.605513	-3.992947	-3.197528		
H	1.533594	-2.212788	-1.772784		
H	0.450133	-0.968359	-2.62516		
N	-1.157577	-3.714922	-5.075168		
H	-0.669867	-4.596212	-5.038095		
H	-1.715526	-3.527091	-5.895491		
C	0.253277	-0.872824	4.253821		
H	-0.708068	-0.373248	4.402776		
H	0.111104	-1.934376	4.480112		
C	1.275184	-0.28578	5.194814		
C	1.316327	1.093508	5.415111		
C	2.225466	-1.090427	5.824528		
C	2.280582	1.653184	6.245901		
H	0.581104	1.729045	4.93018		
C	3.192933	-0.534316	6.657519		
H	2.203635	-2.164249	5.664818		
C	3.223272	0.83986	6.87025		
H	2.295146	2.724879	6.409237		

H	3.920608	-1.175417	7.14237
H	3.973443	1.274806	7.520472

MA-C2-IP-TS-IP

Atom	X	Y	Z	Electronic Energy (EE)	-500.1104331
O	-0.553463	1.398819	-1.046047	Zero-point Energy Correction	0.191872
O	-1.291894	0.642597	0.928672	Thermal Correction to Energy	0.204079
C	0.937428	0.125109	0.217985	Thermal Correction to Enthalpy	0.205023
C	-0.404346	0.727964	0.104266	Thermal Correction to Free Energy	0.153647
C	1.460498	-0.111836	1.459739		
H	1.590408	0.270283	-0.63593		
H	2.497768	-0.399621	1.579513		
H	0.834841	-0.075937	2.344219		
C	-1.847406	1.965147	-1.285189		
H	-2.08747	2.702202	-0.518253		
H	-1.783877	2.443266	-2.259748		
H	-2.607907	1.182823	-1.296581		
O	1.582945	-2.356743	-0.947813		
C	0.338488	-1.852986	-0.674855		
C	-0.505887	-1.535853	-1.868136		
C	-0.285044	-2.550283	0.483789		
H	-1.457288	-1.091938	-1.569473		
H	0.005401	-0.853353	-2.553122		
H	-0.723095	-2.465521	-2.412561		
H	-1.180304	-2.023361	0.817673		
H	0.421951	-2.619604	1.314205		
H	-0.572379	-3.568945	0.192046		
H	1.917769	-1.98633	-1.775175		

MA-C3-AI-TS-IP

Atom	X	Y	Z	Electronic Energy (EE)	-517.1299883
O	-1.567633	-0.482064	-0.51985	Zero-point Energy Correction	0.186865
O	-0.529171	1.330075	0.297137	Thermal Correction to Energy	0.199806
C	0.730001	-0.636905	-0.199341	Thermal Correction to Enthalpy	0.20075
C	-0.481862	0.183368	-0.102224	Thermal Correction to Free Energy	0.146301
C	1.92761	-0.150126	0.225452		
H	0.618849	-1.645961	-0.577451		
H	2.839642	-0.691005	-0.001805		
H	2.019592	0.905087	0.464104		
C	-2.804913	0.240095	-0.477449		
H	-2.749388	1.123456	-1.114563		
H	-3.559461	-0.447789	-0.850905		
H	-3.035425	0.535472	0.546755		
C	2.098827	-0.737645	2.367674		

C	0.884654	-0.118797	2.993895
H	0.859606	0.963158	2.857964
H	-0.01745	-0.561113	2.565076
H	0.884004	-0.332544	4.069821
C	2.163338	-2.234717	2.284445
H	1.238105	-2.618342	1.849875
H	3.012866	-2.575048	1.690774
H	2.260084	-2.650772	3.294586
C	3.333036	-0.064759	2.583371
N	4.336714	0.495302	2.711732

MA-C3-AI-TS-TL

Atom	X	Y	Z		
				Electronic Energy (EE)	-517.1233463
O	-1.579976	-0.472551	-0.523185	Zero-point Energy Correction	0.187214
O	-0.522309	1.316155	0.324216	Thermal Correction to Energy	0.200236
C	0.717435	-0.652271	-0.202234	Thermal Correction to Enthalpy	0.20118
C	-0.488811	0.181854	-0.093247	Thermal Correction to Free Energy	0.146463
C	1.917868	-0.171702	0.220397		
H	0.597868	-1.655383	-0.592598		
H	2.830757	-0.708059	-0.012262		
H	2.00737	0.883944	0.455842		
C	-2.801976	0.26746	-0.477539		
H	-2.735451	1.153161	-1.110687		
H	-3.570128	-0.406169	-0.849706		
H	-3.0269	0.571874	0.545439		
C	2.10414	-0.737549	2.366581		
C	0.872107	-0.139477	2.980321		
H	0.805145	0.934342	2.803562		
H	-0.017071	-0.623173	2.569809		
H	0.879536	-0.313388	4.063185		
C	2.195254	-2.235391	2.299828		
H	1.289777	-2.641319	1.844038		
H	3.065495	-2.568694	1.733018		
H	2.273868	-2.645729	3.313748		
C	3.326431	-0.042004	2.586706		
N	4.321198	0.532496	2.713839		

MA-C3-CM-TS-IP

Atom	X	Y	Z		
				Electronic Energy (EE)	-731.1030214
O	-1.522674	0.564616	-0.248702	Zero-point Energy Correction	0.274737
O	-0.792783	1.804663	-1.968098	Thermal Correction to Energy	0.290981
C	0.51331	-0.03801	-1.193315	Thermal Correction to Enthalpy	0.291925
C	-0.641053	0.876254	-1.203361	Thermal Correction to Free Energy	0.229254
C	1.539178	0.146961	-2.066561		

H	0.534929	-0.806006	-0.42969
H	2.34671	-0.571901	-2.11959
H	1.42145	0.825611	-2.901949
O	2.601261	1.611449	-1.15067
C	3.083112	1.391702	0.157053
C	4.244689	2.407668	0.208402
C	2.003751	1.682643	1.196795
C	3.630341	-0.027479	0.341034
C	4.29983	3.437277	1.147715
C	5.277974	2.29501	-0.729741
C	5.379354	4.320025	1.165534
C	6.350383	3.178233	-0.714837
C	6.406273	4.194587	0.237102
H	1.217766	0.928893	1.116812
H	2.408456	1.638825	2.210768
H	1.558419	2.666256	1.031588
H	2.812316	-0.752148	0.329274
H	4.140462	-0.104324	1.304328
H	4.338049	-0.282651	-0.44969
H	3.511519	3.559589	1.879693
H	5.239358	1.513626	-1.480203
H	5.411082	5.109104	1.908729
H	7.142327	3.075191	-1.448357
H	7.242806	4.88419	0.251288
C	-2.686852	1.398775	-0.168651
H	-3.278928	0.998158	0.650252
H	-2.397419	2.429167	0.04
H	-3.248479	1.351824	-1.102125

MA-C3-CM-TS-TL

Atom	X	Y	Z		
O	-1.512019	0.559114	-0.231475	Zero-point Energy Correction	0.274968
O	-0.883392	1.690595	-2.064594	Thermal Correction to Energy	0.291291
C	0.514453	-0.040491	-1.198359	Thermal Correction to Enthalpy	0.292235
C	-0.67156	0.835429	-1.241343	Thermal Correction to Free Energy	0.229141
C	1.549302	0.16927	-2.055187		
H	0.544771	-0.798709	-0.425548		
H	2.370058	-0.533899	-2.106688		
H	1.419345	0.848737	-2.887415		
O	2.60437	1.618006	-1.149972		
C	3.075012	1.421134	0.159081		
C	4.249876	2.421182	0.210174		
C	1.990081	1.739901	1.186591		
C	3.615073	0.001464	0.375677		
C	4.358995	3.404032	1.192311		

Electronic Energy (EE): -731.0967503

C	5.247531	2.331778	-0.766411
C	5.456214	4.263433	1.212936
C	6.337341	3.191919	-0.749236
C	6.447655	4.160743	0.245423
H	1.179773	1.014971	1.089422
H	2.372972	1.676775	2.208034
H	1.579245	2.736743	1.015024
H	2.79772	-0.724188	0.366652
H	4.117031	-0.06311	1.343778
H	4.332814	-0.265318	-0.402237
H	3.595844	3.510734	1.952456
H	5.158044	1.590302	-1.551505
H	5.529026	5.017599	1.988257
H	7.099782	3.109667	-1.515392
H	7.297525	4.83316	0.260943
C	-2.698846	1.354503	-0.182463
H	-3.25501	1.003879	0.683613
H	-2.445855	2.409462	-0.070229
H	-3.286251	1.218964	-1.091492

MA-C3-IP-TS-IP

Atom	X	Y	Z		
O	-0.755525	1.595718	-0.79489	Electronic Energy (EE)	-500.1110999
O	-1.080898	0.406913	1.076711	Zero-point Energy Correction	0.19316
C	0.887172	0.043949	-0.236805	Thermal Correction to Energy	0.205315
C	-0.396581	0.6685	0.106514	Thermal Correction to Enthalpy	0.20626
C	1.406701	-0.909705	0.547278	Thermal Correction to Free Energy	0.153416
H	1.370056	0.375675	-1.148271		
H	2.339955	-1.400252	0.302019		
H	0.908251	-1.211579	1.462585		
C	-1.997931	2.266371	-0.552095		
H	-1.961207	2.800782	0.397829		
H	-2.119146	2.967615	-1.374245		
H	-2.820142	1.549905	-0.542158		
O	1.214243	-3.883933	0.890689		
C	0.788775	-3.03367	-0.091439		
C	1.595475	-2.97459	-1.339093		
C	-0.683174	-2.848335	-0.111689		
H	1.284331	-2.125025	-1.950074		
H	2.661793	-2.864925	-1.112427		
H	1.467185	-3.888634	-1.937935		
H	-0.946199	-1.974567	-0.712172		
H	-1.073113	-2.713833	0.900576		
H	-1.186095	-3.72269	-0.552213		
H	2.165513	-4.032286	0.810762		

Electronic Energy (EE) row etc. appear beside the table.

MA-C3-TB-TS-IP

Atom	X	Y	Z		
O	-0.337355	0.979324	-0.795336	Electronic Energy (EE)	-539.393542
O	1.785821	0.542496	-1.365056	Zero-point Energy Correction	0.220641
C	0.748604	-0.688293	0.401745	Thermal Correction to Energy	0.234028
C	0.818798	0.321136	-0.666737	Thermal Correction to Enthalpy	0.234972
C	1.851823	-1.392829	0.779	Thermal Correction to Free Energy	0.179925
H	-0.209724	-0.830575	0.886502		
H	1.741964	-2.264604	1.408664		
H	2.774705	-1.299865	0.218167		
O	2.434682	-0.433352	2.454069		
C	2.974505	0.862766	2.376568		
C	3.650896	1.004926	3.758594		
C	1.887257	1.927874	2.235073		
C	4.023836	0.979285	1.274187		
H	4.421588	0.2426	3.887262		
H	2.913646	0.909395	4.558277		
H	4.114185	1.992744	3.819976		
H	1.1053	1.770815	2.982058		
H	2.311931	2.925078	2.37753		
H	1.433072	1.897579	1.241625		
H	4.78005	0.197815	1.386221		
H	4.518339	1.952559	1.328304		
H	3.56133	0.887115	0.289063		
C	-0.378514	1.988324	-1.813453		
H	-0.191187	1.544706	-2.791801		
H	-1.381372	2.405686	-1.772383		
H	0.363322	2.760792	-1.607024		

MA-C3-TB-TS-TL

Atom	X	Y	Z		
O	-0.365995	0.962129	-0.822465	Electronic Energy (EE)	-539.3889476
O	1.789597	0.595071	-1.318197	Zero-point Energy Correction	0.221249
C	0.735548	-0.671549	0.41036	Thermal Correction to Energy	0.234598
C	0.810281	0.341001	-0.656183	Thermal Correction to Enthalpy	0.235542
C	1.841952	-1.371581	0.785965	Thermal Correction to Free Energy	0.180829
H	-0.221791	-0.81533	0.895371		
H	1.741735	-2.244928	1.413847		
H	2.757943	-1.272926	0.215399		
O	2.437871	-0.430633	2.449564		
C	2.983267	0.857739	2.37887		
C	3.6601	0.988882	3.761711		
C	1.900533	1.930584	2.245075		

C	4.033941	0.979449	1.276243
H	4.422713	0.218518	3.886578
H	2.921664	0.891393	4.559346
H	4.132378	1.97174	3.832592
H	1.118066	1.768495	2.989497
H	2.326125	2.926744	2.392425
H	1.445958	1.906297	1.252222
H	4.78476	0.192403	1.383188
H	4.53654	1.948256	1.336947
H	3.573749	0.900272	0.288862
C	-0.396918	1.965233	-1.840573
H	-0.153625	1.531778	-2.811309
H	-1.41246	2.353522	-1.842348
H	0.313569	2.760938	-1.611891

MA-C3-TL-TS-TL

Atom	X	Y	Z		
O	-1.461958	-0.578596	-0.467506	Electronic Energy (EE)	-577.2874864
O	-0.534665	1.376223	0.129637	Zero-point Energy Correction	0.213381
C	0.839595	-0.554304	-0.118693	Thermal Correction to Energy	0.226222
C	-0.41685	0.199569	-0.131994	Thermal Correction to Enthalpy	0.227166
C	2.006869	0.084334	0.160241	Thermal Correction to Free Energy	0.171322
H	0.784529	-1.625112	-0.269537		
H	2.952604	-0.436325	0.066044		
H	2.037022	1.168148	0.143555		
C	4.658557	2.987145	2.705594		
C	3.460223	2.304186	2.571099		
C	3.429815	0.894486	2.570361		
C	4.65163	0.201959	2.694504		
C	5.846814	0.89066	2.829063		
C	5.857486	2.285626	2.835656		
H	4.662832	4.071141	2.709617		
H	2.527998	2.850376	2.467726		
H	4.645349	-0.883428	2.691117		
H	6.776292	0.342232	2.930878		
H	6.793084	2.821902	2.940991		
C	2.194684	0.187871	2.387514		
H	2.170064	-0.880452	2.564298		
H	1.259977	0.721954	2.50798		
C	-2.730412	0.075202	-0.507335		
H	-3.44976	-0.689721	-0.790708		
H	-2.981094	0.486517	0.471477		
H	-2.724212	0.87993	-1.243818		

Electronic Energy (EE) values are listed alongside the coordinates above.

MA2-AI-TS-IP

Atom	X	Y	Z		
				Electronic Energy (EE)	-823.605051
O	-1.63611	-0.858686	0.479599	Zero-point Energy Correction	0.287932
O	-0.780147	1.13216	-0.113926	Thermal Correction to Energy	0.307425
C	0.675135	-0.675856	0.426787	Thermal Correction to Enthalpy	0.30837
C	-0.618421	-0.02863	0.208919	Thermal Correction to Free Energy	0.238166
C	1.924062	-0.032367	-0.076346		
H	0.644088	-1.751456	0.555887		
H	1.816604	1.054741	-0.046094		
C	-2.949292	-0.284962	0.441946		
H	-3.175378	0.076881	-0.561804		
H	-3.629989	-1.088152	0.714366		
H	-3.024286	0.535235	1.15757		
O	-2.447254	-1.290327	3.588007		
O	-0.681726	-2.622661	3.21229		
C	-0.427747	-0.254384	3.09006		
C	-1.158559	-1.509291	3.303615		
C	0.846495	-0.280154	2.612696		
H	-0.98636	0.669844	3.183736		
H	1.387033	0.651153	2.484082		
H	1.429273	-1.193384	2.670288		
C	-3.266196	-2.45648	3.738674		
H	-2.922934	-3.053267	4.584615		
H	-4.2727	-2.087778	3.920678		
H	-3.239689	-3.05631	2.828051		
H	2.762009	-0.312825	0.568541		
C	2.282433	-0.462434	-1.531931		
C	1.195553	-0.052779	-2.536145		
H	0.280605	-0.612559	-2.327108		
H	0.979459	1.015182	-2.469913		
H	1.513377	-0.288461	-3.553782		
C	2.562738	-1.969574	-1.620773		
H	2.906371	-2.233415	-2.622914		
H	3.322385	-2.270967	-0.896379		
H	1.642312	-2.520544	-1.418495		
C	3.520528	0.254367	-1.878268		
N	4.488917	0.814931	-2.14554		

MA2-AI-TS-TL

Atom	X	Y	Z		
				Electronic Energy (EE)	-823.594409
O	-1.667856	-0.77291	0.469943	Zero-point Energy Correction	0.28842
O	-0.752746	1.184999	-0.150842	Thermal Correction to Energy	0.308071
C	0.648995	-0.649098	0.430684	Thermal Correction to Enthalpy	0.309015
C	-0.626829	0.031271	0.192037	Thermal Correction to Free Energy	0.238168
C	1.912384	-0.035823	-0.076213		

H	0.589764	-1.720837	0.581426
H	1.812761	1.052406	-0.066917
C	-2.958743	-0.161301	0.416395
H	-3.16588	0.207278	-0.589054
H	-3.667229	-0.940598	0.6875
H	-3.018815	0.666215	1.124901
O	-2.437032	-1.361353	3.588961
O	-0.618842	-2.618858	3.196041
C	-0.459249	-0.2447	3.087172
C	-1.139235	-1.532832	3.295867
C	0.815584	-0.226882	2.615089
H	-1.05161	0.657948	3.17954
H	1.329818	0.718742	2.484572
H	1.420503	-1.124594	2.682939
C	-3.197922	-2.561626	3.735127
H	-2.809202	-3.164211	4.556956
H	-4.215791	-2.243974	3.949012
H	-3.165664	-3.145903	2.814169
H	2.745162	-0.30932	0.578792
C	2.279395	-0.489833	-1.522454
C	1.194308	-0.09358	-2.534175
H	0.281901	-0.658672	-2.329217
H	0.964613	0.971084	-2.47083
H	1.518489	-0.327686	-3.549886
C	2.549484	-2.000298	-1.583387
H	2.887083	-2.287712	-2.580827
H	3.313227	-2.294658	-0.860519
H	1.629248	-2.545634	-1.365543
C	3.52425	0.215166	-1.874539
N	4.498268	0.764582	-2.141731

MA2-CM-TS-IP

Atom	X	Y	Z		
O	-1.509012	-0.969368	0.457409	Electronic Energy (EE)	-1037.589268
O	-1.285672	1.219622	-0.002297	Zero-point Energy Correction	0.374492
C	0.640728	-0.095527	0.443121	Thermal Correction to Energy	0.397851
C	-0.785618	0.140242	0.25453	Thermal Correction to Enthalpy	0.398795
C	1.591231	0.965041	0.013106	Thermal Correction to Free Energy	0.317897
H	0.974725	-1.126664	0.425389		
H	1.610417	0.993851	-1.089137		
H	1.251989	1.94821	0.358445		
C	-2.93355	-0.810204	0.428385		
H	-3.255766	-0.47254	-0.557377		
H	-3.344847	-1.793903	0.642488		
H	-3.249452	-0.093292	1.187566		

O	2.870153	0.649505	0.527723
O	-2.177584	-1.822643	3.468268
O	-0.128177	-2.624533	3.032483
C	-0.483942	-0.267457	3.128466
C	-0.872705	-1.680576	3.206029
C	0.751788	0.074782	2.67516
H	-1.257042	0.47206	3.303137
H	1.046126	1.1184	2.652206
H	1.543613	-0.665522	2.641766
C	-2.673255	-3.166695	3.486525
H	-2.201671	-3.733136	4.290531
H	-3.743253	-3.083781	3.661192
H	-2.480021	-3.653798	2.529831
C	3.9717	1.340942	-0.086229
C	3.771452	2.851587	0.018351
H	4.695789	3.377372	-0.226899
H	2.985931	3.213487	-0.648789
H	3.504291	3.10118	1.048261
C	5.186612	0.915885	0.734384
H	6.096036	1.346183	0.310065
H	5.078645	1.261723	1.765722
H	5.281107	-0.172481	0.732949
C	4.140769	0.85324	-1.527079
C	3.909573	-0.494345	-1.822433
C	4.569141	1.697021	-2.552212
C	4.096359	-0.984133	-3.110186
H	3.572254	-1.160855	-1.035709
C	4.762763	1.206291	-3.843214
H	4.756376	2.746414	-2.356355
C	4.525607	-0.133754	-4.127508
H	3.90639	-2.03094	-3.32065
H	5.095855	1.87786	-4.626882
H	4.671521	-0.513789	-5.132404

MA2-CM-TS-TL

Atom	X	Y	Z		
				Electronic Energy (EE)	-1037.581504
O	-1.598721	-0.842467	0.441818	Zero-point Energy Correction	0.375354
O	-1.321719	1.353413	0.039668	Thermal Correction to Energy	0.3987
C	0.574185	-0.029736	0.398702	Thermal Correction to Enthalpy	0.399644
C	-0.849605	0.257747	0.251477	Thermal Correction to Free Energy	0.319226
C	1.550889	1.008144	-0.02947		
H	0.874723	-1.070264	0.36275		
H	1.598213	1.018323	-1.131504		
H	1.211988	2.000747	0.288833		
C	-3.012137	-0.635223	0.459078		

H	-3.351619	-0.231394	-0.495657
H	-3.453773	-1.613705	0.633501
H	-3.288048	0.050924	1.261286
O	2.811174	0.684771	0.520931
O	-2.078117	-1.937188	3.454464
O	-0.011693	-2.635007	2.921727
C	-0.461002	-0.301272	3.106668
C	-0.784976	-1.73508	3.14932
C	0.745412	0.10321	2.630707
H	-1.258648	0.398513	3.326641
H	1.002051	1.156605	2.622953
H	1.562793	-0.603998	2.550562
C	-2.503838	-3.299835	3.446716
H	-1.968199	-3.873634	4.204352
H	-3.567838	-3.280505	3.671735
H	-2.328697	-3.748329	2.46756
C	3.936639	1.344218	-0.07582
C	3.762414	2.861055	-0.00423
H	4.70121	3.367789	-0.234476
H	3.000337	3.226072	-0.696253
H	3.474692	3.134967	1.013576
C	5.121593	0.914031	0.785619
H	6.051793	1.315216	0.378607
H	4.990757	1.279671	1.806894
H	5.192437	-0.175074	0.808615
C	4.133923	0.826756	-1.503094
C	3.912724	-0.52877	-1.765816
C	4.569566	1.64505	-2.544492
C	4.1175	-1.050785	-3.036868
H	3.568003	-1.17186	-0.963208
C	4.78095	1.122143	-3.819358
H	4.746886	2.700343	-2.374141
C	4.554451	-0.225211	-4.070373
H	3.935635	-2.103317	-3.222413
H	5.118916	1.774331	-4.616653
H	4.714151	-0.630424	-5.062803

MA2-IP-TS-IP

Atom	X	Y	Z		
				Electronic Energy (EE)	-806.5925071
O	-1.649073	-0.868063	0.478541	Zero-point Energy Correction	0.293146
O	-0.796766	1.122426	-0.120248	Thermal Correction to Energy	0.312067
C	0.662911	-0.686314	0.411054	Thermal Correction to Enthalpy	0.313012
C	-0.630988	-0.038663	0.201019	Thermal Correction to Free Energy	0.244346
C	1.909805	-0.053555	-0.105945		
H	0.627082	-1.762085	0.540967		

H	1.811364	1.035161	-0.085089
C	-2.960865	-0.291936	0.452122
H	-3.194997	0.072223	-0.548992
H	-3.641308	-1.094003	0.728746
H	-3.029191	0.527407	1.169529
O	-2.447373	-1.28319	3.607233
O	-0.689681	-2.622483	3.219314
C	-0.430243	-0.255581	3.082785
C	-1.161553	-1.506528	3.309657
C	0.839133	-0.28658	2.591592
H	-0.98589	0.67041	3.176564
H	1.380965	0.642626	2.454254
H	1.421106	-1.200241	2.649877
C	-3.266639	-2.4466	3.773931
H	-2.914674	-3.039862	4.618815
H	-4.270189	-2.074944	3.96617
H	-3.252662	-3.051551	2.866403
H	2.753038	-0.333326	0.533609
C	2.27657	-0.475583	-1.544707
O	3.488321	0.247607	-1.803632
H	3.780401	0.032432	-2.69783
C	1.209864	-0.055002	-2.550086
H	0.272319	-0.592018	-2.381339
H	1.017333	1.018175	-2.478964
H	1.548695	-0.283899	-3.565014
C	2.551167	-1.9727	-1.634358
H	2.915411	-2.22309	-2.635114
H	3.309865	-2.266743	-0.904404
H	1.64382	-2.553175	-1.452318

MA2-TB-TS-IP

Atom	X	Y	Z		
O	-1.430008	-1.337675	0.427645	Electronic Energy (EE)	-845.8774385
O	-1.078375	0.585555	-0.682286	Zero-point Energy Correction	0.320996
C	0.753004	-0.558189	0.302462	Thermal Correction to Energy	0.341265
C	-0.645295	-0.367172	-0.060365	Thermal Correction to Enthalpy	0.34221
C	1.773497	0.306582	-0.350823	Thermal Correction to Free Energy	0.270455
H	1.046447	-1.549058	0.629936		
H	1.825765	0.042031	-1.419376		
H	1.46836	1.35752	-0.294537		
C	-2.841021	-1.162391	0.245087		
H	-3.085948	-1.144865	-0.817558		
H	-3.309224	-2.018816	0.724618		
H	-3.170865	-0.235798	0.717204		
O	3.014253	0.076601	0.282284		

O	-2.324891	-1.170756	3.538159
O	-0.296284	-2.127354	3.490365
C	-0.550935	0.148024	2.819631
C	-1.0048	-1.156316	3.315727
C	0.723539	0.300851	2.371386
H	-1.299589	0.922557	2.698244
H	1.063161	1.275999	2.03896
H	1.48361	-0.428421	2.629421
C	-2.878468	-2.42182	3.963582
H	-2.46195	-2.711211	4.929224
H	-3.94957	-2.25566	4.049594
H	-2.672074	-3.197777	3.225378
C	4.058091	1.043751	0.030872
C	3.751687	2.340274	0.777707
H	4.577985	3.044158	0.65014
H	2.845951	2.823704	0.403826
H	3.630767	2.140641	1.845737
C	4.220947	1.294125	-1.465893
H	5.10366	1.914929	-1.636174
H	4.356483	0.349297	-1.999544
H	3.359091	1.817228	-1.887529
C	5.310299	0.391987	0.600343
H	6.165233	1.064791	0.503738
H	5.167908	0.160966	1.659166
H	5.533197	-0.535507	0.066948

MA2-TB-TS-TL

Atom	X	Y	Z		
O	-1.494036	-1.247557	0.400053	Zero-point Energy Correction	0.321442
O	-1.098012	0.687195	-0.677265	Thermal Correction to Energy	0.341908
C	0.70531	-0.514468	0.28922	Thermal Correction to Enthalpy	0.342852
C	-0.688656	-0.28118	-0.073894	Thermal Correction to Free Energy	0.269493
C	1.750682	0.328533	-0.354353		
H	0.97436	-1.512078	0.615554		
H	1.842999	0.028575	-1.411301		
H	1.435542	1.378539	-0.349421		
C	-2.894542	-1.033383	0.217318		
H	-3.138454	-0.981069	-0.844632		
H	-3.387735	-1.887048	0.676402		
H	-3.207523	-0.109758	0.706474		
O	2.961916	0.122475	0.33344		
O	-2.269876	-1.275047	3.55609		
O	-0.197741	-2.136301	3.483855		
C	-0.560253	0.129016	2.836176		
C	-0.947966	-1.203253	3.32343		

Electronic Energy (EE): -845.8687659

C	0.696194	0.335669	2.363192
H	-1.341889	0.873568	2.740557
H	0.995031	1.324593	2.032988
H	1.487616	-0.369154	2.590748
C	-2.750135	-2.554194	3.970265
H	-2.299349	-2.844029	4.92051
H	-3.826712	-2.446421	4.081992
H	-2.518021	-3.310361	3.218678
C	4.037716	1.034741	0.049548
C	3.747177	2.396967	0.679555
H	4.601539	3.063678	0.53913
H	2.87762	2.882063	0.229583
H	3.572182	2.284573	1.752392
C	4.269466	1.164377	-1.45482
H	5.180263	1.739072	-1.637793
H	4.390543	0.177364	-1.908988
H	3.446193	1.682356	-1.952945
C	5.246449	0.394661	0.718958
H	6.128088	1.029773	0.608223
H	5.052065	0.249239	1.783948
H	5.455587	-0.578938	0.270431

MA2-TL-TS-TL

Atom	X	Y	Z	Electronic Energy (EE)	-883.7621123
O	-1.546577	-0.900636	0.442696	Zero-point Energy Correction	0.314774
O	-0.660006	1.025396	-0.307254	Thermal Correction to Energy	0.334548
C	0.769162	-0.706165	0.475721	Thermal Correction to Enthalpy	0.335492
C	-0.515424	-0.091943	0.137912	Thermal Correction to Free Energy	0.262607
C	2.019081	-0.07388	-0.034281		
H	0.745438	-1.775286	0.657279		
H	1.947164	1.013187	0.056189		
C	-2.848654	-0.335032	0.285411		
H	-3.021034	-0.0532	-0.754159		
H	-3.548351	-1.111168	0.58707		
H	-2.963685	0.5435	0.922254		
O	-2.453106	-1.29042	3.538078		
O	-0.607064	-2.551661	3.331779		
C	-0.458876	-0.187615	3.071457		
C	-1.138344	-1.466171	3.32771		
C	0.833589	-0.185398	2.652578		
H	-1.061342	0.71294	3.085669		
H	1.349321	0.754311	2.490484		
H	1.439184	-1.074665	2.788991		
C	-3.211402	-2.486556	3.719759		
H	-2.874892	-3.023458	4.607712		

H	-4.244285	-2.168197	3.84139
H	-3.112217	-3.136134	2.84867
H	2.878276	-0.407737	0.554606
C	2.260683	-0.429528	-1.520677
H	1.397011	-0.100175	-2.104456
H	2.337553	-1.515164	-1.624645
C	3.513673	0.230191	-2.038032
C	3.479041	1.548899	-2.496916
C	4.737667	-0.440024	-2.01916
C	4.639277	2.181476	-2.930567
H	2.531801	2.079642	-2.516446
C	5.901103	0.1896	-2.452114
H	4.777849	-1.466241	-1.666703
C	5.854858	1.502962	-2.9089
H	4.59441	3.203711	-3.288778
H	6.842899	-0.34713	-2.43553
H	6.759176	1.994069	-3.248961

MA3-AI-TS-IP

Atom	X	Y	Z		
O	-1.517716	-0.617902	-0.836222	Electronic Energy (EE)	-1130.081995
O	0.330623	0.454109	-1.496357	Zero-point Energy Correction	0.38888
C	0.520125	-1.318551	0.134223	Thermal Correction to Energy	0.415326
C	-0.20102	-0.408737	-0.837188	Thermal Correction to Enthalpy	0.41627
C	1.835792	-1.84831	-0.439601	Thermal Correction to Free Energy	0.328848
H	-0.154896	-2.131595	0.405889		
H	2.368174	-1.020926	-0.917738		
C	-2.298251	0.257576	-1.665396		
H	-1.995409	0.153685	-2.707864		
H	-3.330063	-0.058985	-1.536203		
H	-2.173455	1.29114	-1.340771		
O	-2.571494	0.044181	2.789302		
O	-1.275959	-1.783084	2.974856		
C	-0.408922	0.179838	1.951369		
C	-1.433929	-0.637576	2.596742		
C	0.814021	-0.488531	1.415191		
H	-0.759973	1.096624	1.488978		
H	1.571543	0.264533	1.180664		
H	1.227141	-1.175553	2.157377		
C	-3.590996	-0.624447	3.543524		
H	-3.21907	-0.881071	4.536569		
H	-4.414205	0.082235	3.618731		
H	-3.914986	-1.527039	3.023997		
O	-2.73588	2.085562	5.264606		
O	-1.685704	3.190921	3.619061		

C	-0.642375	1.299565	4.631022
C	-1.704309	2.294611	4.438415
C	0.396558	1.235036	3.756528
H	-0.798418	0.545179	5.393614
H	1.191011	0.51536	3.919873
H	0.607257	2.072314	3.099616
C	-3.860451	2.958184	5.100124
H	-4.242751	2.892776	4.080671
H	-4.608341	2.611627	5.809131
H	-3.576387	3.987525	5.32227
H	2.455584	-2.201064	0.389467
C	1.681408	-3.012052	-1.449747
C	0.82034	-2.635167	-2.665858
H	-0.215399	-2.488646	-2.348976
H	1.183357	-1.722225	-3.141951
H	0.830598	-3.443747	-3.399552
C	1.133931	-4.282935	-0.780224
H	1.142998	-5.117055	-1.484781
H	1.73091	-4.553016	0.093374
H	0.101911	-4.117142	-0.466018
C	3.0387	-3.314631	-1.935932
N	4.099276	-3.55197	-2.31332

MA3-AI-TS-TL

Atom	X	Y	Z		
O	-1.516169	-0.634082	-0.871962	Electronic Energy (EE)	-1130.068266
O	0.329421	0.492266	-1.452728	Zero-point Energy Correction	0.38988
C	0.511326	-1.306656	0.145175	Thermal Correction to Energy	0.416395
C	-0.201348	-0.389902	-0.828816	Thermal Correction to Enthalpy	0.417339
C	1.824069	-1.839575	-0.434458	Thermal Correction to Free Energy	0.329514
H	-0.167032	-2.115469	0.420723		
H	2.355678	-1.009033	-0.907679		
C	-2.281884	0.237764	-1.712603		
H	-1.937007	0.166421	-2.744933		
H	-3.3113	-0.102003	-1.630874		
H	-2.193258	1.269203	-1.369591		
O	-2.563844	-0.030154	2.8567		
O	-1.232858	-1.839788	2.967168		
C	-0.417614	0.162966	1.985166		
C	-1.417854	-0.691797	2.626784		
C	0.808789	-0.481917	1.426759		
H	-0.790578	1.081587	1.544809		
H	1.556556	0.279695	1.188428		
H	1.232876	-1.170918	2.16114		
C	-3.553358	-0.740736	3.603751		

H	-3.163127	-1.023727	4.582515
H	-4.389256	-0.054357	3.717406
H	-3.867964	-1.636261	3.066206
O	-2.75169	2.147155	5.234428
O	-1.646141	3.197112	3.586336
C	-0.663344	1.305196	4.650032
C	-1.701253	2.321966	4.417388
C	0.388645	1.212855	3.795854
H	-0.848081	0.565411	5.419914
H	1.171265	0.485042	3.977626
H	0.617919	2.045639	3.139921
C	-3.844121	3.044171	5.029471
H	-4.214595	2.964621	4.006266
H	-4.612868	2.743255	5.737442
H	-3.536206	4.073064	5.220427
H	2.447291	-2.198886	0.389535
C	1.667669	-2.994617	-1.454353
C	0.80765	-2.598177	-2.665486
H	-0.228593	-2.453768	-2.349319
H	1.171047	-1.677872	-3.126241
H	0.818384	-3.393056	-3.413597
C	1.105339	-4.265251	-0.795991
H	1.108072	-5.095069	-1.50522
H	1.697371	-4.551781	0.075521
H	0.074413	-4.096173	-0.479922
C	3.024708	-3.304661	-1.941306
N	4.083754	-3.549079	-2.316324

MA3-CM-TS-IP

Atom	X	Y	Z		
O	-1.291141	-0.453056	-0.951885	Electronic Energy (EE)	-1344.067487
O	0.247264	0.98097	-1.713002	Zero-point Energy Correction	0.475774
C	0.900052	-0.697842	-0.109361	Thermal Correction to Energy	0.506031
C	-0.053077	0.033724	-1.022866	Thermal Correction to Enthalpy	0.506975
C	2.251765	-0.857367	-0.79506	Thermal Correction to Free Energy	0.409743
H	0.482099	-1.682758	0.110371		
H	2.119971	-1.405739	-1.735838		
H	2.67328	0.127384	-1.024279		
C	-2.291639	0.243953	-1.71172		
H	-2.045887	0.214196	-2.773307		
H	-3.222802	-0.283887	-1.522489		
H	-2.364828	1.278092	-1.373384		
O	3.098595	-1.569136	0.088955		
O	-2.283282	-0.398558	2.754329		
O	-0.531773	-1.807087	2.766765		

C	-0.288903	0.3717	1.845647
C	-1.015206	-0.730305	2.472404
C	1.036296	0.098122	1.215107
H	-0.902213	1.180671	1.462553
H	1.546838	1.042594	1.005346
H	1.661855	-0.492633	1.886886
C	-3.038379	-1.361137	3.501236
H	-2.553746	-1.56136	4.458047
H	-4.015966	-0.911522	3.659387
H	-3.136356	-2.287649	2.934252
O	-2.85613	1.371796	5.375844
O	-2.243473	2.812645	3.769149
C	-0.668007	1.228691	4.606081
C	-1.96951	1.902012	4.52505
C	0.2983	1.500345	3.68885
H	-0.570105	0.418325	5.319344
H	1.265677	1.017324	3.770683
H	0.2381	2.399429	3.084668
C	-4.183156	1.908741	5.318138
H	-4.594323	1.792374	4.314543
H	-4.764657	1.334593	6.035377
H	-4.177049	2.963849	5.594316
C	4.243066	-2.192428	-0.515723
C	5.037879	-2.750445	0.662276
H	5.908775	-3.301939	0.302042
H	5.376709	-1.932463	1.303415
H	4.414153	-3.427274	1.250925
C	5.076251	-1.155158	-1.265992
H	5.241629	-0.295526	-0.61188
H	6.050533	-1.567467	-1.533652
H	4.58552	-0.814447	-2.180451
C	3.78179	-3.350799	-1.404171
C	4.448966	-3.700573	-2.578537
C	2.685069	-4.120713	-1.003249
C	4.030718	-4.795023	-3.335183
H	5.301754	-3.123523	-2.916975
C	2.263071	-5.208632	-1.759198
H	2.155218	-3.857762	-0.09392
C	2.936589	-5.551282	-2.930233
H	4.562095	-5.051012	-4.245307
H	1.407277	-5.790357	-1.434447
H	2.609201	-6.39938	-3.521104

MA3-CM-TS-TL

Atom	X	Y	Z	Electronic Energy (EE)	-1344.056453

O	-1.315652	-0.433897	-0.96069	Zero-point Energy Correction	0.476813
O	0.219807	1.026696	-1.681542	Thermal Correction to Energy	0.50714
C	0.878253	-0.698384	-0.129884	Thermal Correction to Enthalpy	0.508084
C	-0.075022	0.06214	-1.022143	Thermal Correction to Free Energy	0.410635
C	2.225352	-0.852543	-0.826034		
H	0.455002	-1.68269	0.079586		
H	2.093457	-1.424197	-1.753617		
H	2.623149	0.135155	-1.084714		
C	-2.310907	0.285456	-1.697391		
H	-2.062669	0.299479	-2.758963		
H	-3.243465	-0.24825	-1.531956		
H	-2.388874	1.308978	-1.32838		
O	3.090594	-1.527564	0.064368		
O	-2.287813	-0.438767	2.746584		
O	-0.565181	-1.88254	2.666202		
C	-0.283187	0.335978	1.862375		
C	-1.026295	-0.786726	2.436315		
C	1.03296	0.064097	1.211272		
H	-0.884591	1.175393	1.529693		
H	1.557025	1.005635	1.021552		
H	1.656236	-0.557467	1.856406		
C	-3.050682	-1.426777	3.440604		
H	-2.567285	-1.691797	4.382254		
H	-4.020864	-0.973534	3.631631		
H	-3.164537	-2.322162	2.828001		
O	-2.871547	1.388104	5.353631		
O	-2.112513	2.828475	3.807716		
C	-0.673296	1.121521	4.639414		
C	-1.926066	1.884859	4.539248		
C	0.331844	1.362418	3.757805		
H	-0.649226	0.283766	5.326212		
H	1.267879	0.822205	3.842402		
H	0.33354	2.287001	3.19068		
C	-4.148878	2.020263	5.266559		
H	-4.536601	1.955982	4.248685		
H	-4.795588	1.481115	5.955061		
H	-4.078622	3.069299	5.557467		
C	4.236383	-2.147384	-0.53131		
C	5.046192	-2.663242	0.656072		
H	5.920279	-3.217393	0.308295		
H	5.37682	-1.82399	1.272716		
H	4.431954	-3.32775	1.266775		
C	5.051999	-1.117841	-1.312998		
H	5.208246	-0.239763	-0.682098		
H	6.031686	-1.52024	-1.5762		
H	4.552587	-0.805479	-2.232782		

C	3.786013	-3.334267	-1.387769
C	4.429218	-3.692343	-2.571889
C	2.717313	-4.118958	-0.943424
C	4.016658	-4.810168	-3.295527
H	5.258045	-3.102154	-2.944766
C	2.301068	-5.230116	-1.66616
H	2.207561	-3.844702	-0.026036
C	2.951627	-5.581267	-2.846885
H	4.528973	-5.072587	-4.214229
H	1.4678	-5.823809	-1.307603
H	2.628588	-6.447978	-3.411813

MA3-IP-TS-IP

Atom	X	Y	Z		
				Electronic Energy (EE)	-1113.069096
O	-1.530362	-0.684504	-0.879038	Zero-point Energy Correction	0.394263
O	0.306564	0.450778	-1.458769	Thermal Correction to Energy	0.420071
C	0.496406	-1.340387	0.150636	Thermal Correction to Enthalpy	0.421015
C	-0.22033	-0.437299	-0.829299	Thermal Correction to Free Energy	0.334927
C	1.80278	-1.89281	-0.422108		
H	-0.184249	-2.146001	0.431586		
H	2.371064	-1.070623	-0.868683		
C	-2.304433	0.176185	-1.728839		
H	-1.959471	0.093769	-2.760103		
H	-3.33054	-0.172153	-1.642864		
H	-2.223596	1.209437	-1.389549		
O	-2.572766	0.031583	2.83661		
O	-1.253166	-1.775466	3.046778		
C	-0.425693	0.170298	1.959023		
C	-1.430309	-0.641987	2.641946		
C	0.796563	-0.497157	1.420473		
H	-0.795845	1.070405	1.478783		
H	1.548471	0.257015	1.171824		
H	1.21864	-1.173619	2.167248		
C	-3.572274	-0.630372	3.62265		
H	-3.181333	-0.860912	4.614799		
H	-4.401624	0.069312	3.695921		
H	-3.895278	-1.547669	3.128891		
O	-2.712164	2.116766	5.290079		
O	-1.719455	3.202558	3.596385		
C	-0.624554	1.346494	4.619729		
C	-1.704678	2.322725	4.433549		
C	0.395079	1.278967	3.722776		
H	-0.751861	0.605695	5.40078		
H	1.204143	0.575156	3.883183		
H	0.578328	2.105513	3.044466		

C	-3.851573	2.972248	5.139174
H	-4.259721	2.883107	4.131604
H	-4.576244	2.629903	5.873879
H	-3.57496	4.009002	5.334746
H	2.397913	-2.287964	0.406892
C	1.662338	-3.010655	-1.461402
O	3.023835	-3.306723	-1.818094
H	3.015121	-4.022256	-2.46589
C	0.910145	-2.563214	-2.712336
H	-0.147036	-2.377554	-2.502634
H	1.355752	-1.656571	-3.128819
H	0.956752	-3.353252	-3.468059
C	1.022229	-4.262515	-0.867972
H	1.065812	-5.078896	-1.595277
H	1.555062	-4.572881	0.034687
H	-0.028038	-4.097704	-0.617515

MA3-TB-TS-IP

Atom	X	Y	Z		
O	-1.663628	-1.021381	-0.710078	Electronic Energy (EE)	-1152.355585
O	-0.102348	0.092891	-1.86116	Zero-point Energy Correction	0.422025
C	0.575958	-1.319684	-0.028302	Thermal Correction to Energy	0.449414
C	-0.404477	-0.678868	-0.979529	Thermal Correction to Enthalpy	0.450358
C	1.851146	-1.694006	-0.775112	Thermal Correction to Free Energy	0.359692
H	0.114394	-2.215871	0.392238		
H	1.603816	-2.330472	-1.633419		
H	2.32919	-0.783881	-1.150887		
C	-2.680383	-0.376919	-1.493951		
H	-2.550892	-0.617412	-2.549275		
H	-3.625112	-0.770673	-1.128062		
H	-2.636028	0.70293	-1.347959		
O	2.686729	-2.38426	0.132085		
O	-2.340196	-0.183358	2.915453		
O	-0.747071	-1.761923	3.069927		
C	-0.35313	0.1934	1.77488		
C	-1.137978	-0.700259	2.623081		
C	0.884918	-0.325431	1.122226		
H	-0.908881	0.99304	1.296332		
H	1.461848	0.510641	0.715645		
H	1.505834	-0.853594	1.848332		
C	-3.13304	-0.91693	3.85744		
H	-2.603318	-1.008896	4.806895		
H	-4.045848	-0.340311	3.988035		
H	-3.365699	-1.907829	3.465573		
O	-2.525259	2.080153	5.212512		

O	-1.878001	3.140663	3.344774
C	-0.431055	1.53957	4.360253
C	-1.651727	2.344143	4.233617
C	0.487917	1.535604	3.358012
H	-0.370196	0.854388	5.198034
H	1.400747	0.961085	3.469851
H	0.480354	2.322449	2.611071
C	-3.782022	2.764092	5.13549
H	-4.281247	2.531776	4.193841
H	-4.367894	2.39908	5.975579
H	-3.632315	3.841294	5.218028
C	4.092569	-2.44095	-0.190988
C	4.301921	-2.929688	-1.621898
H	3.944343	-2.202734	-2.355337
H	5.367911	-3.088616	-1.801349
H	3.780553	-3.877549	-1.781888
C	4.661432	-3.444836	0.802176
H	5.741296	-3.540812	0.669094
H	4.465236	-3.116615	1.826192
H	4.202746	-4.426036	0.65624
C	4.73046	-1.069309	0.022992
H	4.546465	-0.723818	1.043919
H	5.81071	-1.135562	-0.129366
H	4.3435	-0.323773	-0.675382

MA3-TB-TS-TL

Atom	X	Y	Z		
O	-1.658662	-1.01412	-0.709204	Electronic Energy (EE)	-1152.343518
O	-0.093948	0.149682	-1.807739	Zero-point Energy Correction	0.422639
C	0.578123	-1.31298	-0.012967	Thermal Correction to Energy	0.450246
C	-0.396161	-0.648943	-0.957179	Thermal Correction to Enthalpy	0.45119
C	1.856792	-1.669863	-0.762563	Thermal Correction to Free Energy	0.358737
H	0.117054	-2.215214	0.393461		
H	1.616205	-2.327723	-1.607564		
H	2.301224	-0.753165	-1.165096		
C	-2.663201	-0.351017	-1.484897		
H	-2.512946	-0.543707	-2.547457		
H	-3.612396	-0.764875	-1.153791		
H	-2.633951	0.724102	-1.302455		
O	2.714048	-2.315476	0.149181		
O	-2.350881	-0.230527	2.934634		
O	-0.763557	-1.819778	3.052937		
C	-0.352941	0.167851	1.817236		
C	-1.146423	-0.749639	2.636239		
C	0.885358	-0.345949	1.160087		

H	-0.90265	0.984637	1.361408
H	1.475966	0.491651	0.775705
H	1.494288	-0.902934	1.874148
C	-3.145932	-0.993687	3.843143
H	-2.626847	-1.118191	4.794822
H	-4.061752	-0.424841	3.987293
H	-3.370946	-1.974934	3.422992
O	-2.573658	2.106593	5.195585
O	-1.809349	3.158685	3.365401
C	-0.471026	1.489318	4.413815
C	-1.653501	2.34731	4.247345
C	0.481697	1.463495	3.445838
H	-0.46802	0.793183	5.244153
H	1.370961	0.857722	3.577436
H	0.520433	2.263793	2.714804
C	-3.787956	2.847809	5.073749
H	-4.265291	2.641622	4.114459
H	-4.422125	2.516966	5.893079
H	-3.593784	3.918214	5.154758
C	4.101268	-2.413901	-0.213796
C	4.261543	-2.998615	-1.616184
H	3.89413	-2.315903	-2.386331
H	5.318031	-3.18922	-1.818716
H	3.720108	-3.944723	-1.698579
C	4.687187	-3.360508	0.825474
H	5.760497	-3.488422	0.668094
H	4.525879	-2.961805	1.829594
H	4.202973	-4.337322	0.761318
C	4.769462	-1.042185	-0.111579
H	4.60756	-0.621099	0.883939
H	5.845789	-1.136692	-0.275317
H	4.385575	-0.339737	-0.854837

MA3-TL-TS-TL

Atom	X	Y	Z		
O	-1.421178	-0.871794	-0.932204	Electronic Energy (EE)	-1190.236716
O	0.409265	0.311263	-1.438442	Zero-point Energy Correction	0.415609
C	0.56397	-1.40433	0.248534	Thermal Correction to Energy	0.442505
C	-0.126448	-0.562358	-0.804337	Thermal Correction to Enthalpy	0.443449
C	1.864833	-1.984633	-0.307969	Thermal Correction to Free Energy	0.3518
H	-0.114232	-2.207977	0.545213		
H	2.514279	-1.163226	-0.626217		
C	-2.163094	-0.073162	-1.86133		
H	-1.746391	-0.170479	-2.86424		
H	-3.180063	-0.45645	-1.833941		

H	-2.14387	0.974881	-1.559928
O	-2.512726	-0.119958	2.970046
O	-1.072469	-1.82912	3.21995
C	-0.402311	0.11062	2.023146
C	-1.333491	-0.733954	2.772949
C	0.843226	-0.510721	1.481874
H	-0.840547	0.964423	1.517128
H	1.554734	0.26831	1.193902
H	1.305055	-1.141426	2.245514
C	-3.440672	-0.811747	3.807632
H	-3.018276	-0.964439	4.801958
H	-4.31988	-0.174334	3.867126
H	-3.701843	-1.77737	3.372672
O	-2.746246	2.223401	5.202464
O	-1.760085	3.20911	3.442595
C	-0.635626	1.454263	4.597462
C	-1.736332	2.393881	4.334009
C	0.388782	1.353624	3.710831
H	-0.75052	0.765078	5.425731
H	1.218625	0.687086	3.91655
H	0.545556	2.144018	2.984851
C	-3.892312	3.045763	4.979714
H	-4.295831	2.873969	3.980643
H	-4.617081	2.757585	5.737669
H	-3.634021	4.100329	5.085427
H	2.382834	-2.518569	0.494559
C	1.629479	-2.938677	-1.485829
H	1.103109	-2.407059	-2.285667
H	0.983849	-3.760849	-1.162909
C	2.928135	-3.48724	-2.021845
C	3.678967	-2.75415	-2.943642
C	3.431749	-4.708176	-1.570683
C	4.901273	-3.230394	-3.405944
H	3.297544	-1.802515	-3.30205
C	4.654753	-5.188463	-2.03028
H	2.857477	-5.289082	-0.855355
C	5.393358	-4.450252	-2.949613
H	5.468925	-2.650689	-4.125035
H	5.028985	-6.140973	-1.672377
H	6.344443	-4.823883	-3.31086

MAA-C2-IP-TS-IP

Atom	X	Y	Z		
				Electronic Energy (EE)	-500.1320326
O	0.354112	0.361809	-3.112903	Zero-point Energy Correction	0.191917
O	1.526109	1.873513	-1.963952	Thermal Correction to Energy	0.203745

C	2.139663	-0.444186	-1.79672	Thermal Correction to Enthalpy	0.20469
C	1.348642	0.712772	-2.275176	Thermal Correction to Free Energy	0.15489
C	3.275601	-0.149256	-1.071411		
H	3.994692	-0.926439	-0.841764		
H	3.449655	0.842039	-0.672127		
H	-0.111417	1.167518	-3.387355		
C	2.061333	-1.724564	-2.59782		
H	1.048418	-2.124243	-2.648473		
H	2.400991	-1.545162	-3.622308		
H	2.716312	-2.476775	-2.155251		
O	-0.535231	-1.422257	-0.993837		
C	0.520401	-0.919999	-0.287994		
C	0.277302	0.369047	0.432436		
C	1.223881	-2.000547	0.464755		
H	1.223718	0.822662	0.73485		
H	-0.278993	1.082441	-0.181576		
H	-0.311389	0.169327	1.337271		
H	2.146554	-1.621616	0.90602		
H	1.450976	-2.852147	-0.179965		
H	0.574625	-2.352882	1.276414		
H	-1.041011	-0.704471	-1.39882		

MAA-C3-AI-TS-IP

Atom	X	Y	Z		
				Electronic Energy (EE)	-517.154352
O	-0.602665	0.108814	-1.633827	Zero-point Energy Correction	0.186277
O	-0.158018	1.498627	0.060839	Thermal Correction to Energy	0.199198
C	0.872789	-0.663752	0.020405	Thermal Correction to Enthalpy	0.200142
C	0.006901	0.422758	-0.479087	Thermal Correction to Free Energy	0.145728
C	1.4937	-0.46968	1.218647		
H	2.261202	-1.164734	1.542283		
H	1.486447	0.514726	1.673762		
H	-1.148674	0.860643	-1.913525		
C	0.985423	-1.928078	-0.775011		
H	0.009975	-2.407377	-0.903498		
H	1.372516	-1.723477	-1.777795		
H	1.657895	-2.629214	-0.279639		
C	0.095422	-1.282963	2.785121		
C	0.095654	-2.753065	2.483383		
H	1.0964	-3.183423	2.542611		
H	-0.545473	-3.274063	3.20532		
H	-0.317108	-2.925294	1.487537		
C	-1.170794	-0.515726	2.546321		
H	-1.931043	-0.831854	3.271293		
H	-1.023724	0.560161	2.648504		
H	-1.553714	-0.736797	1.546852		

C	0.89646	-0.862952	3.879974
N	1.584158	-0.503828	4.738511

MAA-C3-AI-TS-TL

Atom	X	Y	Z
O	-0.586223	0.113891	-1.658969
O	-0.209984	1.46935	0.078169
C	0.864588	-0.670766	0.015177
C	-0.009168	0.41524	-0.47751
C	1.48047	-0.474946	1.215419
H	2.258271	-1.15798	1.539328
H	1.462753	0.510563	1.667025
H	-1.126035	0.874083	-1.918732
C	0.996803	-1.924816	-0.794382
H	0.026937	-2.40597	-0.952223
H	1.399463	-1.705279	-1.787357
H	1.664043	-2.630974	-0.299029
C	0.098876	-1.282184	2.796832
C	0.088646	-2.753844	2.497082
H	1.083112	-3.195691	2.574051
H	-0.567532	-3.272466	3.206834
H	-0.30686	-2.925868	1.494023
C	-1.165674	-0.510598	2.558339
H	-1.915629	-0.786992	3.309543
H	-1.004907	0.566503	2.610955
H	-1.572529	-0.759881	1.575409
C	0.904873	-0.866876	3.892137
N	1.599435	-0.515044	4.746859

Electronic Energy (EE)	-517.1451446
Zero-point Energy Correction	0.187011
Thermal Correction to Energy	0.199888
Thermal Correction to Enthalpy	0.200832
Thermal Correction to Free Energy	0.146774

MAA-C3-CM-TS-IP

Atom	X	Y	Z
O	-1.529106	0.5427	-0.066588
O	-0.680441	1.907824	-1.620489
C	0.467598	-0.13124	-1.10574
C	-0.612889	0.879645	-0.983304
C	1.468179	0.139935	-1.994461
H	2.244994	-0.595899	-2.165251
H	1.329977	0.904973	-2.745602
H	-2.203213	1.240294	-0.027686
O	2.644231	1.564867	-1.117059
C	3.127404	1.404253	0.196722
C	4.272786	2.438958	0.215366
C	2.041602	1.717469	1.223251
C	3.697069	0.001888	0.4295

Electronic Energy (EE)	-731.1270316
Zero-point Energy Correction	0.274523
Thermal Correction to Energy	0.290615
Thermal Correction to Enthalpy	0.291559
Thermal Correction to Free Energy	0.229979

C	4.31514	3.495879	1.124781
C	5.30623	2.315472	-0.721362
C	5.38063	4.395716	1.114031
C	6.36466	3.21546	-0.735319
C	6.406852	4.260089	0.186289
H	1.261139	0.956515	1.166332
H	2.440801	1.707421	2.240306
H	1.589646	2.692165	1.026063
H	2.89127	-0.734721	0.43484
H	4.20478	-0.039762	1.396457
H	4.411145	-0.264885	-0.351851
H	3.527277	3.626894	1.855796
H	5.278752	1.511538	-1.448049
H	5.401653	5.206435	1.833983
H	7.156368	3.10348	-1.467831
H	7.232362	4.96296	0.177869
C	0.408167	-1.363136	-0.260082
H	0.420103	-1.131226	0.809068
H	-0.525163	-1.900597	-0.454562
H	1.241943	-2.025844	-0.491119

MAA-C3-CM-TS-TL

Atom	X	Y	Z		
O	-1.49143	0.532134	-0.00887	Electronic Energy (EE)	-731.117855
O	-0.790483	1.818996	-1.697137	Zero-point Energy Correction	0.274867
C	0.472814	-0.134013	-1.115916	Thermal Correction to Energy	0.291016
C	-0.636408	0.846558	-1.003259	Thermal Correction to Enthalpy	0.29196
C	1.473336	0.169256	-1.99524	Thermal Correction to Free Energy	0.230182
H	2.257485	-0.553172	-2.185722		
H	1.315121	0.945776	-2.729957		
H	-2.180173	1.212098	0.007314		
O	2.660571	1.547252	-1.128743		
C	3.118214	1.413963	0.190802		
C	4.270117	2.440876	0.213258		
C	2.015685	1.753522	1.192855		
C	3.683437	0.0155	0.465674		
C	4.338085	3.476611	1.14351		
C	5.288358	2.325117	-0.739226		
C	5.414052	4.362711	1.137969		
C	6.357237	3.211226	-0.748353		
C	6.425776	4.233822	0.194876		
H	1.22213	1.00755	1.123382		
H	2.388273	1.744301	2.219991		
H	1.584968	2.732149	0.97226		

H	2.880282	-0.723909	0.46768
H	4.173607	-0.009574	1.441908
H	4.414584	-0.26234	-0.295647
H	3.558747	3.604278	1.883759
H	5.231156	1.541861	-1.485675
H	5.454227	5.158383	1.873172
H	7.135831	3.107412	-1.495446
H	7.259121	4.926792	0.189742
C	0.438778	-1.366799	-0.267157
H	0.471761	-1.135004	0.801631
H	-0.494493	-1.911619	-0.435775
H	1.271181	-2.026196	-0.513358

MAA-C3-IP-TS-IP

Atom	X	Y	Z		
O	0.610462	0.237384	-3.535101	Electronic Energy (EE)	-500.139345
O	1.043498	1.813681	-2.012903	Zero-point Energy Correction	0.191517
C	2.122965	-0.324282	-1.829071	Thermal Correction to Energy	0.204161
C	1.231189	0.685949	-2.428498	Thermal Correction to Enthalpy	0.205105
C	2.762518	0.008546	-0.691676	Thermal Correction to Free Energy	0.15116
H	3.48022	-0.668862	-0.241645		
H	2.696693	1.011795	-0.286254		
H	0.044826	0.946067	-3.879521		
C	2.225146	-1.67803	-2.474829		
H	1.25234	-2.176411	-2.515094		
H	2.58954	-1.60257	-3.503115		
H	2.913031	-2.30969	-1.910733		
O	2.01846	-0.064543	2.352598		
C	1.328837	-0.622714	1.304739		
C	1.366229	-2.106272	1.180114		
C	0.096129	0.129452	0.956242		
H	0.970146	-2.413482	0.209702		
H	2.386957	-2.4897	1.275816		
H	0.753916	-2.583646	1.960448		
H	-0.257911	-0.16317	-0.035638		
H	0.280841	1.206528	0.961518		
H	-0.711097	-0.079635	1.674603		
H	2.740918	-0.647429	2.619044		

MAA-C3-TB-TS-IP

Atom	X	Y	Z		
				Electronic Energy (EE)	-539.4189682
O	-0.345497	1.044607	-0.792868	Zero-point Energy Correction	0.220255
O	1.729378	0.479977	-1.4036	Thermal Correction to Energy	0.23359
C	0.694629	-0.667867	0.429986	Thermal Correction to Enthalpy	0.234534

C	0.776835	0.324619	-0.669667	Thermal Correction to Free Energy	0.179936
C	1.815712	-1.385536	0.729644		
H	1.737005	-2.260134	1.360782		
H	2.706466	-1.288723	0.120941		
H	-0.245558	1.660223	-1.536711		
O	2.49125	-0.47696	2.43872		
C	2.963113	0.845196	2.387382		
C	3.693599	0.982963	3.741172		
C	1.819802	1.857647	2.325559		
C	3.955169	1.047412	1.244207		
H	4.508592	0.259955	3.810965		
H	2.999969	0.822188	4.569139		
H	4.106263	1.99213	3.816922		
H	1.087909	1.649778	3.110349		
H	2.201425	2.872516	2.4657		
H	1.313528	1.822586	1.357794		
H	4.754584	0.303966	1.3014		
H	4.400714	2.043919	1.302649		
H	3.454359	0.953592	0.278115		
C	-0.586178	-0.807213	1.186975		
H	-0.839245	0.129732	1.693204		
H	-1.417249	-1.042482	0.516089		
H	-0.498656	-1.595173	1.935		

MAA-C3-TB-TS-TL

Atom	X	Y	Z		
				Electronic Energy (EE)	-539.4116127
O	-0.391866	1.007172	-0.812803	Zero-point Energy Correction	0.220983
O	1.7273	0.551657	-1.350903	Thermal Correction to Energy	0.234307
C	0.68763	-0.665613	0.435249	Thermal Correction to Enthalpy	0.235251
C	0.764912	0.336662	-0.653491	Thermal Correction to Free Energy	0.180811
C	1.819641	-1.366977	0.732189		
H	1.759793	-2.245858	1.35824		
H	2.703741	-1.252223	0.11737		
H	-0.269558	1.627307	-1.546018		
O	2.476345	-0.464971	2.428345		
C	2.965218	0.845321	2.380133		
C	3.682768	0.97338	3.741885		
C	1.83503	1.874091	2.308221		
C	3.97319	1.038349	1.247447		
H	4.485306	0.238097	3.818775		
H	2.978479	0.817218	4.560965		
H	4.109115	1.976025	3.827015		
H	1.095386	1.67371	3.086881		
H	2.224306	2.886118	2.447826		
H	1.33533	1.842305	1.337192		

H	4.758409	0.280939	1.311441
H	4.436692	2.026136	1.314057
H	3.485029	0.959162	0.273573
C	-0.592036	-0.823188	1.192105
H	-0.857409	0.109608	1.698551
H	-1.421447	-1.068127	0.5234
H	-0.493384	-1.607384	1.942202

MAA-C3-TL-TS-TL

Atom	X	Y	Z		
O	-1.471172	-0.65137	-0.475058	Electronic Energy (EE)	-577.3087131
O	-0.533735	1.308697	0.050281	Zero-point Energy Correction	0.212892
C	0.856152	-0.628785	-0.152004	Thermal Correction to Energy	0.225817
C	-0.407945	0.126197	-0.175528	Thermal Correction to Enthalpy	0.226761
C	1.991185	0.074685	0.111535	Thermal Correction to Free Energy	0.170742
H	2.959713	-0.406102	0.031089		
H	1.972729	1.157603	0.085562		
H	-2.2529	-0.081467	-0.469449		
C	4.597619	2.986033	2.72491		
C	3.408694	2.288861	2.581603		
C	3.397596	0.878551	2.553554		
C	4.630709	0.20205	2.660438		
C	5.81621	0.90523	2.803838		
C	5.807394	2.299953	2.837363		
H	4.586179	4.069735	2.749658		
H	2.468671	2.823884	2.491731		
H	4.640202	-0.883103	2.636925		
H	6.75374	0.368179	2.892038		
H	6.735562	2.847547	2.949704		
C	2.173516	0.158337	2.362845		
H	2.163621	-0.914414	2.515682		
H	1.230954	0.677812	2.485227		
C	0.823239	-2.121033	-0.290877		
H	0.237235	-2.581641	0.510821		
H	0.358716	-2.422791	-1.232931		
H	1.835288	-2.526643	-0.257763		

MAA2-AI-TS-IP

Atom	X	Y	Z		
O	-1.726509	0.083599	-0.654436	Electronic Energy (EE)	-823.6523331
O	-0.04706	1.54694	-0.422677	Zero-point Energy Correction	0.287645
C	0.366651	-0.767561	-0.012977	Thermal Correction to Energy	0.306737
C	-0.446288	0.398145	-0.385259	Thermal Correction to Enthalpy	0.307681
C	1.846333	-0.529229	0.11145	Thermal Correction to Free Energy	0.239707

H	2.005122	0.483184	0.488128
H	-2.210711	0.904797	-0.835901
C	-0.153454	-2.133443	-0.342488
H	0.478428	-2.903494	0.105016
H	-1.179049	-2.266349	0.001376
H	-0.161456	-2.287754	-1.428243
O	-3.623369	0.154719	2.446827
O	-1.912987	1.566971	2.161331
C	-1.471133	-0.778182	2.329471
C	-2.317467	0.429652	2.298924
C	-0.125439	-0.612056	2.145756
H	0.53232	-1.456324	2.323549
H	0.307732	0.380542	2.209098
H	-4.119221	0.988315	2.423562
C	-2.124429	-2.114672	2.492186
H	-2.675249	-2.163936	3.436643
H	-2.849377	-2.302645	1.693708
H	-1.377061	-2.908613	2.48382
H	2.264788	-1.230232	0.839558
C	2.668244	-0.690421	-1.208464
C	2.076044	0.113411	-2.374859
H	1.088864	-0.285399	-2.623203
H	1.975893	1.169046	-2.119587
H	2.713718	0.019094	-3.256226
C	2.864495	-2.160529	-1.614515
H	3.23979	-2.752459	-0.777169
H	1.919563	-2.583269	-1.954028
H	3.578294	-2.227477	-2.438509
C	4.002677	-0.142878	-0.909155
N	5.046368	0.279417	-0.672598

MAA2-AI-TS-TL

Atom	X	Y	Z		
O	-1.667746	0.243393	-0.718868	Electronic Energy (EE)	-823.635325
O	0.081077	1.614628	-0.438717	Zero-point Energy Correction	0.288536
C	0.363262	-0.716711	-0.025328	Thermal Correction to Energy	0.3077
C	-0.377708	0.495974	-0.413841	Thermal Correction to Enthalpy	0.308644
C	1.852768	-0.556636	0.110697	Thermal Correction to Free Energy	0.2404
H	2.056027	0.441199	0.504962		
H	-2.100066	1.102466	-0.833074		
C	-0.231814	-2.051867	-0.356455		
H	0.345153	-2.859351	0.100244		
H	-1.267558	-2.11667	-0.02438		
H	-0.24266	-2.215887	-1.440498		
O	-3.709801	-0.065868	2.454403		

O	-2.172617	1.485239	1.973922
C	-1.474213	-0.775957	2.324479
C	-2.443113	0.334521	2.224908
C	-0.153788	-0.480656	2.122199
H	0.58858	-1.233488	2.363433
H	0.167089	0.555596	2.119577
H	-4.276514	0.715467	2.384547
C	-1.980374	-2.158538	2.59647
H	-2.475151	-2.203915	3.571429
H	-2.724976	-2.465902	1.856459
H	-1.159113	-2.87598	2.591081
H	2.235475	-1.288111	0.829116
C	2.679705	-0.731714	-1.204642
C	2.1362	0.126172	-2.356089
H	1.13896	-0.226477	-2.633216
H	2.065355	1.17555	-2.069735
H	2.783734	0.036151	-3.230335
C	2.795053	-2.20215	-1.640377
H	3.144848	-2.83135	-0.819237
H	1.828735	-2.571565	-1.982275
H	3.501331	-2.293563	-2.467984
C	4.042075	-0.266941	-0.88402
N	5.106849	0.08385	-0.628745

MAA2-CM-TS-IP

Atom	X	Y	Z		
O	-1.611004	-0.543807	-0.837015	Electronic Energy (EE)	-1037.6403
O	-0.836616	1.556592	-0.714355	Zero-point Energy Correction	0.374165
C	0.500626	-0.212099	0.136553	Thermal Correction to Energy	0.397226
C	-0.677866	0.365795	-0.505678	Thermal Correction to Enthalpy	0.398171
C	1.634061	0.751347	0.326557	Thermal Correction to Free Energy	0.320026
H	2.087645	0.940037	-0.66122		
H	1.260718	1.707961	0.704261		
H	-2.371216	-0.075812	-1.216775		
C	0.810972	-1.661914	-0.045695		
H	1.502954	-2.008446	0.722799		
H	-0.090179	-2.272954	-0.022738		
H	1.295171	-1.81043	-1.020629		
O	2.5859	0.19102	1.208981		
O	-3.7618	-1.294359	1.920082		
O	-2.965159	0.779187	1.666587		
C	-1.454619	-0.988258	2.23887		
C	-2.766297	-0.393687	1.915055		
C	-0.370938	-0.16213	2.215294		
H	0.589375	-0.523526	2.567659		

H	-0.510417	0.912491	2.160661
H	-4.591855	-0.838504	1.708929
C	-1.368287	-2.461787	2.490228
H	-1.986751	-2.748121	3.346323
H	-1.73258	-3.032101	1.63003
H	-0.337138	-2.752305	2.693384
C	3.886553	0.805317	1.186213
C	4.651161	0.107806	2.308355
H	5.682665	0.464614	2.3395
H	4.174644	0.317752	3.269426
H	4.660048	-0.972417	2.145692
C	3.774369	2.299088	1.481815
H	3.160081	2.436638	2.375059
H	4.760702	2.722406	1.680228
H	3.324749	2.854103	0.65568
C	4.564244	0.50303	-0.152584
C	4.431164	-0.776833	-0.701844
C	5.342484	1.441817	-0.829196
C	5.054118	-1.107467	-1.899575
H	3.825952	-1.515125	-0.186539
C	5.973726	1.110325	-2.0284
H	5.463845	2.44259	-0.431445
C	5.830547	-0.162362	-2.568517
H	4.934896	-2.103426	-2.3121
H	6.57384	1.854259	-2.540773
H	6.317648	-0.417785	-3.502916

MAA2-CM-TS-TL

Atom	X	Y	Z		
O	-1.676095	-0.368148	-0.926085	Electronic Energy (EE)	-1037.627661
O	-0.827377	1.701774	-0.774888	Zero-point Energy Correction	0.375236
C	0.445033	-0.120991	0.056157	Thermal Correction to Energy	0.398347
C	-0.712206	0.510928	-0.581583	Thermal Correction to Enthalpy	0.399291
C	1.61797	0.791926	0.253599	Thermal Correction to Free Energy	0.320957
H	2.094933	0.961285	-0.726759		
H	1.27843	1.764562	0.622971		
H	-2.439439	0.15322	-1.213558		
C	0.698152	-1.581301	-0.130321		
H	1.375776	-1.952607	0.639821		
H	-0.229161	-2.151805	-0.109768		
H	1.17616	-1.750921	-1.10467		
O	2.528903	0.187214	1.148895		
O	-3.651509	-1.589644	2.044472		
O	-3.132294	0.526468	1.546915		
C	-1.394382	-0.966057	2.245916		

C	-2.776995	-0.57178	1.906456
C	-0.428076	-0.012274	2.129272
H	0.574933	-0.208361	2.493889
H	-0.718314	1.023271	1.985102
H	-4.526936	-1.249067	1.812566
C	-1.116879	-2.384988	2.638426
H	-1.674096	-2.655457	3.540116
H	-1.428521	-3.084012	1.857059
H	-0.053093	-2.525277	2.832812
C	3.844042	0.760542	1.178946
C	4.549494	0.017942	2.311569
H	5.591708	0.335144	2.382062
H	4.048369	0.224938	3.26025
H	4.525385	-1.058063	2.128185
C	3.769586	2.252094	1.502392
H	3.123434	2.393273	2.371907
H	4.759	2.63944	1.752096
H	3.373304	2.839643	0.671666
C	4.557879	0.464985	-0.142755
C	4.39277	-0.794097	-0.728239
C	5.39834	1.384952	-0.767399
C	5.047134	-1.123585	-1.908338
H	3.733961	-1.51277	-0.253027
C	6.060847	1.054707	-1.948996
H	5.542954	2.371171	-0.342568
C	5.88716	-0.197858	-2.5236
H	4.903057	-2.103373	-2.349657
H	6.709649	1.783839	-2.420772
H	6.399149	-0.452357	-3.444304

MAA2-IP-TS-IP

Atom	X	Y	Z		
O	-1.73767	0.093655	-0.662752	Electronic Energy (EE)	-806.639902
O	-0.056991	1.554613	-0.430725	Zero-point Energy Correction	0.292995
C	0.356937	-0.762307	-0.02724	Thermal Correction to Energy	0.311426
C	-0.455048	0.404991	-0.39493	Thermal Correction to Enthalpy	0.31237
C	1.838732	-0.538423	0.076573	Thermal Correction to Free Energy	0.246481
H	2.020175	0.47801	0.433067		
H	-2.219831	0.917205	-0.838021		
C	-0.175097	-2.125101	-0.351021		
H	0.453296	-2.898305	0.096247		
H	-1.200776	-2.251244	-0.004967		
H	-0.185051	-2.281942	-1.436507		
O	-3.625377	0.135803	2.469046		
O	-1.933987	1.564823	2.15485		

C	-1.464936	-0.774498	2.33216
C	-2.323418	0.423254	2.303996
C	-0.123036	-0.596949	2.131025
H	0.544572	-1.433832	2.306363
H	0.302961	0.399378	2.182598
H	-4.129791	0.964151	2.446058
C	-2.101788	-2.117339	2.511964
H	-2.639899	-2.167168	3.463701
H	-2.834132	-2.3197	1.723892
H	-1.345362	-2.902691	2.49921
H	2.258036	-1.233231	0.811139
C	2.658659	-0.707498	-1.224161
C	2.084868	0.100773	-2.382455
H	1.093392	-0.264302	-2.666967
H	2.005597	1.155714	-2.112787
H	2.740041	0.008907	-3.253958
C	2.842726	-2.168992	-1.623291
H	3.221076	-2.753195	-0.780644
H	1.913106	-2.616794	-1.975441
H	3.569056	-2.22934	-2.43998
O	3.942648	-0.172609	-0.860537
H	4.541793	-0.305635	-1.605149

MAA2-TB-TS-IP

Atom	X	Y	Z		
O	-1.607072	-0.960178	-0.808172	Electronic Energy (EE)	-845.9283347
O	-0.467985	0.889775	-1.362238	Zero-point Energy Correction	0.320783
C	0.583501	-0.736195	0.011236	Thermal Correction to Energy	0.340788
C	-0.509362	-0.183673	-0.784918	Thermal Correction to Enthalpy	0.341733
C	1.871055	0.027493	-0.090182	Thermal Correction to Free Energy	0.271727
H	2.28869	-0.139756	-1.096839		
H	1.676308	1.099126	0.004587		
H	-2.29497	-0.500382	-1.314781		
C	0.640089	-2.203114	0.28682		
H	1.284923	-2.410049	1.141795		
H	-0.349487	-2.619419	0.469202		
H	1.070525	-2.719115	-0.582209		
O	2.767566	-0.436403	0.896744		
O	-3.707653	-0.340938	1.997363		
O	-2.519471	1.357574	1.158875		
C	-1.371931	-0.44898	2.230496		
C	-2.551017	0.290128	1.739029		
C	-0.147141	0.090664	1.973481		
H	0.740605	-0.348631	2.416195		
H	-0.072819	1.110337	1.60942		

H	-4.437241	0.198115	1.653077
C	-1.571326	-1.777757	2.890822
H	-2.198651	-1.681378	3.782058
H	-2.077252	-2.479616	2.220223
H	-0.61161	-2.203454	3.18532
C	3.876879	0.431818	1.217675
C	4.622658	0.850832	-0.046618
H	4.018826	1.510185	-0.674959
H	5.531053	1.391971	0.228582
H	4.908947	-0.028263	-0.630662
C	4.770972	-0.425237	2.102863
H	5.637941	0.149211	2.436309
H	4.218941	-0.763504	2.983683
H	5.123022	-1.301668	1.552955
C	3.378942	1.649642	1.994527
H	2.828752	1.331793	2.884215
H	4.231076	2.254423	2.314891
H	2.731104	2.288051	1.389375

MAA2-TB-TS-TL

Atom	X	Y	Z		
O	-1.596877	-0.831069	-0.962333	Electronic Energy (EE)	-845.9149444
O	-0.401988	1.003044	-1.449708	Zero-point Energy Correction	0.321357
C	0.561881	-0.649674	-0.048523	Thermal Correction to Energy	0.341595
C	-0.487419	-0.066748	-0.885936	Thermal Correction to Enthalpy	0.342539
C	1.868479	0.086068	-0.09533	Thermal Correction to Free Energy	0.271499
H	2.325247	-0.086694	-1.083949		
H	1.687171	1.162066	-0.011998		
H	-2.26415	-0.307746	-1.429287		
C	0.576432	-2.116766	0.228739		
H	1.202569	-2.334853	1.09462		
H	-0.429243	-2.502002	0.389949		
H	1.008545	-2.649915	-0.628781		
O	2.70639	-0.398673	0.928829		
O	-3.71324	-0.625008	2.134952		
O	-2.782364	1.095609	1.054008		
C	-1.370803	-0.449172	2.218642		
C	-2.651618	0.103773	1.732848		
C	-0.234489	0.216729	1.871044		
H	0.717622	-0.0696	2.305919		
H	-0.311591	1.204836	1.429053		
H	-4.505731	-0.195581	1.783281		
C	-1.379191	-1.726548	3.001309		
H	-1.954886	-1.613989	3.924635		
H	-1.848979	-2.536929	2.436549		

H	-0.361884	-2.021671	3.260119
C	3.864378	0.394809	1.248618
C	4.662522	0.734996	-0.008699
H	4.124327	1.428695	-0.659111
H	5.605741	1.208966	0.27227
H	4.890108	-0.172537	-0.574131
C	4.681413	-0.503174	2.167899
H	5.577695	0.015929	2.514367
H	4.086935	-0.790338	3.038363
H	4.98294	-1.410671	1.640127
C	3.441613	1.663081	1.990396
H	2.866824	1.404983	2.883292
H	4.325594	2.224542	2.302622
H	2.837413	2.324661	1.365574

MAA2-TL-TS-TL

Atom	X	Y	Z		
O	-1.583751	0.030181	-0.781877	Zero-point Energy Correction	0.314485
O	0.136083	1.464638	-0.684732	Thermal Correction to Energy	0.334041
C	0.473279	-0.776348	0.032653	Thermal Correction to Enthalpy	0.334985
C	-0.299666	0.348426	-0.508581	Thermal Correction to Free Energy	0.263567
C	1.947019	-0.538885	0.167401		
H	2.130313	0.454356	0.583838		
H	-2.030539	0.855285	-1.020133		
C	-0.006142	-2.173398	-0.208655		
H	0.517201	-2.877562	0.443313		
H	-1.08001	-2.267353	-0.051687		
H	0.198253	-2.470289	-1.245363		
O	-3.724037	-0.087124	2.247819		
O	-2.202998	1.487866	1.797562		
C	-1.46484	-0.7314	2.308444		
C	-2.455257	0.341612	2.086275		
C	-0.149407	-0.405473	2.158176		
H	0.612814	-1.116307	2.459545		
H	0.138577	0.637806	2.086794		
H	-4.305004	0.671475	2.095482		
C	-1.948889	-2.113539	2.623404		
H	-2.52131	-2.122556	3.555537		
H	-2.617108	-2.489578	1.843345		
H	-1.107588	-2.7996	2.727078		
H	2.379232	-1.278189	0.848981		
C	2.667295	-0.638569	-1.197536		
H	2.210684	0.07702	-1.886416		
H	2.527174	-1.6402	-1.612861		
C	4.139016	-0.345778	-1.054529		

Electronic Energy (EE): -883.806539

C	4.594025	0.97463	-1.023284
C	5.066233	-1.377123	-0.899762
C	5.944008	1.257045	-0.846016
H	3.880142	1.784383	-1.14247
C	6.41855	-1.098567	-0.721675
H	4.725589	-2.407846	-0.9249
C	6.861008	0.220032	-0.694299
H	6.281731	2.287054	-0.829238
H	7.126745	-1.911559	-0.608
H	7.91377	0.439037	-0.558755

MAA3-AI-TS-IP

Atom	X	Y	Z		
				Electronic Energy (EE)	-1130.146026
O	-1.392994	-1.294274	-0.959075	Zero-point Energy Correction	0.388707
O	-0.954052	0.849102	-1.383884	Thermal Correction to Energy	0.414212
C	0.465878	-0.340242	0.189645	Thermal Correction to Enthalpy	0.415156
C	-0.672039	-0.173959	-0.81004	Thermal Correction to Free Energy	0.332875
C	1.521579	0.763983	-0.023759		
H	0.998539	1.716864	-0.135405		
H	-2.137836	-1.109569	-1.554799		
C	1.068166	-1.747717	0.136882		
H	1.882007	-1.818584	0.862956		
H	0.330203	-2.510746	0.371496		
H	1.46928	-1.980901	-0.851238		
O	-3.626692	-1.265493	1.745523		
O	-2.844296	0.722426	1.073614		
C	-1.334987	-0.839883	2.055929		
C	-2.637809	-0.372176	1.564847		
C	-0.132938	-0.051929	1.613125		
H	0.683752	-0.250166	2.313771		
H	-0.367868	1.014227	1.663829		
H	-4.457726	-0.870333	1.4387		
C	-1.205924	-2.286465	2.430501		
H	-1.94009	-2.570133	3.186859		
H	-1.385569	-2.925909	1.558765		
H	-0.205065	-2.495839	2.811353		
O	1.547788	0.428975	5.536621		
O	0.471738	1.884616	4.223515		
C	-0.596955	-0.179746	4.802333		
C	0.491965	0.816153	4.802349		
C	-1.698589	0.088335	4.035551		
H	-2.589805	-0.516429	4.170441		
H	-1.824186	1.080487	3.615198		
H	2.218597	1.129771	5.509072		
C	-0.428334	-1.438858	5.594072		

H	0.42515	-2.02326	5.235793
H	-0.236729	-1.21199	6.647114
H	-1.326115	-2.053437	5.528301
H	2.115718	0.833435	0.892777
C	2.514683	0.668071	-1.209134
C	3.292706	1.997447	-1.277493
H	3.772761	2.219127	-0.321984
H	4.057258	1.95645	-2.055888
H	2.592173	2.800924	-1.514662
C	1.860593	0.396584	-2.572556
H	1.308844	-0.545497	-2.592696
H	1.1716	1.210374	-2.80198
H	2.628442	0.357632	-3.348311
C	3.521226	-0.378425	-0.952128
N	4.347184	-1.158389	-0.768887

MAA3-AI-TS-TL

Atom	X	Y	Z	Electronic Energy (EE)	-1130.124344
O	-1.438863	-1.300431	-0.915576	Zero-point Energy Correction	0.389936
O	-0.921338	0.787679	-1.497838	Thermal Correction to Energy	0.415505
C	0.452978	-0.331781	0.169516	Thermal Correction to Enthalpy	0.416449
C	-0.678553	-0.187941	-0.84255	Thermal Correction to Free Energy	0.333779
C	1.491028	0.788786	-0.051363		
H	0.944903	1.722731	-0.204525		
H	-2.1705	-1.108191	-1.52106		
C	1.069917	-1.734322	0.121629		
H	1.877494	-1.80547	0.854324		
H	0.332132	-2.501939	0.340946		
H	1.489376	-1.963116	-0.859282		
O	-3.650987	-1.229485	1.826396		
O	-2.850199	0.709856	1.046089		
C	-1.340075	-0.83604	2.048987		
C	-2.647138	-0.360243	1.574083		
C	-0.144993	-0.046628	1.592661		
H	0.675178	-0.236246	2.29165		
H	-0.384953	1.017916	1.646285		
H	-4.469978	-0.815696	1.519122		
C	-1.211439	-2.285337	2.412304		
H	-1.954137	-2.579073	3.15582		
H	-1.384005	-2.915622	1.532953		
H	-0.214267	-2.499608	2.800604		
O	1.565251	0.373671	5.581679		
O	0.563875	1.812095	4.193421		
C	-0.578068	-0.200706	4.810289		
C	0.536296	0.76926	4.804631		

C	-1.667391	0.092473	4.035855
H	-2.577613	-0.482869	4.170762
H	-1.760275	1.087297	3.613406
H	2.243348	1.062836	5.536921
C	-0.448261	-1.453364	5.620693
H	0.400302	-2.059483	5.289104
H	-0.268038	-1.217094	6.673233
H	-1.356171	-2.052473	5.549908
H	2.059094	0.902706	0.87731
C	2.519274	0.683065	-1.206105
C	3.265723	2.029301	-1.290588
H	3.712609	2.291462	-0.329525
H	4.055786	1.986867	-2.042607
H	2.5558	2.809973	-1.571771
C	1.905498	0.363466	-2.577823
H	1.380456	-0.593259	-2.587645
H	1.194458	1.146635	-2.841776
H	2.691272	0.325724	-3.33519
C	3.53715	-0.337549	-0.889187
N	4.359277	-1.105198	-0.649154

MAA3-CM-TS-IP

Atom	X	Y	Z		
				Electronic Energy (EE)	-1344.1362
O	-1.622136	-0.847546	-0.831126	Zero-point Energy Correction	0.475368
O	-0.776695	1.156654	-1.320261	Thermal Correction to Energy	0.504903
C	0.384953	-0.231666	0.294543	Thermal Correction to Enthalpy	0.505847
C	-0.710073	0.124088	-0.697413	Thermal Correction to Free Energy	0.41289
C	1.512999	0.790645	0.105571		
H	1.901961	0.721893	-0.918418		
H	1.120407	1.799577	0.261852		
H	-2.308117	-0.549148	-1.450836		
C	0.903937	-1.647281	0.011322		
H	1.690956	-1.892833	0.726183		
H	0.116556	-2.394662	0.089329		
H	1.323655	-1.699054	-0.997686		
O	2.528011	0.49952	1.047556		
O	-3.752114	-0.864957	1.831213		
O	-2.716972	1.057527	1.331498		
C	-1.418714	-0.776391	2.120084		
C	-2.654615	-0.097236	1.714363		
C	-0.140194	-0.07949	1.757184		
H	0.657909	-0.464702	2.396431		
H	-0.245986	0.988765	1.962344		
H	-4.52507	-0.33675	1.576809		
C	-1.447723	-2.265573	2.282681		

H	-2.225803	-2.580823	2.980213
H	-1.664244	-2.747852	1.322219
H	-0.48205	-2.629136	2.638153
O	1.673532	-0.189182	5.57322
O	0.648277	1.513965	4.548913
C	-0.545562	-0.535588	4.890554
C	0.614832	0.373325	4.966973
C	-1.65387	-0.091619	4.223322
H	-2.581155	-0.648095	4.313436
H	-1.72137	0.954135	3.942311
H	2.395979	0.458193	5.598042
C	-0.435218	-1.903971	5.488001
H	0.361447	-2.482714	5.009772
H	-0.188941	-1.845197	6.552433
H	-1.375436	-2.444574	5.377221
C	3.747908	1.244037	0.902849
C	4.589821	0.818625	2.103939
H	5.575602	1.28627	2.059691
H	4.094093	1.123738	3.029413
H	4.717391	-0.266432	2.110963
C	3.469967	2.744574	0.971005
H	2.979887	3.120061	0.070483
H	2.826491	2.943537	1.831868
H	4.401227	3.297386	1.108037
C	4.457073	0.819851	-0.385626
C	4.46433	-0.533422	-0.74036
C	5.134	1.72633	-1.201023
C	5.124647	-0.967773	-1.883836
H	3.937868	-1.248098	-0.11665
C	5.803093	1.292028	-2.345589
H	5.146114	2.781654	-0.954856
C	5.799179	-0.053997	-2.692303
H	5.113864	-2.020312	-2.145361
H	6.323269	2.012471	-2.967323
H	6.31509	-0.390371	-3.584663

MAA3-CM-TS-TL

Atom	X	Y	Z		
O	-1.668903	-0.823672	-0.821304	Electronic Energy (EE)	-1344.117209
O	-0.738652	1.116383	-1.411278	Zero-point Energy Correction	0.476395
C	0.366175	-0.234442	0.273165	Thermal Correction to Energy	0.506095
C	-0.713744	0.125458	-0.733372	Thermal Correction to Enthalpy	0.507039
C	1.509327	0.770093	0.083635	Thermal Correction to Free Energy	0.413361
H	1.891439	0.704752	-0.943147		
H	1.131588	1.783811	0.245832		

H	-2.337357	-0.502763	-1.444748
C	0.874943	-1.655931	-0.003564
H	1.645082	-1.909868	0.726628
H	0.077634	-2.394443	0.0485
H	1.318095	-1.708161	-1.002484
O	2.519984	0.449247	1.017707
O	-3.768978	-0.884103	1.914649
O	-2.763908	1.029326	1.332681
C	-1.424676	-0.779125	2.114355
C	-2.675815	-0.109855	1.733723
C	-0.160238	-0.067127	1.732802
H	0.651373	-0.417893	2.375252
H	-0.287245	1.001634	1.921615
H	-4.538693	-0.348971	1.675271
C	-1.440531	-2.269882	2.269771
H	-2.215597	-2.596082	2.965229
H	-1.660701	-2.747348	1.308242
H	-0.471354	-2.630664	2.61888
O	1.670686	-0.067532	5.602184
O	0.600621	1.576649	4.529175
C	-0.5237	-0.508083	4.886909
C	0.599709	0.448628	4.962882
C	-1.646167	-0.102032	4.220535
H	-2.558359	-0.681897	4.314598
H	-1.73995	0.942291	3.941617
H	2.35139	0.619726	5.62304
C	-0.362429	-1.869777	5.489587
H	0.462861	-2.416877	5.023423
H	-0.12546	-1.799105	6.55489
H	-1.277572	-2.4511	5.374432
C	3.736405	1.196859	0.904914
C	4.565082	0.741797	2.104932
H	5.554649	1.202461	2.0823
H	4.057844	1.027879	3.029553
H	4.684841	-0.343374	2.08943
C	3.458934	2.69651	1.009891
H	2.989884	3.099462	0.110252
H	2.795719	2.8715	1.860496
H	4.386471	3.245129	1.184519
C	4.462345	0.806026	-0.385228
C	4.461244	-0.535867	-0.778143
C	5.161721	1.726766	-1.163587
C	5.137929	-0.94575	-1.919979
H	3.910972	-1.256697	-0.183167
C	5.846521	1.317364	-2.307004
H	5.177065	2.774869	-0.889236

C	5.836337	-0.017871	-2.689746
H	5.121046	-1.989978	-2.211329
H	6.3835	2.048843	-2.900202
H	6.364881	-0.334789	-3.581313

MAA3-IP-TS-IP

Atom	X	Y	Z		
O	-1.389011	-1.273585	-0.975577	Electronic Energy (EE)	-1113.131019
O	-0.954886	0.875525	-1.36898	Zero-point Energy Correction	0.393762
C	0.472317	-0.330983	0.185077	Thermal Correction to Energy	0.418765
C	-0.667033	-0.154475	-0.809602	Thermal Correction to Enthalpy	0.419709
C	1.531852	0.770089	-0.027573	Thermal Correction to Free Energy	0.338691
H	1.009493	1.727924	-0.08774		
H	-2.13314	-1.077681	-1.568352		
C	1.066973	-1.742222	0.127575		
H	1.871492	-1.819052	0.862769		
H	0.321926	-2.503769	0.345376		
H	1.491265	-1.955177	-0.853103		
O	-3.617587	-1.273517	1.732683		
O	-2.846633	0.726742	1.085292		
C	-1.328403	-0.837974	2.051766		
C	-2.633215	-0.372244	1.563623		
C	-0.127954	-0.047204	1.609593		
H	0.689208	-0.245146	2.309814		
H	-0.365107	1.018247	1.663139		
H	-4.450336	-0.878924	1.429925		
C	-1.197231	-2.285471	2.422654		
H	-1.930094	-2.572088	3.179225		
H	-1.37687	-2.923003	1.549666		
H	-0.195581	-2.494412	2.80155		
O	1.553759	0.427196	5.527831		
O	0.467705	1.888558	4.229325		
C	-0.592352	-0.182788	4.798527		
C	0.493459	0.816048	4.80045		
C	-1.694818	0.083698	4.032108		
H	-2.584649	-0.523257	4.166486		
H	-1.823157	1.076521	3.614274		
H	2.222588	1.129864	5.500865		
C	-0.419444	-1.443854	5.586337		
H	0.432165	-2.027294	5.221963		
H	-0.222483	-1.21987	6.638978		
H	-1.317436	-2.058471	5.523454		
H	2.151213	0.804199	0.875238		
C	2.505964	0.69135	-1.21399		
O	3.454445	-0.344472	-0.900211		

H	4.106334	-0.371393	-1.611364
C	3.255902	2.021356	-1.299801
H	3.732213	2.251645	-0.343074
H	4.032307	1.963626	-2.068844
H	2.576905	2.837183	-1.558671
C	1.850564	0.387115	-2.559577
H	1.311047	-0.563189	-2.550321
H	1.15472	1.178803	-2.841587
H	2.627187	0.323731	-3.328261

MAA3-TB-TS-IP

Atom	X	Y	Z		
O	-1.553524	-1.05659	-0.901784	Electronic Energy (EE)	-1152.424389
O	-0.437775	0.76626	-1.537995	Zero-point Energy Correction	0.422046
C	0.481816	-0.592597	0.246954	Thermal Correction to Energy	0.448385
C	-0.525737	-0.202147	-0.821292	Thermal Correction to Enthalpy	0.449329
C	1.755554	0.224424	-0.012479	Thermal Correction to Free Energy	0.365704
H	2.122876	0.010855	-1.023807		
H	1.520719	1.290165	0.04965		
H	-2.180635	-0.731212	-1.56832		
C	0.799691	-2.090343	0.14977		
H	1.508643	-2.362169	0.933257		
H	-0.089113	-2.709211	0.25209		
H	1.257231	-2.311951	-0.818863		
O	2.718713	-0.148412	0.95263		
O	-3.739797	-0.527416	1.709354		
O	-2.471654	1.174968	0.993759		
C	-1.418359	-0.682455	2.050811		
C	-2.555567	0.08172	1.524671		
C	-0.056577	-0.18698	1.655777		
H	0.676016	-0.571048	2.371261		
H	-0.042559	0.905029	1.71633		
H	-4.439834	0.051526	1.36907		
C	-1.626852	-2.132938	2.36962		
H	-2.445575	-2.281629	3.075636		
H	-1.889098	-2.685533	1.459921		
H	-0.716217	-2.569227	2.7833		
O	1.731429	0.458439	5.220155		
O	0.514203	2.038579	4.208172		
C	-0.505256	-0.064581	4.728251		
C	0.593107	0.919527	4.675375		
C	-1.656749	0.24624	4.059175		
H	-2.549121	-0.346054	4.229709		
H	-1.78976	1.255445	3.683523		
H	2.405175	1.154998	5.165677		

C	-0.278447	-1.372326	5.419471
H	0.519699	-1.942149	4.931723
H	0.031375	-1.218434	6.457235
H	-1.189874	-1.970497	5.411351
C	3.798298	0.77894	1.187662
C	4.475578	1.167252	-0.124045
H	3.815908	1.764524	-0.758386
H	5.366735	1.76341	0.086618
H	4.780869	0.273771	-0.675868
C	4.762054	0.004289	2.076421
H	5.616632	0.628442	2.346949
H	4.257568	-0.311755	2.993751
H	5.129223	-0.884288	1.556546
C	3.278547	2.009407	1.928211
H	2.588713	2.598568	1.31951
H	2.761744	1.705803	2.842163
H	4.115234	2.65769	2.201407

MAA3-TB-TS-TL

Atom	X	Y	Z		
O	-1.582141	-1.260438	-0.933983	Electronic Energy (EE)	-1152.404778
O	-0.418148	0.355931	-1.938815	Zero-point Energy Correction	0.422997
C	0.538316	-0.718703	0.012617	Thermal Correction to Energy	0.449538
C	-0.510884	-0.450392	-1.053403	Thermal Correction to Enthalpy	0.450482
C	1.786697	0.085498	-0.373549	Thermal Correction to Free Energy	0.365245
H	2.130949	-0.234159	-1.364569		
H	1.525388	1.146374	-0.43339		
H	-2.219672	-1.000397	-1.61553		
C	0.876343	-2.216001	0.030762		
H	1.660258	-2.396652	0.76771		
H	0.010226	-2.829197	0.271083		
H	1.250865	-2.530208	-0.947712		
O	2.775436	-0.156067	0.602715		
O	-3.622542	-0.315074	1.731489		
O	-2.297862	1.199351	0.745386		
C	-1.294042	-0.625198	1.893831		
C	-2.415762	0.180927	1.389208		
C	0.065625	-0.208653	1.410234		
H	0.823889	-0.557113	2.115827		
H	0.106246	0.883606	1.389561		
H	-4.279586	0.343512	1.463452		
C	-1.561359	-2.04185	2.296834		
H	-2.405863	-2.107215	2.9828		
H	-1.821036	-2.639779	1.415683		
H	-0.679821	-2.487142	2.764173		

O	-4.533152	0.534045	5.270989
O	-3.629491	1.913493	3.761442
C	-2.32095	0.098588	4.609893
C	-3.521478	0.946302	4.47892
C	-1.232288	0.431324	3.853194
H	-0.28653	-0.062073	4.050167
H	-1.199374	1.399254	3.36467
H	-5.27139	1.144298	5.133964
C	-2.368575	-1.095177	5.513437
H	-2.542176	-0.79081	6.550018
H	-3.188337	-1.76797	5.245489
H	-1.429802	-1.648273	5.467665
C	3.874422	0.76867	0.659287
C	4.505983	0.953592	-0.719531
H	3.834746	1.472408	-1.408084
H	5.417464	1.549389	-0.631826
H	4.769269	-0.015885	-1.150753
C	4.863737	0.108058	1.610369
H	5.739373	0.744115	1.757782
H	4.392733	-0.066422	2.580619
H	5.191487	-0.852747	1.207158
C	3.407424	2.106777	1.232963
H	2.700924	2.613779	0.572287
H	2.9291	1.952363	2.203473
H	4.262422	2.772991	1.372278

MAA3-TL-TS-TL

Atom	X	Y	Z		
O	-1.228143	-1.349138	-1.037005	Electronic Energy (EE)	-1190.291904
O	-0.538569	0.638674	-1.775535	Zero-point Energy Correction	0.416044
C	0.634343	-0.378569	0.097206	Thermal Correction to Energy	0.441675
C	-0.4188	-0.268551	-0.998575	Thermal Correction to Enthalpy	0.442619
C	1.652092	0.763075	-0.079506	Thermal Correction to Free Energy	0.359095
H	1.090322	1.697625	-0.13555		
H	-1.894759	-1.185793	-1.720641		
C	1.309254	-1.75488	0.015999		
H	2.139454	-1.79834	0.726324		
H	0.613413	-2.561767	0.236098		
H	1.716152	-1.934314	-0.980809		
O	-3.586307	-1.182168	1.537048		
O	-2.626405	0.697962	0.786037		
C	-1.265312	-0.93676	1.848827		
C	-2.523274	-0.378037	1.330165		
C	-0.025647	-0.160516	1.499319		
H	0.756541	-0.402039	2.226612		

H	-0.250851	0.904946	1.590748
H	-4.370458	-0.678687	1.274306
C	-1.201392	-2.407835	2.119293
H	-2.041089	-2.734725	2.732436
H	-1.259895	-2.965928	1.177875
H	-0.265671	-2.673775	2.616676
O	-4.853699	-0.916029	5.068687
O	-4.242161	0.776138	3.741407
C	-2.572793	-0.744401	4.532999
C	-3.936591	-0.200345	4.385272
C	-1.562251	-0.090009	3.88493
H	-0.535799	-0.357059	4.113043
H	-1.743826	0.899573	3.479417
H	-5.711485	-0.490253	4.930213
C	-2.371996	-1.994003	5.333732
H	-2.669061	-1.839105	6.375368
H	-2.986159	-2.815773	4.954273
H	-1.325022	-2.298365	5.313513
H	2.256399	0.809602	0.831801
C	2.579231	0.668878	-1.304794
H	2.885509	1.687162	-1.564222
H	2.021907	0.296075	-2.167879
C	3.833114	-0.158279	-1.112999
C	4.71865	0.119413	-0.067718
C	4.154772	-1.195777	-1.98937
C	5.879738	-0.626428	0.105764
H	4.503527	0.935875	0.614746
C	5.315199	-1.946198	-1.821207
H	3.484104	-1.42155	-2.812896
C	6.180918	-1.666781	-0.769083
H	6.553536	-0.391613	0.922147
H	5.541987	-2.749574	-2.513042
H	7.084815	-2.249411	-0.634701

MMA-C2-IP-TS-IP

Atom	X	Y	Z		
O	0.404912	0.436109	-3.079034	Electronic Energy (EE)	-539.4173945
O	1.615825	1.90312	-1.904454	Zero-point Energy Correction	0.220046
C	2.160619	-0.434944	-1.776661	Thermal Correction to Energy	0.233495
C	1.402143	0.755132	-2.238906	Thermal Correction to Enthalpy	0.234439
C	3.299414	-0.180764	-1.041501	Thermal Correction to Free Energy	0.180617
H	3.996114	-0.979982	-0.818285		
H	3.495366	0.799221	-0.6246		
C	2.059017	-1.696866	-2.604006		
H	1.037636	-2.07104	-2.672279		

H	2.412785	-1.504839	-3.621468
H	2.691953	-2.473287	-2.170947
O	-0.531964	-1.379751	-1.005005
C	0.532121	-0.908465	-0.286944
C	0.307493	0.374078	0.452164
C	1.204734	-2.014709	0.457149
H	1.259717	0.804124	0.770469
H	-0.226779	1.108774	-0.156279
H	-0.294662	0.173392	1.347987
H	2.129126	-1.660333	0.915001
H	1.424568	-2.859677	-0.198912
H	0.539683	-2.368623	1.255281
H	-1.016899	-0.646932	-1.408461
C	-0.391596	1.527429	-3.557851
H	-1.12947	1.082955	-4.221642
H	-0.885945	2.032872	-2.727104
H	0.228727	2.238348	-4.104524

MMA-C3-AI-TS-IP

Atom	X	Y	Z		
O	2.111642	-1.531672	-1.315599	Electronic Energy (EE)	-556.4398702
O	4.260133	-1.338169	-0.716006	Zero-point Energy Correction	0.214869
C	3.085025	0.585522	-1.531677	Thermal Correction to Energy	0.22923
C	3.239883	-0.832487	-1.141419	Thermal Correction to Enthalpy	0.230174
C	4.15198	1.411439	-1.33457	Thermal Correction to Free Energy	0.172605
H	4.139247	2.410177	-1.75825		
H	5.117838	0.989344	-1.078784		
C	3.907717	2.278318	0.72502		
C	3.859496	1.065009	1.605555		
H	4.766838	0.464803	1.527013		
H	3.739805	1.376463	2.650663		
H	2.994607	0.451654	1.340977		
C	2.656418	3.095284	0.585318		
H	2.440015	3.599229	1.535492		
H	2.748	3.856198	-0.191236		
H	1.812979	2.441095	0.356358		
C	5.134331	2.993557	0.697156		
N	6.141002	3.559809	0.62547		
C	2.171114	-2.922588	-0.974871		
H	2.415905	-3.042781	0.081036		
H	1.179291	-3.317765	-1.180401		
H	2.916271	-3.430367	-1.588087		
C	1.768947	1.053692	-2.072424		
H	1.503299	0.502827	-2.979769		
H	0.960002	0.889148	-1.354039		

H	1.81485	2.116681	-2.311768

MMA-C3-AI-TS-TL

Atom	X	Y	Z		
O	2.11166	-1.54387	-1.328743	Electronic Energy (EE)	-556.4331161
O	4.238058	-1.317467	-0.659741	Zero-point Energy Correction	0.215265
C	3.06801	0.584762	-1.525973	Thermal Correction to Energy	0.229744
C	3.231281	-0.831473	-1.120846	Thermal Correction to Enthalpy	0.230688
C	4.133755	1.412804	-1.333781	Thermal Correction to Free Energy	0.17252
H	4.127189	2.407917	-1.765466		
H	5.097745	0.986484	-1.079834		
C	3.917757	2.28579	0.724493		
C	3.850424	1.065318	1.595102		
H	4.72881	0.430076	1.478851		
H	3.772747	1.363359	2.647863		
H	2.959681	0.48327	1.346796		
C	2.675719	3.120819	0.596221		
H	2.45219	3.605098	1.554768		
H	2.781525	3.90194	-0.158082		
H	1.82595	2.484912	0.340035		
C	5.154215	2.987479	0.702369		
N	6.164948	3.544626	0.63138		
C	2.187775	-2.928553	-0.982377		
H	2.403614	-3.045112	0.08044		
H	1.212402	-3.347019	-1.219116		
H	2.965449	-3.424935	-1.563892		
C	1.752341	1.041289	-2.079073		
H	1.503122	0.491886	-2.991445		
H	0.934733	0.859034	-1.375321		
H	1.785534	2.105987	-2.31347		

MMA-C3-CM-TS-IP

Atom	X	Y	Z		
O	-0.282076	1.046607	-0.820339	Electronic Energy (EE)	-770.4122487
O	1.781097	0.431248	-1.427964	Zero-point Energy Correction	0.302318
C	0.696499	-0.707239	0.383858	Thermal Correction to Energy	0.32023
C	0.815227	0.297899	-0.706944	Thermal Correction to Enthalpy	0.321174
C	1.804474	-1.433574	0.704393	Thermal Correction to Free Energy	0.255194
H	1.71016	-2.300529	1.343768		
H	2.711976	-1.339507	0.12073		
O	2.422495	-0.502364	2.452463		
C	2.927151	0.80761	2.415722		
C	1.789732	1.830615	2.31575		

C	3.933244	0.976684	1.27227
H	1.060952	1.6593	3.111849
H	2.167406	2.851642	2.401155
H	1.284593	1.741696	1.351047
H	4.718621	0.220656	1.349726
H	4.394403	1.965774	1.307933
H	3.42834	0.871191	0.310073
C	-0.60039	-0.840633	1.112097
H	-0.849273	0.092637	1.6272
H	-1.420439	-1.051729	0.420087
H	-0.53932	-1.641437	1.848953
C	-0.259948	2.047699	-1.847963
H	-0.128933	1.581151	-2.824606
H	-1.223806	2.546918	-1.79112
H	0.547773	2.756495	-1.66224
C	3.657643	0.9891	3.764105
C	3.734984	-0.033637	4.710256
C	4.267534	2.215505	4.049815
C	4.407608	0.165578	5.915852
H	3.271339	-0.991561	4.510966
C	4.938365	2.412237	5.251484
H	4.223623	3.026188	3.330409
C	5.011851	1.386149	6.19164
H	4.457001	-0.641572	6.638624
H	5.406077	3.36978	5.452826
H	5.53508	1.539827	7.128684

MMA-C3-CM-TS-TL

Atom	X	Y	Z		
O	-0.321175	1.019259	-0.832307	Electronic Energy (EE)	-770.4077639
O	1.783782	0.491629	-1.381938	Zero-point Energy Correction	0.302823
C	0.696723	-0.697706	0.39367	Thermal Correction to Energy	0.320798
C	0.80806	0.313418	-0.691856	Thermal Correction to Enthalpy	0.321742
C	1.815142	-1.408672	0.712593	Thermal Correction to Free Energy	0.255362
H	1.738739	-2.27849	1.349333		
H	2.715246	-1.300759	0.120451		
O	2.417152	-0.482081	2.442627		
C	2.941061	0.816194	2.404551		
C	1.817677	1.855212	2.292707		
C	3.96084	0.968566	1.269656		
H	1.083102	1.693556	3.084454		
H	2.204418	2.8727	2.378437		
H	1.317233	1.769749	1.325293		
H	4.731449	0.199508	1.358858		

H	4.44251	1.947604	1.309861
H	3.467478	0.873422	0.300081
C	-0.596627	-0.844084	1.127251
H	-0.854272	0.087968	1.639013
H	-1.418278	-1.066459	0.441367
H	-0.523192	-1.638187	1.869768
C	-0.303087	2.018534	-1.855709
H	-0.118943	1.562864	-2.829159
H	-1.286051	2.482448	-1.832764
H	0.472007	2.757861	-1.649683
C	3.660837	0.991605	3.759281
C	3.719966	-0.034926	4.701252
C	4.278202	2.210456	4.055578
C	4.382006	0.154077	5.912967
H	3.248577	-0.985063	4.486462
C	4.938932	2.397452	5.263651
H	4.248759	3.023559	3.338397
C	4.99348	1.367891	6.199644
H	4.417814	-0.655557	6.633235
H	5.413016	3.349638	5.473408
H	5.508481	1.513221	7.142129

MMA-C3-IP-TS-IP

Atom	X	Y	Z		
O	-1.364238	-1.155543	-0.45805	Zero-point Energy Correction	0.220219
O	-0.595329	0.900867	-0.021867	Thermal Correction to Energy	0.234158
C	0.883836	-0.981077	0.169539	Thermal Correction to Enthalpy	0.235102
C	-0.394845	-0.297836	-0.098417	Thermal Correction to Free Energy	0.17907
C	1.921284	-0.223464	0.585693		
H	2.906102	-0.660384	0.712804		
H	1.846867	0.857808	0.611532		
C	0.956405	-2.47653	0.031384		
H	0.254466	-2.976696	0.705047		
H	0.707503	-2.796566	-0.984336		
H	1.964204	-2.824153	0.264415		
C	-2.64291	-0.57971	-0.746752		
H	-2.565087	0.122249	-1.577786		
H	-3.286727	-1.413382	-1.017212		
H	-3.036952	-0.068535	0.132444		
O	2.918776	0.849317	3.2764		
C	1.927918	-0.071022	3.046621		
C	2.227755	-1.488812	3.392054		
C	0.566367	0.479813	3.271903		
H	1.461924	-2.148171	2.978113		
H	3.202455	-1.796796	3.000759		

H	2.242638	-1.630939	4.483192
H	-0.18455	-0.169284	2.814375
H	0.475013	1.481804	2.845813
H	0.343085	0.54538	4.347279
H	3.783367	0.42089	3.235755

MMA-C3-TB-TS-IP

Atom	X	Y	Z	Electronic Energy (EE)	-578.7048869
O	-0.298039	1.013556	-0.788598	Zero-point Energy Correction	0.248706
O	1.769675	0.419459	-1.402359	Thermal Correction to Energy	0.263534
C	0.711361	-0.712183	0.428858	Thermal Correction to Enthalpy	0.264478
C	0.810546	0.279521	-0.672636	Thermal Correction to Free Energy	0.206684
C	1.83243	-1.417556	0.755461		
H	1.750819	-2.28312	1.398676		
H	2.731802	-1.322777	0.159359		
O	2.475261	-0.467394	2.455357		
C	2.956264	0.850743	2.378467		
C	3.661661	1.021047	3.741453		
C	1.821171	1.868846	2.267689		
C	3.971383	1.017833	1.249823		
H	4.470281	0.29502	3.846194		
H	2.951519	0.886601	4.560137		
H	4.079889	2.029153	3.798755		
H	1.070297	1.682946	3.04		
H	2.206454	2.884109	2.394851		
H	1.336269	1.817485	1.289508		
H	4.764991	0.271547	1.341359		
H	4.4221	2.012903	1.291507		
H	3.488796	0.90244	0.27688		
C	-0.579392	-0.85249	1.168297		
H	-0.83645	0.082522	1.676198		
H	-1.40257	-1.08159	0.485808		
H	-0.503163	-1.644583	1.913339		
C	-0.292344	2.006521	-1.823541		
H	-0.159224	1.535074	-2.797586		
H	-1.262159	2.49428	-1.766985		
H	0.506853	2.72728	-1.646662		

MMA-C3-TB-TS-TL

Atom	X	Y	Z	Electronic Energy (EE)	-578.6999199
O	-0.327248	0.995989	-0.810118	Zero-point Energy Correction	0.249293
O	1.779601	0.479123	-1.362724	Thermal Correction to Energy	0.264145
C	0.709284	-0.697703	0.431336	Thermal Correction to Enthalpy	0.265089

C	0.809017	0.299657	-0.664886	Thermal Correction to Free Energy	0.207177
C	1.834831	-1.396323	0.756711		
H	1.764074	-2.264868	1.396031		
H	2.728488	-1.292388	0.154012		
O	2.468227	-0.463651	2.447271		
C	2.961289	0.84449	2.378648		
C	3.660687	1.001731	3.746231		
C	1.835333	1.875086	2.268793		
C	3.984985	1.012042	1.255833		
H	4.46095	0.267133	3.849622		
H	2.945364	0.86347	4.559036		
H	4.087614	2.005333	3.81667		
H	1.085163	1.694324	3.042264		
H	2.22597	2.888936	2.391139		
H	1.346871	1.823037	1.292799		
H	4.76752	0.254507	1.346344		
H	4.450113	2.000024	1.308193		
H	3.510053	0.913144	0.277256		
C	-0.580179	-0.84344	1.173453		
H	-0.842113	0.092127	1.676583		
H	-1.404439	-1.079673	0.495386		
H	-0.497384	-1.628708	1.92458		
C	-0.319098	1.983352	-1.844243		
H	-0.133951	1.51893	-2.813402		
H	-1.305418	2.440475	-1.824244		
H	0.450878	2.731037	-1.648966		

MMA-C3-TL-TS-TL

Atom	X	Y	Z		
				Electronic Energy (EE)	-616.5963371
O	-1.460755	-0.584231	-0.46432	Zero-point Energy Correction	0.241142
O	-0.500142	1.368366	0.073176	Thermal Correction to Energy	0.255622
C	0.858335	-0.58927	-0.141947	Thermal Correction to Enthalpy	0.256566
C	-0.398923	0.184412	-0.160482	Thermal Correction to Free Energy	0.196902
C	2.003471	0.097578	0.122061		
H	2.965175	-0.396142	0.036956		
H	1.999597	1.18067	0.096665		
C	4.641319	2.9786	2.733608		
C	3.445609	2.293314	2.588737		
C	3.419968	0.883222	2.560265		
C	4.646326	0.194757	2.667969		
C	5.838752	0.886027	2.81296		
C	5.844207	2.280723	2.847195		
H	4.640474	4.062393	2.75858		
H	2.511109	2.837788	2.497765		
H	4.645304	-0.890438	2.643849		

H	6.770723	0.339394	2.90172
H	6.777684	2.818954	2.960813
C	2.18844	0.175633	2.366969
H	2.16774	-0.896767	2.521561
H	1.251663	0.704756	2.492578
C	-2.719022	0.089289	-0.505627
H	-3.453775	-0.670157	-0.763162
H	-2.951401	0.527458	0.465882
H	-2.707407	0.876492	-1.260594
C	0.807751	-2.080603	-0.283674
H	0.215723	-2.535492	0.516933
H	0.33878	-2.374829	-1.22588
H	1.814955	-2.498571	-0.251235

MMA2-AI-TS-IP

Atom	X	Y	Z		
O	-2.155388	-0.206866	1.895126	Electronic Energy (EE)	-902.2252936
O	-0.768582	1.465283	1.333529	Zero-point Energy Correction	0.344806
C	0.135578	-0.537037	2.261932	Thermal Correction to Energy	0.366848
C	-0.941262	0.351874	1.796575	Thermal Correction to Enthalpy	0.367793
C	1.456682	0.121519	2.545422	Thermal Correction to Free Energy	0.293566
H	1.601931	0.932635	1.829687		
C	-0.235643	-1.809803	2.961941		
H	-0.744415	-1.59402	3.908857		
H	-0.922706	-2.412429	2.365652		
H	0.656268	-2.399911	3.180663		
C	-3.253172	0.588155	1.428588		
H	-3.125392	0.826237	0.372699		
H	-4.140314	-0.023644	1.574917		
H	-3.327453	1.506558	2.012458		
O	0.081957	1.353001	-1.99131		
O	-1.427036	-0.145138	-1.286751		
C	0.85519	-0.366191	-0.601477		
C	-0.281342	0.262878	-1.307157		
C	0.558165	-1.389413	0.253506		
H	1.360704	-1.972858	0.69267		
H	-0.423915	-1.847424	0.212589		
C	2.228691	0.201418	-0.774493		
H	2.53114	0.173811	-1.825817		
H	2.266981	1.249635	-0.461666		
H	2.952216	-0.367047	-0.189206		
C	-0.968779	2.046756	-2.675329		
H	-1.717776	2.391144	-1.960874		
H	-0.492997	2.894666	-3.161924		
H	-1.433545	1.395822	-3.416595		

H	2.261543	-0.606004	2.403177
C	1.611726	0.716238	3.982348
C	1.804098	-0.362678	5.060761
H	0.875467	-0.913814	5.206168
H	2.593993	-1.062398	4.779823
H	2.07143	0.10409	6.011332
C	0.452932	1.650812	4.359748
H	0.327529	2.445483	3.622825
H	-0.473249	1.07271	4.418776
H	0.636449	2.099468	5.338227
C	2.847846	1.517215	3.944345
N	3.816538	2.137181	3.912567

MMA2-AI-TS-TL

Atom	X	Y	Z		
O	-2.153739	-0.2173	1.910127	Electronic Energy (EE)	-902.2146971
O	-0.766954	1.465857	1.372702	Zero-point Energy Correction	0.345391
C	0.135665	-0.545451	2.273866	Thermal Correction to Energy	0.367566
C	-0.942829	0.350759	1.816024	Thermal Correction to Enthalpy	0.36851
C	1.456431	0.114371	2.556127	Thermal Correction to Free Energy	0.293878
H	1.593632	0.922408	1.835467		
C	-0.236709	-1.824409	2.964255		
H	-0.752468	-1.623753	3.910378		
H	-0.924508	-2.419159	2.361359		
H	0.652164	-2.420142	3.182376		
C	-3.240524	0.5584	1.396076		
H	-3.079714	0.769707	0.33944		
H	-4.128695	-0.05464	1.532076		
H	-3.336478	1.491854	1.952696		
O	0.082155	1.347336	-2.012762		
O	-1.438423	-0.106173	-1.240004		
C	0.85112	-0.352419	-0.596588		
C	-0.292549	0.28217	-1.2935		
C	0.553182	-1.377455	0.254847		
H	1.352493	-1.974462	0.681414		
H	-0.434381	-1.822646	0.209744		
C	2.224362	0.211644	-0.781257		
H	2.520974	0.175742	-1.833565		
H	2.261738	1.263696	-0.483496		
H	2.954345	-0.348	-0.195306		
C	-0.974586	2.052006	-2.666486		
H	-1.686712	2.428637	-1.930852		
H	-0.499985	2.878326	-3.19039		
H	-1.49067	1.400161	-3.372441		
H	2.264936	-0.609705	2.412297		

C	1.620549	0.719693	3.986965
C	1.789503	-0.355254	5.073349
H	0.857136	-0.902844	5.208538
H	2.580254	-1.061335	4.811205
H	2.047299	0.111248	6.026316
C	0.469948	1.670222	4.349371
H	0.340936	2.442919	3.590891
H	-0.460831	1.101787	4.427818
H	0.66091	2.143891	5.314313
C	2.870901	1.500886	3.94721
N	3.852212	2.099342	3.913705

MMA2-CM-TS-IP

Atom	X	Y	Z		
O	-1.826265	-0.19356	2.117954	Electronic Energy (EE)	-1116.213592
O	-0.985942	1.568521	1.009619	Zero-point Energy Correction	0.4309
C	0.503059	0.072247	2.098509	Thermal Correction to Energy	0.45702
C	-0.81293	0.571488	1.691106	Thermal Correction to Enthalpy	0.457964
C	1.607862	1.078138	1.978616	Thermal Correction to Free Energy	0.373229
H	1.492777	1.659233	1.059675		
H	1.522331	1.77842	2.826939		
C	0.612526	-0.989327	3.144365		
H	0.466178	-0.542988	4.137544		
H	-0.138982	-1.767146	3.012597		
H	1.605061	-1.440897	3.126795		
C	-3.139308	0.226017	1.725884		
H	-3.220829	0.247677	0.639231		
H	-3.818844	-0.514003	2.142232		
H	-3.358134	1.212286	2.137374		
O	2.858969	0.418939	2.011139		
O	-0.648326	0.713556	-2.372867		
O	-1.616572	-0.798215	-1.033961		
C	0.729491	-0.375561	-0.82204		
C	-0.622005	-0.197448	-1.393286		
C	0.840647	-1.195749	0.259526		
H	1.822128	-1.440405	0.650464		
H	0.0132	-1.838807	0.538989		
C	1.872862	0.415237	-1.378386		
H	2.023305	0.192781	-2.439155		
H	1.684324	1.491087	-1.303485		
H	2.790561	0.178578	-0.838387		
C	-1.930596	0.969465	-2.958994		
H	-2.621587	1.342811	-2.201907		
H	-1.760238	1.726369	-3.720736		
H	-2.329849	0.060475	-3.410027		

C	3.99015	1.255616	2.308858
C	5.194672	0.335158	2.128682
H	6.114584	0.862356	2.389847
H	5.099987	-0.542263	2.772665
H	5.258738	0.005665	1.088182
C	4.069545	2.417405	1.320521
H	5.03316	2.923313	1.404562
H	3.981027	2.024676	0.304763
H	3.281033	3.15551	1.482869
C	3.914095	1.710423	3.768658
C	4.284727	2.991834	4.175908
C	3.496435	0.794714	4.740532
C	4.238874	3.351346	5.523097
H	4.611719	3.725259	3.448308
C	3.443917	1.152532	6.082634
H	3.203551	-0.20454	4.437092
C	3.816792	2.435699	6.479937
H	4.530449	4.35285	5.819927
H	3.112266	0.429974	6.820366
H	3.776686	2.717023	7.526266

MMA2-CM-TS-TL

Atom	X	Y	Z		
O	-1.850308	-0.20445	2.135167	Electronic Energy (EE)	-1116.206569
O	-1.019757	1.570375	1.035777	Zero-point Energy Correction	0.432198
C	0.474317	0.085432	2.128182	Thermal Correction to Energy	0.458211
C	-0.845403	0.57663	1.711855	Thermal Correction to Enthalpy	0.459156
C	1.576021	1.093222	2.003242	Thermal Correction to Free Energy	0.375256
H	1.440419	1.680559	1.091171		
H	1.507865	1.786861	2.858861		
C	0.591233	-0.971226	3.178939		
H	0.469323	-0.522136	4.174024		
H	-0.17452	-1.737398	3.06465		
H	1.578704	-1.43366	3.143011		
C	-3.153814	0.173797	1.686565		
H	-3.190519	0.165271	0.597646		
H	-3.832965	-0.570535	2.096563		
H	-3.41105	1.166074	2.060383		
O	2.826955	0.432114	2.009335		
O	-0.59458	0.704823	-2.389551		
O	-1.611179	-0.762112	-1.034271		
C	0.735976	-0.37126	-0.790376		
C	-0.606045	-0.185769	-1.386741		
C	0.823334	-1.18743	0.295095		
H	1.796361	-1.434298	0.704928		

H	-0.01937	-1.817826	0.557126
C	1.896493	0.407794	-1.3274
H	2.091678	0.151827	-2.372991
H	1.69522	1.483094	-1.300819
H	2.789507	0.195982	-0.73794
C	-1.865099	0.985108	-2.977361
H	-2.54342	1.398669	-2.229538
H	-1.675394	1.71597	-3.760157
H	-2.300642	0.079372	-3.40119
C	3.962242	1.26282	2.291052
C	5.161439	0.340859	2.081699
H	6.088665	0.860103	2.331828
H	5.073824	-0.541006	2.719354
H	5.204124	0.017279	1.038703
C	4.02893	2.43246	1.309185
H	4.995832	2.934561	1.37638
H	3.919666	2.04907	0.292515
H	3.246795	3.172827	1.489937
C	3.916792	1.711134	3.754813
C	4.293526	2.989213	4.164629
C	3.518125	0.78924	4.727763
C	4.274183	3.338601	5.514302
H	4.602935	3.729009	3.436197
C	3.492543	1.136274	6.072475
H	3.216226	-0.205023	4.418053
C	3.872901	2.415756	6.471711
H	4.569555	4.338048	5.812823
H	3.175894	0.408526	6.811123
H	3.85374	2.689146	7.520272

MMA2-IP-TS-IP

Atom	X	Y	Z		
				Electronic Energy (EE)	-885.2126624
O	-2.163045	-0.214445	1.889092	Zero-point Energy Correction	0.350169
O	-0.779578	1.461172	1.331081	Thermal Correction to Energy	0.371541
C	0.128753	-0.538065	2.265152	Thermal Correction to Enthalpy	0.372485
C	-0.948179	0.347344	1.795114	Thermal Correction to Free Energy	0.300279
C	1.44305	0.120693	2.572313		
H	1.597667	0.946702	1.875126		
C	-0.246939	-1.813726	2.958099		
H	-0.752378	-1.600256	3.907396		
H	-0.936697	-2.412346	2.361028		
H	0.64375	-2.407	3.173873		
C	-3.259901	0.577441	1.416893		
H	-3.12828	0.815145	0.361318		
H	-4.146691	-0.035908	1.55949		

H	-3.33949	1.496484	1.999177
O	0.081232	1.3562	-1.990196
O	-1.422665	-0.151972	-1.295896
C	0.856228	-0.357275	-0.594447
C	-0.278972	0.263179	-1.307426
C	0.561222	-1.383772	0.257321
H	1.365126	-1.960921	0.702042
H	-0.416575	-1.850088	0.20836
C	2.226028	0.223926	-0.753102
H	2.538755	0.204189	-1.801613
H	2.251833	1.27058	-0.434047
H	2.949323	-0.34032	-0.163513
C	-0.969798	2.042214	-2.680893
H	-1.725714	2.382032	-1.971505
H	-0.497016	2.893249	-3.165055
H	-1.42582	1.388089	-3.424827
H	2.252442	-0.602044	2.426021
C	1.602129	0.70525	3.994758
O	2.823007	1.459031	3.896045
H	3.01956	1.822701	4.768151
C	1.785908	-0.369722	5.062187
H	0.869174	-0.934391	5.234929
H	2.581358	-1.06298	4.777069
H	2.064642	0.106271	6.007764
C	0.459585	1.644859	4.365043
H	0.36064	2.440325	3.623489
H	-0.490818	1.106822	4.433324
H	0.658282	2.098266	5.340866

MMA2-TB-TS-IP

Atom	X	Y	Z		
O	-1.821602	-0.329959	2.107357	Electronic Energy (EE)	-924.5010554
O	-0.943226	1.524152	1.194795	Zero-point Energy Correction	0.377144
C	0.511447	-0.108786	2.122377	Thermal Correction to Energy	0.40037
C	-0.791892	0.456993	1.766715	Thermal Correction to Enthalpy	0.401314
C	1.642593	0.875223	2.098362	Thermal Correction to Free Energy	0.324051
H	1.537604	1.548601	1.243044		
H	1.568391	1.487805	3.011151		
C	0.593742	-1.26653	3.063305		
H	0.419913	-0.916966	4.090233		
H	-0.153387	-2.027399	2.839191		
H	1.587188	-1.714592	3.030624		
C	-3.125113	0.154151	1.760637		
H	-3.201791	0.295629	0.682631		
H	-3.819796	-0.61397	2.092464		

H	-3.327656	1.093768	2.27647
O	2.870703	0.180368	2.055485
O	-0.538749	1.072002	-2.24263
O	-1.600792	-0.543404	-1.111513
C	0.759458	-0.265911	-0.824291
C	-0.572561	0.046626	-1.383562
C	0.811786	-1.199703	0.16568
H	1.773402	-1.534763	0.538563
H	-0.052044	-1.823247	0.36908
C	1.951203	0.515532	-1.28433
H	2.110821	0.388659	-2.359524
H	1.814495	1.587244	-1.108283
H	2.845275	0.182242	-0.755911
C	-1.797908	1.468253	-2.799706
H	-2.479151	1.77621	-2.005266
H	-1.578294	2.307311	-3.455444
H	-2.236805	0.647538	-3.368033
C	4.054814	0.932317	2.396915
C	5.200051	0.011401	1.998327
H	6.16036	0.469413	2.245116
H	5.12214	-0.940823	2.529518
H	5.173133	-0.1852	0.923279
C	4.088965	1.197173	3.901101
H	3.291063	1.873393	4.216399
H	3.991439	0.257098	4.450834
H	5.040806	1.660053	4.173469
C	4.12259	2.237504	1.607717
H	5.08833	2.717654	1.782846
H	4.023875	2.043895	0.536033
H	3.341688	2.93847	1.912517

MMA2-TB-TS-TL

Atom	X	Y	Z	Electronic Energy (EE)	-924.4936303
O	-1.826769	-0.317644	2.126319	Zero-point Energy Correction	0.378233
O	-0.955857	1.546783	1.223084	Thermal Correction to Energy	0.401452
C	0.502003	-0.07968	2.150861	Thermal Correction to Enthalpy	0.402396
C	-0.80427	0.482691	1.788908	Thermal Correction to Free Energy	0.32539
C	1.635641	0.900817	2.118523		
H	1.506763	1.589896	1.279099		
H	1.590942	1.497814	3.044107		
C	0.592464	-1.232926	3.097282		
H	0.420933	-0.885108	4.125157		
H	-0.156068	-1.994026	2.879817		
H	1.589242	-1.673824	3.060388		
C	-3.120793	0.131602	1.71756		

H	-3.154032	0.236087	0.633455
H	-3.816264	-0.637089	2.047437
H	-3.359435	1.085011	2.191458
O	2.856709	0.1985	2.030391
O	-0.528821	1.038637	-2.276197
O	-1.596657	-0.534159	-1.089905
C	0.75974	-0.242207	-0.797317
C	-0.572515	0.046114	-1.374928
C	0.81512	-1.170669	0.196221
H	1.776958	-1.489315	0.582269
H	-0.049666	-1.795992	0.389423
C	1.945731	0.555175	-1.244885
H	2.150021	0.392979	-2.307476
H	1.767627	1.627563	-1.120833
H	2.824845	0.269307	-0.665952
C	-1.787343	1.420093	-2.831848
H	-2.457453	1.767285	-2.04393
H	-1.572175	2.228011	-3.527505
H	-2.246737	0.580596	-3.355223
C	4.050872	0.920494	2.377347
C	5.177653	-0.000631	1.928075
H	6.15015	0.432868	2.171357
H	5.092586	-0.969094	2.426376
H	5.126924	-0.161782	0.84861
C	4.115498	1.131919	3.890235
H	3.34018	1.815572	4.243342
H	4.000513	0.175838	4.406911
H	5.081366	1.561299	4.167527
C	4.125813	2.253747	1.634741
H	5.097739	2.71988	1.812814
H	4.010943	2.100703	0.558579
H	3.356388	2.952288	1.972069

MMA2-TL-TS-TL

Atom	X	Y	Z		
O	-2.032863	-0.260536	1.993477	Electronic Energy (EE)	-962.3845662
O	-0.657875	1.45398	1.524016	Zero-point Energy Correction	0.371181
C	0.269611	-0.598574	2.279322	Thermal Correction to Energy	0.393803
C	-0.822568	0.312327	1.90238	Thermal Correction to Enthalpy	0.394747
C	1.58497	0.059361	2.564729	Thermal Correction to Free Energy	0.316656
H	1.771063	0.846474	1.831882		
C	-0.054253	-1.879587	2.984621		
H	-0.379561	-1.677975	4.013215		
H	-0.863483	-2.422557	2.495911		
H	0.828806	-2.521262	3.037183		

C	-3.130597	0.545408	1.559277
H	-2.999477	0.822593	0.513806
H	-4.016523	-0.074181	1.680371
H	-3.210416	1.442431	2.17537
O	0.06145	1.396852	-1.964157
O	-1.445253	-0.052065	-1.156749
C	0.867719	-0.344995	-0.621616
C	-0.296354	0.319157	-1.25148
C	0.600973	-1.394693	0.204521
H	1.415663	-1.996774	0.593048
H	-0.391019	-1.832607	0.201957
C	2.237588	0.218596	-0.839613
H	2.500041	0.20804	-1.901468
H	2.290864	1.2616	-0.514387
H	2.982454	-0.3596	-0.291402
C	-1.013037	2.126877	-2.55696
H	-1.687131	2.49915	-1.784089
H	-0.551112	2.956748	-3.086851
H	-1.569403	1.495565	-3.250996
H	2.393237	-0.675599	2.489203
C	1.611818	0.687954	3.976798
H	0.780777	1.39423	4.060954
H	1.463582	-0.091457	4.729202
C	2.91597	1.400692	4.228505
C	3.115982	2.695346	3.742871
C	3.963604	0.769545	4.900315
C	4.331167	3.345143	3.929231
H	2.307155	3.195163	3.217841
C	5.182184	1.41616	5.088218
H	3.820892	-0.235948	5.284576
C	5.369142	2.706474	4.603105
H	4.467837	4.35197	3.551071
H	5.984	0.913072	5.616822
H	6.31586	3.212908	4.751125

MMA3-AI-TS-IP

Atom	X	Y	Z		
O	-2.026371	-0.238856	2.224461	Electronic Energy (EE)	-1248.004202
O	-0.487885	1.266101	1.636498	Zero-point Energy Correction	0.474432
C	0.247702	-0.971929	2.176864	Thermal Correction to Energy	0.504318
C	-0.774172	0.14553	1.997315	Thermal Correction to Enthalpy	0.505262
C	1.546006	-0.392849	2.776361	Thermal Correction to Free Energy	0.413978
H	1.810548	0.493361	2.199791		
C	-0.313151	-2.154015	2.973821		
H	-0.729565	-1.844716	3.932199		

H	-1.106514	-2.650908	2.41355
H	0.484245	-2.879075	3.155732
C	-3.040412	0.750557	1.993921
H	-3.009115	1.08333	0.956284
H	-3.985134	0.25668	2.207383
H	-2.894232	1.598728	2.6639
O	0.060912	1.193141	-1.693403
O	-1.418701	-0.289561	-0.886062
C	0.888084	-0.542181	-0.350119
C	-0.271407	0.112734	-0.977301
C	0.557163	-1.526075	0.736842
H	1.388555	-2.227932	0.854455
H	-0.321391	-2.09422	0.428011
C	2.210636	0.16352	-0.37396
H	2.517576	0.412102	-1.391681
H	2.150465	1.109423	0.176526
H	2.983193	-0.454213	0.0875
C	-1.021626	1.881654	-2.331647
H	-1.72042	2.256575	-1.58216
H	-0.565812	2.710539	-2.86818
H	-1.537248	1.2174	-3.025171
O	-2.009357	-2.823807	-3.225512
O	-0.718189	-1.102868	-3.85012
C	0.117875	-2.680892	-2.257005
C	-0.879349	-2.108133	-3.184312
C	1.247472	-1.944645	-2.034697
H	2.06816	-2.378095	-1.472388
H	1.481751	-1.118906	-2.697185
C	-0.191206	-3.963898	-1.550722
H	-1.103241	-3.879164	-0.95149
H	-0.359158	-4.77199	-2.269468
H	0.634587	-4.24688	-0.896943
C	-3.044006	-2.318477	-4.078111
H	-2.699248	-2.280507	-5.111958
H	-3.872014	-3.016505	-3.981036
H	-3.34716	-1.322866	-3.750913
H	2.341528	-1.128438	2.623019
C	1.571202	0.010549	4.281164
C	1.739916	-1.179353	5.246083
H	0.818266	-1.753633	5.313848
H	2.545145	-1.837647	4.913023
H	1.98137	-0.812905	6.246384
C	0.382079	0.887267	4.709297
H	0.307895	1.785644	4.094981
H	-0.548303	0.320247	4.62453
H	0.498608	1.184383	5.753735

C	2.790092	0.828848	4.42992
N	3.743824	1.461296	4.549242

MMA3-AI-TS-TL

Atom	X	Y	Z		
O	-2.027997	-0.234584	2.209439	Electronic Energy (EE)	-1247.989931
O	-0.475772	1.281912	1.677194	Zero-point Energy Correction	0.475292
C	0.243769	-0.965157	2.186896	Thermal Correction to Energy	0.50527
C	-0.772236	0.160271	2.010084	Thermal Correction to Enthalpy	0.506214
C	1.545281	-0.395822	2.787325	Thermal Correction to Free Energy	0.414838
H	1.801005	0.498135	2.219281		
C	-0.32686	-2.149267	2.973956		
H	-0.741072	-1.846935	3.935415		
H	-1.129644	-2.628209	2.412775		
H	0.459952	-2.887851	3.14949		
C	-3.035123	0.734261	1.897257		
H	-2.957731	1.017023	0.847492		
H	-3.985935	0.24389	2.09221		
H	-2.923443	1.61511	2.530794		
O	0.075532	1.194782	-1.714514		
O	-1.420094	-0.244278	-0.855447		
C	0.886123	-0.516942	-0.338427		
C	-0.273876	0.14187	-0.969138		
C	0.546654	-1.506097	0.741444		
H	1.367377	-2.223339	0.850696		
H	-0.340477	-2.055095	0.422363		
C	2.205992	0.1937	-0.353421		
H	2.511749	0.450094	-1.369237		
H	2.136017	1.138504	0.196948		
H	2.984891	-0.416924	0.107826		
C	-0.996353	1.85516	-2.390747		
H	-1.703446	2.262266	-1.666003		
H	-0.534679	2.660744	-2.957391		
H	-1.502356	1.158974	-3.0589		
O	-2.018205	-2.84102	-3.191594		
O	-0.771254	-1.083085	-3.806831		
C	0.117054	-2.661576	-2.24617		
C	-0.904719	-2.094641	-3.154901		
C	1.235953	-1.906868	-2.036271		
H	2.079375	-2.329267	-1.499771		
H	1.433673	-1.071099	-2.698033		
C	-0.161476	-3.956802	-1.549085		
H	-1.073824	-3.895605	-0.948504		
H	-0.318962	-4.762074	-2.272712		
H	0.671912	-4.231826	-0.901155		

C	-3.073885	-2.328981	-4.005638
H	-2.752893	-2.249167	-5.045016
H	-3.890617	-3.041462	-3.91523
H	-3.385246	-1.347372	-3.645459
H	2.343822	-1.125606	2.618666
C	1.583984	-0.008213	4.295535
C	1.746314	-1.210808	5.245946
H	0.826069	-1.788348	5.303504
H	2.55376	-1.865892	4.911866
H	1.985788	-0.859606	6.251842
C	0.396691	0.866192	4.732335
H	0.318574	1.764019	4.11863
H	-0.534907	0.301093	4.647806
H	0.515735	1.162124	5.776581
C	2.809941	0.801633	4.449886
N	3.770559	1.42153	4.573208

MMA3-CM-TS-IP

Atom	X	Y	Z		
O	-1.725816	-0.244435	2.234416	Electronic Energy (EE)	-1461.997496
O	-0.86508	1.632105	1.388588	Zero-point Energy Correction	0.560544
C	0.637311	-0.185121	1.921586	Thermal Correction to Energy	0.59457
C	-0.712831	0.511661	1.825471	Thermal Correction to Enthalpy	0.595514
C	1.685845	0.90194	2.191918	Thermal Correction to Free Energy	0.493319
H	1.603001	1.704989	1.459106		
H	1.495835	1.330228	3.185668		
C	0.662998	-1.226475	3.043548		
H	0.464102	-0.758927	4.011677		
H	-0.078111	-2.008361	2.875343		
H	1.650456	-1.689771	3.079438		
C	-3.037484	0.316509	2.076529		
H	-3.224291	0.537567	1.025517		
H	-3.725989	-0.44523	2.433797		
H	-3.132015	1.224175	2.673214		
O	2.970936	0.312106	2.142374		
O	-0.645467	1.134082	-2.063858		
O	-1.560502	-0.502182	-0.8288		
C	0.784001	-0.116851	-0.68743		
C	-0.580898	0.137498	-1.172106		
C	0.886032	-0.93198	0.571411		
H	1.887666	-1.363878	0.639334		
H	0.165289	-1.750582	0.527584		
C	1.869671	0.85395	-1.046133		
H	1.966038	0.972391	-2.127197		
H	1.647985	1.848116	-0.641332		

H	2.82556	0.520896	-0.63779
C	-1.949625	1.430213	-2.578477
H	-2.606812	1.751805	-1.768886
H	-1.807173	2.239517	-3.290868
H	-2.368712	0.555463	-3.075463
O	-1.848004	-3.409598	-2.717317
O	-1.081111	-1.582077	-3.760937
C	0.291122	-2.661776	-2.124289
C	-0.919466	-2.474468	-2.950048
C	1.24156	-1.682545	-2.193216
H	2.203225	-1.832745	-1.713197
H	1.195611	-0.954679	-2.995451
C	0.371243	-3.836118	-1.199943
H	-0.452605	-3.830745	-0.479165
H	0.298368	-4.775335	-1.756952
H	1.315897	-3.827578	-0.655073
C	-3.068269	-3.281924	-3.457205
H	-2.870465	-3.339564	-4.528072
H	-3.691617	-4.115336	-3.142357
H	-3.554078	-2.334472	-3.219577
C	4.047619	1.131086	2.628941
C	5.30483	0.322746	2.31626
H	6.188957	0.833278	2.703693
H	5.242819	-0.666236	2.776189
H	5.410453	0.203934	1.234513
C	4.089127	2.462717	1.88131
H	4.038651	2.267064	0.807336
H	3.265033	3.123402	2.158319
H	5.026542	2.983052	2.086887
C	3.91716	1.296278	4.145566
C	3.557154	0.185124	4.915606
C	4.181772	2.500656	4.797092
C	3.456455	0.276993	6.298797
H	3.347301	-0.75698	4.421095
C	4.087582	2.594007	6.186004
H	4.462987	3.380675	4.230666
C	3.722871	1.485434	6.940914
H	3.171126	-0.594832	6.877505
H	4.296724	3.539487	6.674391
H	3.645388	1.55981	8.019839

MMA3-CM-TS-TL

Atom	X	Y	Z		
				Electronic Energy (EE)	-1461.986726
O	-1.751654	-0.288783	2.211917	Zero-point Energy Correction	0.562066
O	-0.900907	1.641132	1.474406	Thermal Correction to Energy	0.596033

C	0.611161	-0.182157	1.949489	Thermal Correction to Enthalpy	0.596978
C	-0.744476	0.506891	1.857769	Thermal Correction to Free Energy	0.495092
C	1.6581	0.904782	2.217691		
H	1.555528	1.714689	1.494629		
H	1.483821	1.323849	3.21831		
C	0.645821	-1.229774	3.065532		
H	0.457384	-0.769825	4.039257		
H	-0.101544	-2.005845	2.900882		
H	1.633779	-1.69304	3.087729		
C	-3.063096	0.237011	1.987434		
H	-3.19164	0.462824	0.929043		
H	-3.751198	-0.546408	2.296484		
H	-3.219587	1.136848	2.583529		
O	2.943561	0.318774	2.141607		
O	-0.591142	1.154388	-2.081541		
O	-1.563933	-0.433108	-0.82374		
C	0.784337	-0.096408	-0.657923		
C	-0.571521	0.178022	-1.165985		
C	0.857904	-0.921123	0.596348		
H	1.851435	-1.371155	0.674149		
H	0.117079	-1.720292	0.534385		
C	1.888259	0.863148	-0.993445		
H	2.032022	0.952834	-2.072121		
H	1.647592	1.868834	-0.631467		
H	2.821909	0.542512	-0.527532		
C	-1.870469	1.436641	-2.652132		
H	-2.56065	1.776809	-1.878168		
H	-1.700935	2.2289	-3.378108		
H	-2.265852	0.547538	-3.142643		
O	-1.843585	-3.418286	-2.685418		
O	-1.111092	-1.564363	-3.710012		
C	0.287818	-2.643802	-2.099016		
C	-0.933439	-2.459838	-2.914121		
C	1.226768	-1.654447	-2.172138		
H	2.2001	-1.79842	-1.7144		
H	1.152896	-0.924119	-2.970004		
C	0.386398	-3.825666	-1.185742		
H	-0.438924	-3.839335	-0.467661		
H	0.323822	-4.760593	-1.750446		
H	1.330521	-3.811834	-0.639681		
C	-3.073574	-3.279092	-3.396607		
H	-2.899122	-3.297132	-4.473193		
H	-3.686743	-4.126429	-3.098269		
H	-3.562785	-2.342738	-3.124057		
C	4.024668	1.134051	2.613291		
C	5.277182	0.328122	2.274429		

H	6.168692	0.831966	2.653027
H	5.219607	-0.663825	2.72684
H	5.364535	0.215633	1.190792
C	4.055243	2.471169	1.872174
H	3.979175	2.283086	0.798821
H	3.240029	3.133372	2.170547
H	4.998854	2.988078	2.0566
C	3.923504	1.294883	4.133088
C	3.5588	0.186092	4.902716
C	4.218078	2.490289	4.786948
C	3.485234	0.270546	6.287159
H	3.320531	-0.745518	4.402204
C	4.151001	2.576478	6.176996
H	4.500568	3.36955	4.220416
C	3.783146	1.469588	6.931157
H	3.195429	-0.599499	6.865602
H	4.38285	3.515228	6.667177
H	3.726612	1.538451	8.011303

MMA3-IP-TS-IP

Atom	X	Y	Z		
O	-2.037023	-0.230821	2.235305	Electronic Energy (EE)	-1230.991598
O	-0.498076	1.264713	1.62825	Zero-point Energy Correction	0.479668
C	0.236328	-0.972278	2.180817	Thermal Correction to Energy	0.508903
C	-0.783064	0.146992	1.999175	Thermal Correction to Enthalpy	0.509847
C	1.529426	-0.400059	2.796379	Thermal Correction to Free Energy	0.42044
H	1.80147	0.498737	2.242132		
C	-0.333391	-2.152708	2.974091		
H	-0.738211	-1.843096	3.937506		
H	-1.135408	-2.640091	2.41762		
H	0.458332	-2.886175	3.148114		
C	-3.047531	0.761322	2.004204		
H	-3.021324	1.087462	0.964226		
H	-3.993457	0.273425	2.226291		
H	-2.894094	1.613587	2.667406		
O	0.058053	1.19336	-1.688566		
O	-1.423514	-0.292069	-0.88989		
C	0.881034	-0.542063	-0.342228		
C	-0.276142	0.111664	-0.974642		
C	0.546202	-1.527199	0.741844		
H	1.377601	-2.228751	0.861099		
H	-0.330885	-2.095226	0.428338		
C	2.20212	0.166199	-0.356408		
H	2.513713	0.420532	-1.371341		
H	2.137001	1.108091	0.200108		

H	2.973197	-0.452345	0.106309
C	-1.022447	1.881106	-2.330676
H	-1.725121	2.254292	-1.583933
H	-0.565489	2.711319	-2.864221
H	-1.534271	1.217171	-3.027353
O	-1.99783	-2.828011	-3.232668
O	-0.709976	-1.102545	-3.851784
C	0.125585	-2.679061	-2.25688
C	-0.869965	-2.108805	-3.186978
C	1.251543	-1.938969	-2.028639
H	2.0714	-2.370239	-1.463478
H	1.485662	-1.111121	-2.688567
C	-0.181605	-3.964023	-1.553115
H	-1.096591	-3.883537	-0.957868
H	-0.342818	-4.772341	-2.273179
H	0.64233	-4.244014	-0.895716
C	-3.031216	-2.324928	-4.087966
H	-2.683664	-2.285527	-5.12084
H	-3.857752	-3.0251	-3.993661
H	-3.337955	-1.330262	-3.761203
H	2.327447	-1.132222	2.635124
C	1.563487	-0.001999	4.285293
C	1.732676	-1.186539	5.236528
H	0.828813	-1.788445	5.319629
H	2.554329	-1.827231	4.905958
H	1.970615	-0.810128	6.236842
C	0.383167	0.868853	4.709004
H	0.327731	1.767193	4.091022
H	-0.566442	0.330383	4.637308
H	0.511805	1.172983	5.752256
O	2.767803	0.789833	4.362955
H	2.914547	1.018312	5.289015

MMA3-TB-TS-IP

Atom	X	Y	Z		
O	-1.723841	-0.300544	2.247466	Electronic Energy (EE)	-1270.285119
O	-0.832229	1.587898	1.46206	Zero-point Energy Correction	0.506559
C	0.638646	-0.272365	1.933251	Thermal Correction to Energy	0.537766
C	-0.698199	0.451198	1.861435	Thermal Correction to Enthalpy	0.53871
C	1.713223	0.782496	2.233129	Thermal Correction to Free Energy	0.443181
H	1.6415	1.616128	1.533267		
H	1.532374	1.174254	3.241672		
C	0.648423	-1.341353	3.029566		
H	0.422906	-0.898146	4.003183		
H	-0.081061	-2.126333	2.827951		

H	1.640305	-1.79426	3.079171
C	-3.025959	0.287785	2.112423
H	-3.210959	0.54975	1.070565
H	-3.72683	-0.474468	2.443583
H	-3.104163	1.174906	2.741534
O	2.979305	0.162198	2.145029
O	-0.613399	1.194662	-2.008892
O	-1.562464	-0.466266	-0.833938
C	0.787843	-0.126444	-0.669681
C	-0.570264	0.16757	-1.150895
C	0.86935	-0.984761	0.561912
H	1.859994	-1.442796	0.616452
H	0.12852	-1.783334	0.491552
C	1.889913	0.839511	-0.990517
H	2.00175	0.983002	-2.067096
H	1.673459	1.826028	-0.565052
H	2.836181	0.486153	-0.57729
C	-1.909266	1.529081	-2.52082
H	-2.566268	1.834625	-1.70488
H	-1.749158	2.358972	-3.205234
H	-2.339437	0.678144	-3.04882
O	-1.891598	-3.304414	-2.806821
O	-1.091666	-1.46028	-3.795499
C	0.259906	-2.612967	-2.191746
C	-0.946475	-2.379383	-3.011665
C	1.225133	-1.646502	-2.226925
H	2.183956	-1.826858	-1.751649
H	1.191017	-0.891556	-3.004402
C	0.321036	-3.818651	-1.307247
H	-0.504418	-3.825861	-0.588398
H	0.236642	-4.737574	-1.895635
H	1.26431	-3.841565	-0.760357
C	-3.109926	-3.132402	-3.540702
H	-2.914188	-3.160099	-4.613147
H	-3.747589	-3.964161	-3.251216
H	-3.57888	-2.184455	-3.273023
C	4.098469	0.887434	2.693787
C	5.313425	0.096315	2.227869
H	6.231319	0.542622	2.616707
H	5.251351	-0.935736	2.582929
H	5.365267	0.08739	1.135893
C	4.024609	0.889207	4.220014
H	3.17629	1.469979	4.588953
H	3.93922	-0.134799	4.593919
H	4.9337	1.333078	4.633652
C	4.149245	2.311378	2.144614

H	4.118369	2.301612	1.051191
H	3.320451	2.919798	2.514517
H	5.080262	2.78975	2.458166

MMA3-TB-TS-TL

Atom	X	Y	Z		
O	-1.980491	0.231215	1.563441	Electronic Energy (EE)	-1270.272186
O	-0.740904	2.074321	1.323726	Zero-point Energy Correction	0.507774
C	0.382394	-0.05268	1.54853	Thermal Correction to Energy	0.539125
C	-0.819638	0.878429	1.476543	Thermal Correction to Enthalpy	0.540069
C	1.542668	0.738054	2.167701	Thermal Correction to Free Energy	0.443448
H	1.663799	1.697757	1.662698		
H	1.293669	0.944028	3.216921		
C	0.099517	-1.29525	2.397153		
H	-0.19632	-1.015063	3.411778		
H	-0.69754	-1.899098	1.963325		
H	1.007713	-1.89775	2.455548		
C	-3.14896	1.023411	1.334015		
H	-3.103525	1.466557	0.339286		
H	-3.989373	0.337016	1.407507		
H	-3.232195	1.807362	2.087836		
O	2.711782	-0.04332	2.060857		
O	-0.025685	2.215867	-2.271728		
O	-1.401529	0.671715	-1.39874		
C	0.930265	0.54402	-0.9312		
C	-0.283874	1.126867	-1.52467		
C	0.702606	-0.526971	0.096522		
H	1.591464	-1.158209	0.165087		
H	-0.133808	-1.15125	-0.226977		
C	2.181619	1.372789	-0.898616		
H	2.514987	1.644846	-1.902556		
H	2.003527	2.314438	-0.367678		
H	2.978075	0.830061	-0.386362		
C	-1.167463	2.84227	-2.857009		
H	-1.852911	3.182799	-2.079413		
H	-0.784872	3.69124	-3.419287		
H	-1.690036	2.15218	-3.521025		
O	-0.472976	-3.722216	-2.473846		
O	1.522015	-3.260503	-1.563955		
C	0.444212	-1.616739	-2.930612		
C	0.581727	-2.921267	-2.246671		
C	1.420555	-0.687046	-2.70695		
H	1.457424	0.203545	-3.325299		
H	2.338523	-0.991441	-2.215912		
C	-0.76976	-1.345699	-3.763658		

H	-0.870651	-2.085893	-4.562545
H	-1.679162	-1.394418	-3.159097
H	-0.706631	-0.352854	-4.212196
C	-0.420245	-5.003529	-1.845221
H	-0.366384	-4.894678	-0.761097
H	-1.339259	-5.511167	-2.129088
H	0.447298	-5.56547	-2.193592
C	3.840735	0.355703	2.853462
C	4.98176	-0.505022	2.326504
H	5.899653	-0.314572	2.887164
H	4.727245	-1.563176	2.419814
H	5.164171	-0.287072	1.271516
C	3.585554	0.044049	4.328646
H	2.786659	0.660526	4.746559
H	3.313414	-1.007705	4.446839
H	4.488391	0.236057	4.913771
C	4.157572	1.837537	2.653842
H	4.258309	2.066332	1.589284
H	3.382938	2.481238	3.077437
H	5.100523	2.083229	3.147985

MMA3-TL-TS-TL

Atom	X	Y	Z		
O	-1.905128	-0.355945	2.250423	Electronic Energy (EE)	-1308.163181
O	-0.424488	1.254839	1.798267	Zero-point Energy Correction	0.501107
C	0.395576	-0.983742	2.186357	Thermal Correction to Energy	0.531419
C	-0.667854	0.102998	2.067263	Thermal Correction to Enthalpy	0.532363
C	1.673396	-0.361823	2.772354	Thermal Correction to Free Energy	0.438059
H	1.954851	0.509644	2.181933		
C	-0.082439	-2.162262	3.041753		
H	-0.419313	-1.837019	4.027209		
H	-0.911979	-2.684475	2.564443		
H	0.741331	-2.86948	3.176856		
C	-2.956449	0.57722	1.98397		
H	-2.895309	0.912249	0.948618		
H	-3.88309	0.034223	2.155126		
H	-2.887074	1.432125	2.657659		
O	0.099256	1.202355	-1.659112		
O	-1.372272	-0.252542	-0.785036		
C	0.947832	-0.53921	-0.344001		
C	-0.229162	0.133753	-0.924344		
C	0.648505	-1.533867	0.743036		
H	1.481183	-2.241164	0.826388		
H	-0.24299	-2.09324	0.453782		
C	2.271485	0.163196	-0.402156		

H	2.549195	0.412388	-1.427985
H	2.225824	1.110718	0.145404
H	3.058279	-0.451185	0.040179
C	-0.992569	1.876585	-2.287424
H	-1.674987	2.271265	-1.532683
H	-0.548343	2.691799	-2.854397
H	-1.521952	1.193715	-2.951149
O	-2.07506	-2.789412	-3.133644
O	-0.822242	-1.042783	-3.768905
C	0.08984	-2.649366	-2.250786
C	-0.950211	-2.058908	-3.122642
C	1.224924	-1.912324	-2.066175
H	2.077794	-2.352212	-1.559382
H	1.414949	-1.072542	-2.725009
C	-0.187292	-3.946439	-1.556642
H	-1.079968	-3.875984	-0.928156
H	-0.378591	-4.743257	-2.28149
H	0.66068	-4.239004	-0.935893
C	-3.14765	-2.253138	-3.908598
H	-2.857781	-2.164632	-4.956433
H	-3.971159	-2.955505	-3.801833
H	-3.434409	-1.272014	-3.527222
H	2.486162	-1.091921	2.686258
C	1.543861	0.076138	4.23907
H	0.658566	0.711673	4.349525
H	1.4037	-0.794403	4.883887
C	2.765866	0.83805	4.689064
C	2.917079	2.187883	4.360713
C	3.788148	0.205658	5.39742
C	4.058252	2.888409	4.73532
H	2.127973	2.691064	3.809386
C	4.933851	0.902621	5.772836
H	3.683224	-0.842392	5.661266
C	5.071796	2.246732	5.443105
H	4.15618	3.93696	4.477611
H	5.716533	0.395738	6.326017
H	5.960931	2.792223	5.737348
